# Galectin-9 regulates dendritic cell polarity and uropod contraction by modulating RhoA activity

**DOI:** 10.1083/jcb.202404079

**Published:** 2025-09-23

**Authors:** Guus A. Franken, Harry Warner, Jorge Cuenca-Escalona, Isabel F. Stehle, Vince P.A. van Reijmersdal, Sophie E. Klomp, Koert Schreurs, Andrea Rodgers-Furones, Rohit Rajesh Gokhale, Manon Vullings, René Classens, Stefania Di Blasio, Yusuf Dolen, Sjoerd van Deventer, Katarina Wolf, Inge M.N. Wortel, Joseph H.R. Hetmanski, Annemiek B. van Spriel, Laia Querol Cano

**Affiliations:** 1 Department of Medical BioSciences, Radboud University Medical Center, Nijmegen, The Netherlands; 2 Data Science, Institute for Computing and Information Sciences, Radboud University, Nijmegen, The Netherlands; 3 Department of Life Sciences, College of Health, Medicine and Life Sciences, Brunel University of London, London, UK

## Abstract

Adaptive immunity relies on dendritic cell (DC) migration to transport antigens from tissues to lymph nodes. Galectins, a family of β-galactoside–binding proteins, control cell membrane organization, exerting crucial roles in multiple physiological processes. Here, we report a novel mechanism underlying cell polarity and uropod retraction by demonstrating that galectin-9 regulates basal and chemokine-driven DC migration in humans and mice. Galectin-9 depletion caused a defect in RhoA signaling that resulted in impaired cell rear contractility. Mechanistically, galectin-9 interacts with and organizes CD44 at the cell surface, in turn modulating RhoA binding to GEF-H1 and the initiation of downstream signaling. Analysis of DC motility in the 3D tumor microenvironment revealed galectin-9 is also required for DC recruitment and infiltration. Exogenous galectin-9 rescued the motility of tumor-immunocompromised human blood DCs, validating the physiological relevance of galectin-9 in DC migration. Our results identify galectin-9 as a necessary mechanistic component for DC motility by regulating cell polarity and contractility, and underscore its implications for DC-based immunotherapies.

## Teaser

Galectin-9 is a critical regulator of dendritic cell basal and chemokine-driven migration by organizing CD44 at the cell surface and promoting RhoA-GEF-H1–mediated signaling. Galectin-9 rescued the motility of tumor-immunocompromised dendritic cells, validating the physiological relevance of our data and underscoring its implications for DC-based immunotherapies.

## Introduction

Dendritic cells (DCs) are the most potent antigen-presenting cell type, paramount for the induction of immune responses against pathogens and tumor cells. DCs are endowed with the capacity to patrol their environment by active migration and to circulate between peripheral tissue and lymphoid organs, thereby linking innate and adaptive responses (Banchereau and Steinman, 1998; Delgado and Lennon-Dumenil, 2022). DC antigen uptake and maturation trigger the upregulation of specific surface proteins such as the chemokine receptor CCR7 that enables CCL19/CCL21-directed chemotaxis to secondary lymphoid organs and costimulatory molecules required for proper T cell activation (Acton et al., 2012; Randolph et al., 2005; Wculek et al., 2020). Rapid DC motility is crucial for their functions, which occurs in a so-called amoeboid manner utilizing a contractile uropod at the cell rear to squeeze through the extracellular matrix without significantly remodeling it (Lämmermann et al., 2008). Amoeboid migration is mediated by the Rho family of small GTPases, key regulators of cytoskeletal dynamics that generate a polarized and dynamic activity balance at the front and rear of the cell (Hind et al., 2016; Sanchez-Madrid and Serrador, 2009). Rac1, Cdc42, and RhoA located at the leading front of the cell drive filamentous actin polymerization (Benvenuti et al., 2004; Lammermann et al., 2009) and directionality, while RhoA is the principal coordinator of the uropod, which choreographs actomyosin contractility (Lammermann et al., 2009; Sanchez-Madrid and Serrador, 2009; Wong et al., 2006). In addition, migrating DCs show enrichment of transmembrane adhesion molecules such as CD44 at the uropod, which link the actin cytoskeleton to the cell membrane, promoting actin polymerization and contraction in a RhoA-dependent manner (Bourguignon et al., 2003; Zhang et al., 2014). Although DC migration dynamics are well characterized, the specific crosstalk between cell membrane events and intracellular cytoskeletal rearrangements that enable front-rear polarization and underlie amoeboid migration remain unresolved.

Galectins are a group of lectins that display a conserved affinity for β-galactoside modifications on cell surface proteins and lipids (Johannes et al., 2018). All galectins contain one or two carbohydrate recognition domains that allow them to simultaneously interact with various glycosylated binding partners, thereby modulating the expression, clustering, and activity of a large range of cell surface proteins (proteoglycans) (Johannes et al., 2018; Liu and Stowell, 2023; Querol Cano et al., 2024). In addition to their extracellular functions, many galectins are also found in the cytosol (Hong et al., 2021; Johannes et al., 2018; Liu et al., 2002; Santalla Mendez et al., 2023) and in the nucleus, where they participate in mRNA splicing (Coppin et al., 2017).

Galectin-9, encoded by the *Lgals9* gene, is a ubiquitously expressed tandem-repeat galectin, known to exert numerous roles in cancer, infection, and inflammation (John and Mishra, 2016; Leffler et al., 2002; Tureci et al., 1997). Galectin-9–mediated functions are cell type–dependent and are dictated by the spatiotemporal expression of its binding partners. Illustrating its versatility, galectin-9 was first characterized as an eosinophil chemoattractant (Matsumoto et al., 1998), induces cell death and immune tolerance by binding T cell immunoglobulin-3 in T helper 1 (T_H_1) cells (Zhu et al., 2005), promotes the expansion of immunosuppressive macrophages (Arikawa et al., 2010) and monocytic myeloid-derived suppressor cells (Dardalhon et al., 2010), and negatively regulates B cell receptor signaling (Cao et al., 2018; Giovannone et al., 2018). Contrary to these reports implying an immunosuppressive role, we and others have previously identified galectin-9 as a positive regulator of DC immune function (Dai et al., 2005; Li et al., 2023; Querol Cano et al., 2019; Santalla Mendez et al., 2023; Suszczyk et al., 2023). Nonetheless, the involvement of galectin-9 in immune cell migration has been insufficiently studied. Dengue virus–infected DCs upregulate galectin-9 expression, which associates with an increased ability to migrate toward CCL19, but whether galectin-9 is relevant for migration in nonpathological naïve DCs has not been addressed (Hsu et al., 2015). Furthermore, very few publications have provided compelling evidence of how endogenous lectins modulate cytoskeleton rearrangements that underlie cell migration, and thus, the mechanism(s) by which galectin-9 shapes cell motility are not delineated.

CD44 is a highly glycosylated single-chain transmembrane receptor with crucial roles in cell adhesion and migration (Senbanjo and Chellaiah, 2017). Illustrating this, CD44-mediated adhesion to hyaluronic acid on the lymphatic endothelium is necessary for DC trafficking to lymph nodes (Johnson et al., 2021). Intracellularly, the cytoplasmic tail of CD44 interacts with ezrin/radixin/moesin or ankyrin to modulate cytoskeletal activation in response to extracellular cues (Bourguignon, 2008). Although the signaling events that control CD44-dependent cytoskeletal rearrangements are well defined (Skandalis, 2023), the molecular mechanisms that regulate CD44 membrane distribution and whether that influences cell migration remain elusive. Interestingly, galectins are required for CD44 nanoclustering and endocytosis at the plasma membrane of epithelial cells, suggesting galectin-mediated interactions are relevant for its spatiotemporal membrane organization (Lakshminarayan et al., 2014).

Here, we show galectin-9 is required for basal and chemokine-driven DC migration *in vitro* and *in vivo*, indicating an evolutionarily conserved function for this lectin. We identified a reduction in RhoA activity, leading to a defect in uropod retraction and actin contractility upon galectin-9 depletion as the underlying mechanism. Importantly, we identified and characterized a functional interaction between galectin-9, CD44, and RhoA at the plasma membrane as an essential driver of DC migration that integrates signals from external stimuli and dictates subsequent cytoskeletal rearrangements. Exogenous galectin-9 was able to rescue the impaired migration capacity of tumor-immunocompromised human blood DCs, confirming the relevance of galectin-9 in DC motility and highlighting the physiological and translational relevance of our findings.

## Results

### Galectin-9 is required for DC migration

**Figure S1.**
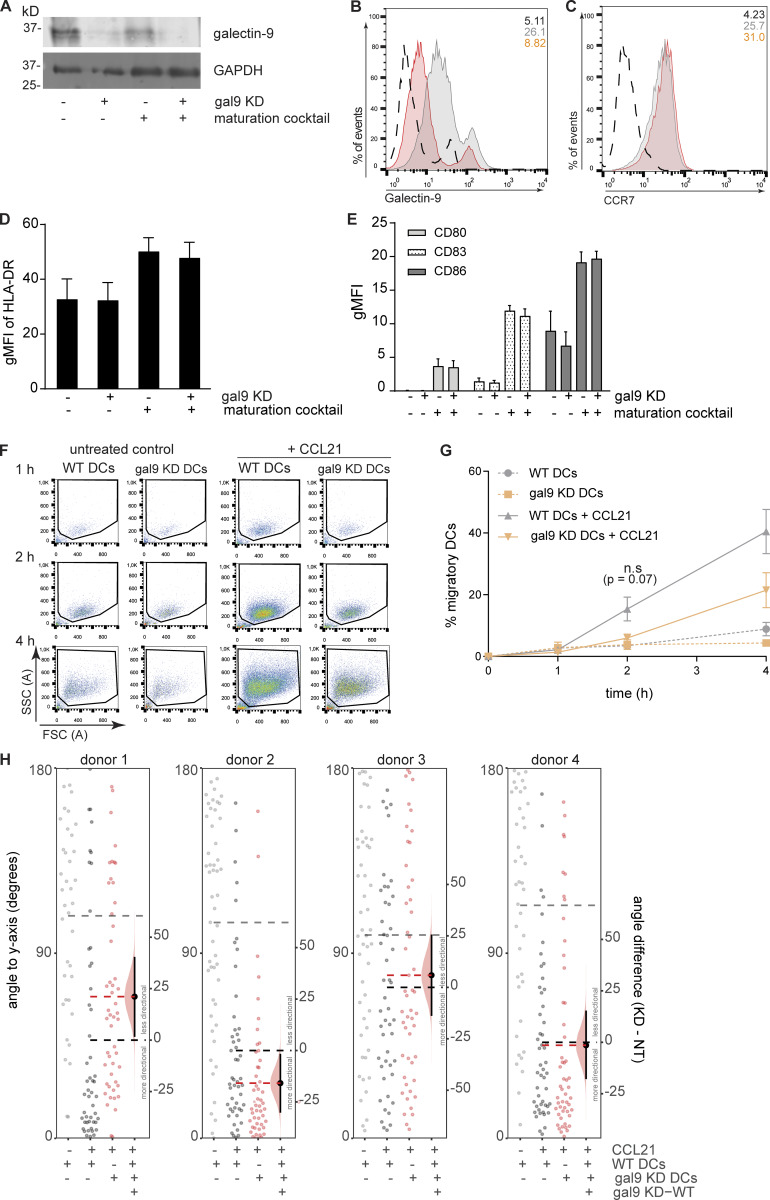
**Galectin-9 depletion impairs chemokine-driven DC migration independently of cell maturation. (A and B)** Galectin-9 expression was assessed by western blot (A) or flow cytometry (B) 48 h in WT or gal9 KD moDCs. Immunoblot is representative of four independent experiments. In B, numbers in the inset indicate gMFI. Cells were gated based on their forward-side scatter after which cell clusters were gated out and only single cells were analyzed. WT, light gray population; gal9 KD moDCs, orange population. A black dotted line represents isotype control values. **(C)** CCR7 surface expression in moDCs treated as per (B). Graph is a representative donor out of three analyzed, and numbers in the inset indicate gMFI. **(D and E)** WT or gal9 KD moDCs were matured and levels of HLA-DR, CD80, CD86, and CD83 analyzed by flow cytometry. The graph depicts the mean ± SEM surface expression (gMFI) of four independent donors. **(F)** WT or gal9 KD moDCs were placed in the upper chamber of a transwell system and subjected to a CCL21 chemokine gradient. Migrated cells were collected at the bottom chamber 1, 2, and 4 h after seeding and measured by flow cytometry. Graphs show a representative donor of four independent donors analyzed. **(G)** Quantification of data shown in F. Graph shows mean percentage ± SEM of migratory DCs relative to the total number of seeded cells for each time point and genotype. An unpaired t test between WT and gal9 KD DCs was performed. **(H)** Overall angle to y axis (degrees) was computed for the overall displacement vector of WT or gal9 KD DCs subjected to CCL21 or nothing as a negative control. Each graph corresponds to one independent donor (labeled donor 1–4; 50 cells analyzed per donor). Horizontal lines represent means for untreated negative control (gray), WT (black), or gal9 KD DCs (red). Plots show the bootstrapped distribution (red) and 95% confidence interval (line segments) of the difference in average angle (gal9 KD-WT). n.s., P > 0.05; *P < 0.05. gMFI, geometrical mean fluorescence intensity. Source data are available for this figure: SourceData FS1.

**Figure 1.**
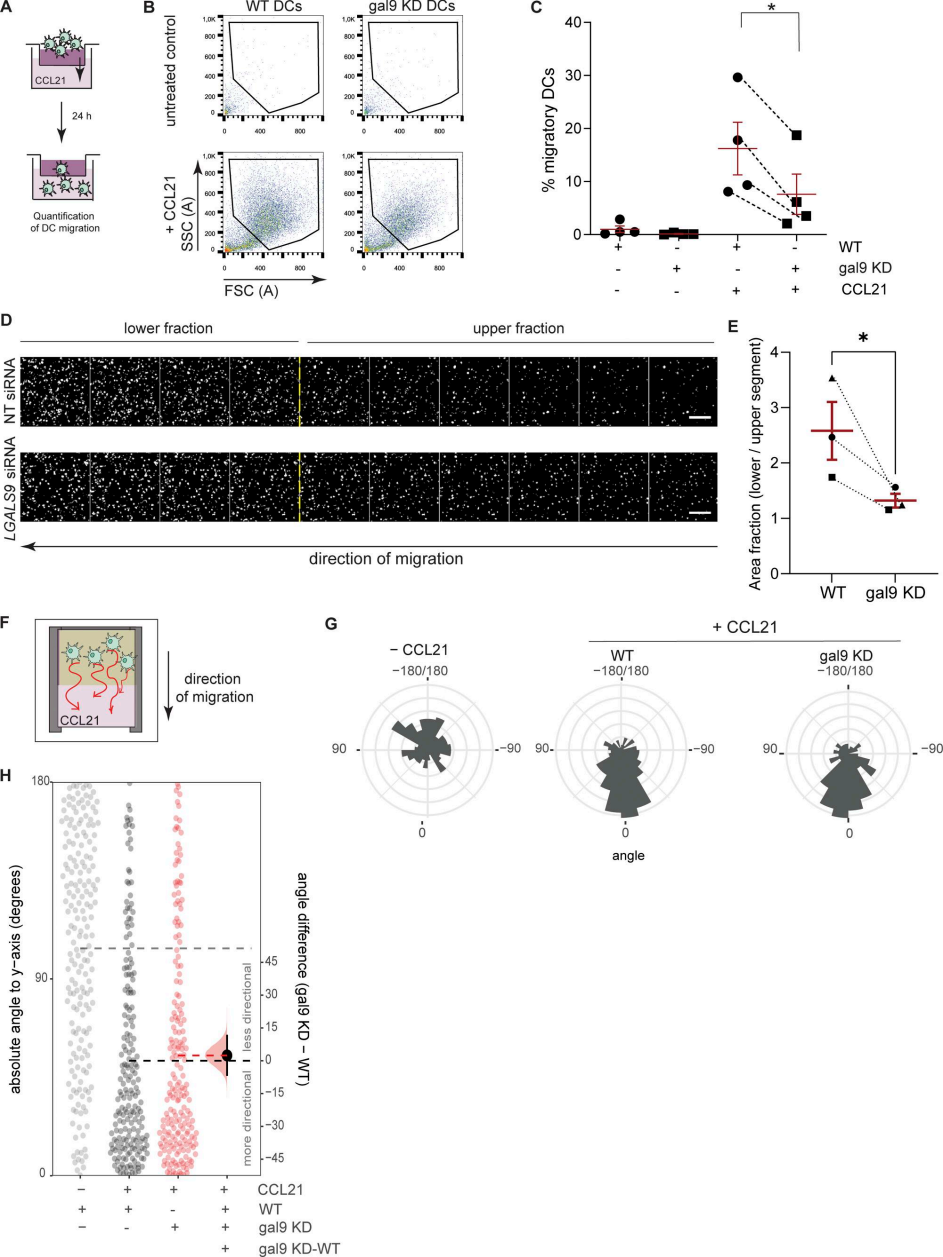
**Galectin-9 depletion reduces DC chemotactic migration. (A)** Schematic representation of the transwell migration assay used. WT or gal9 KD DCs were seeded on a collagen gel in the top chamber of a transwell (5-µm porous membrane) and subjected to a CCL21 chemokine gradient. Migratory cells were collected after 24 h and quantified. **(B)** Day 3 moDCs were transfected with either a *LGALS9* siRNA or a NT siRNA as a negative control, matured at day 6 for 48 h, and subjected to a transwell migration assay as per (A). Representative flow graphs from the migratory moDC fraction are shown. **(C)** Quantification of donors (black symbols) shown in B. Data show the percentage of moDCs that migrated relative to input, and lines connect matched WT and gal9 KD DCs. **(D)** Mature WT or gal9 KD moDCs were stained with CellTrace Far Red, seeded on top of a collagen layer overlaying a transwell chamber, and subjected to a CCL21 chemokine gradient. Cells were left to migrate for 24 h after which collagen gels were fixed and imaged by confocal microscopy. **(E)** Quantification of area fraction signal in WT and gal9 KD DC containing collagens over 10 z-planes from images shown in D from three independent donors (black symbols). **(F)** Schematic representation of the glass migration chamber used to analyze chemotaxis. WT or galectin-9–depleted moDCs were embedded in a collagen gel at the top of a glass chamber, and medium containing CCL21 was placed below. **(G)** Schematic of the migratory angle distribution in WT and gal9 KD DCs in the absence of any chemoattractant (- CCL21) or when subjected to CCL21 (+CCL21). Only WT cells are shown in the −CCL21 condition. **(H)** Overall angle to y axis (degrees) was computed for the overall displacement vector of WT or gal9 KD DCs subjected to CCL21 or nothing as a negative control. Each data point represents 1 cell track pooled from four independent donors with 50 cells analyzed per donor. Horizontal lines represent means for untreated negative control (gray), WT (black), or gal9 KD DCs (red). Plots show the bootstrapped distribution (red) and 95% confidence interval (line segments) of the difference in average angle (gal9 KD-WT). Unpaired Student’s *t* test was conducted to compare WT and gal9 KD cells. *P < 0.05.

Actin polymerization regulates three-dimensional (3D) migration speed in DCs (Renkawitz et al., 2009), and our discovery that galectin-9 mediates actin contractility (Querol Cano et al., 2019) prompted us to study its involvement in governing DC migration. We investigated the functional consequence of galectin-9 depletion in DC migration using nontargeting (NT) siRNA-transfected and *LGALS9* siRNA-transfected monocyte-derived DCs (moDCs), herewith referred to as wild-type (WT) and galectin-9 knockdown (gal9 KD) DCs, respectively. Galectin-9 expression was almost completely inhibited (75–90% reduction; Fig. S1, A and B), while CCR7, HLA-DR, CD80, CD83, and CD86 surface expression showed a similar upregulation upon stimulation demonstrating no impairment in DC maturation upon galectin-9 depletion (Fig. S1, C–E). We first analyzed WT and gal9 KD DC chemokine-driven migration toward CCL21 using a transwell chamber. Galectin-9–depleted DCs displayed an impaired migratory capacity (Fig. S1, F and G). Importantly, this migration defect was not attributed to the aberrant expression of CCL21 receptor CCR7, as this was equal in WT and gal9 KD DCs (Fig. S1 C). We also determined DC chemotactic migration in response to galectin-9 expression in 3D settings by employing transwell assays containing a collagen gel (Fig. 1 A). CCL21 induced DC migration in both WT and gal9 KD DCs, but this was significantly decreased in the latter for all donors analyzed (Fig. 1, B and C). Concomitantly, DCs were trapped in the collagen gel upon galectin-9 depletion (Fig. 1, D and E), demonstrating an involvement of galectin-9 in chemokine-driven DC migration. To discriminate whether galectin-9 depletion altered the migratory capacity of DCs and/or their chemotactic capacity, we employed custom glass 3D migration chambers (Wolf et al., 2013), in which we examined directionality of WT and gal9 KD DCs toward CCL21 (Fig. 1 F). In the absence of chemotaxis (−CCL21), the overall direction of each cell track should be random with an average angle of 90°, whereas a bias to lower angles indicates directionality toward CCL21 (Fig. 1 G). We report the bootstrapped difference of the average angle of gal9 KD cells relative to that of WT DCs (KD-WT) (Ho et al., 2019; Wortel et al., 2022). Validating our model, WT DCs moved in all directions in the absence of any stimulation but exhibited a clear directionality toward CCL21 (Fig. 1, G and H; and Fig. S1 H). Galectin-9–depleted DCs also displayed a directional migration toward the chemokine (Fig. 1, G and H; and Fig. S1 H), indicating that their defective motility is not due to an impairment in their chemotaxis ability.

**Figure 2.**
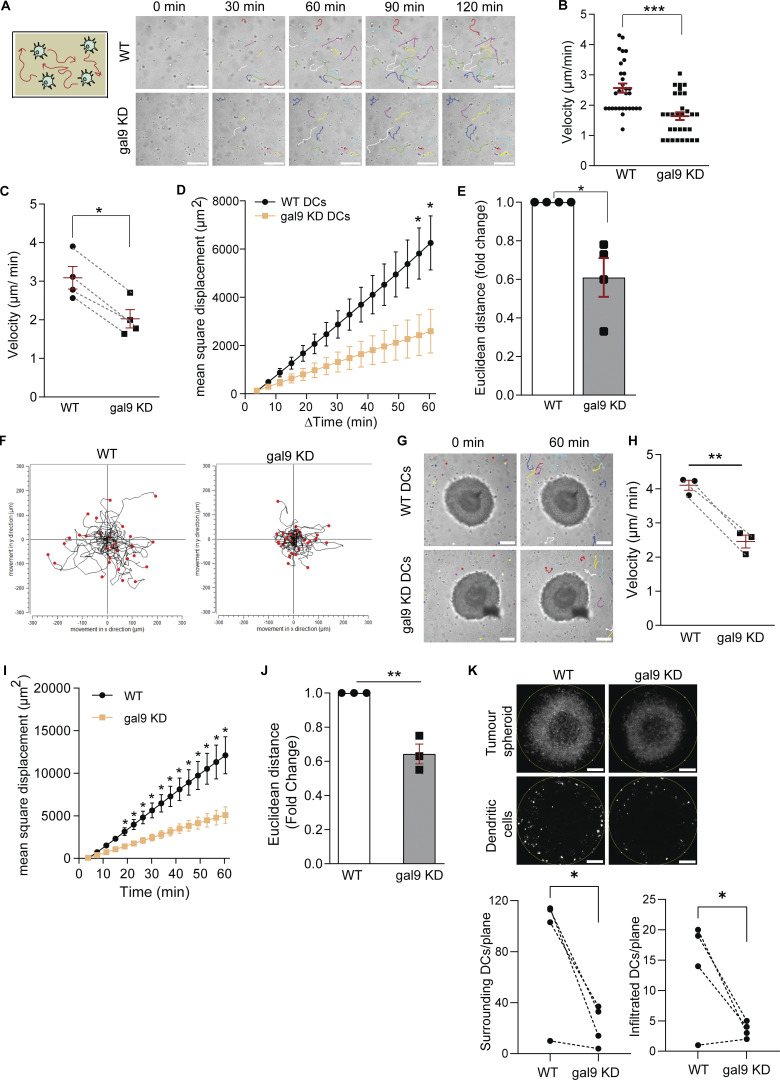
**Galectin-9 depletion reduces DC 3D migration. (A)** Left: schematic representation of the 3D collagen migration assay used. Right: representative single-cell tracking paths of WT or gal9 KD moDCs embedded in a 3D collagen matrix. Lines represent the tracking path covered by each cell from their initial position (time = 0 min). Scale bar = 150 µm. **(B)** Mature WT or gal9 KD moDCs were embedded within a 3D collagen matrix as per (A). The mean cell velocity of a representative donor is shown. **(C–E)** Mean ± SEM cell velocity (C), MSD (D), and Euclidean distance (E) of four independent donors. 20 cells were analyzed per donor and transfection. Lines in C connect matched WT and gal9 KD moDCs. Graph in E depicts relative Euclidean distance in gal9 KD moDCs after 60 min of tracking with respect to control cells. **(F)** Individual trajectory plots of WT and gal9 KD moDCs of one representative donor out of four analyzed. End points of tracks are indicated by red dots. The black line indicates the overall movement in x and y direction (µm). **(G)** Representative single-cell tracking paths of WT or gal9 KD moDCs embedded in a 3D collagen matrix together with a MEL624 malignant melanoma spheroid. Dots indicate cell position at the specified time point, whereas lines represent the tracking path covered by each cell from their initial position (time = 0 min). Scale bar = 150 µm. **(H–J)** Cell velocity (H), the MSD (I), and the Euclidean distance migrated after 1 h (J) are depicted. Data represent the mean value ± SEM of three independent donors. The Euclidean distance is depicted as the mean value for galectin-9–depleted moDCs relative to the control group after 60 min of tracking. A one-way t test was performed. In (H), lines connect matched WT and gal9 KD moDCs. **(K)** Collagen matrices from (G) were fixed and stained for actin, and the number of DCs surrounding (left graph) and infiltrated (right graph) in the spheroid was calculated. Measurements were taken at the same tumor spheroid z-plane across conditions. Images are representative of the tumor spheroid and surrounding DCs. Graphs show the mean ± SEM of four independent donors. Unpaired Student’s *t* test was conducted to compare WT and gal9 KD cells except for E and J, where a one-way *t* test was performed. *P < 0.05; **P < 0.01; ***P < 0.0001.

**Figure S2.**
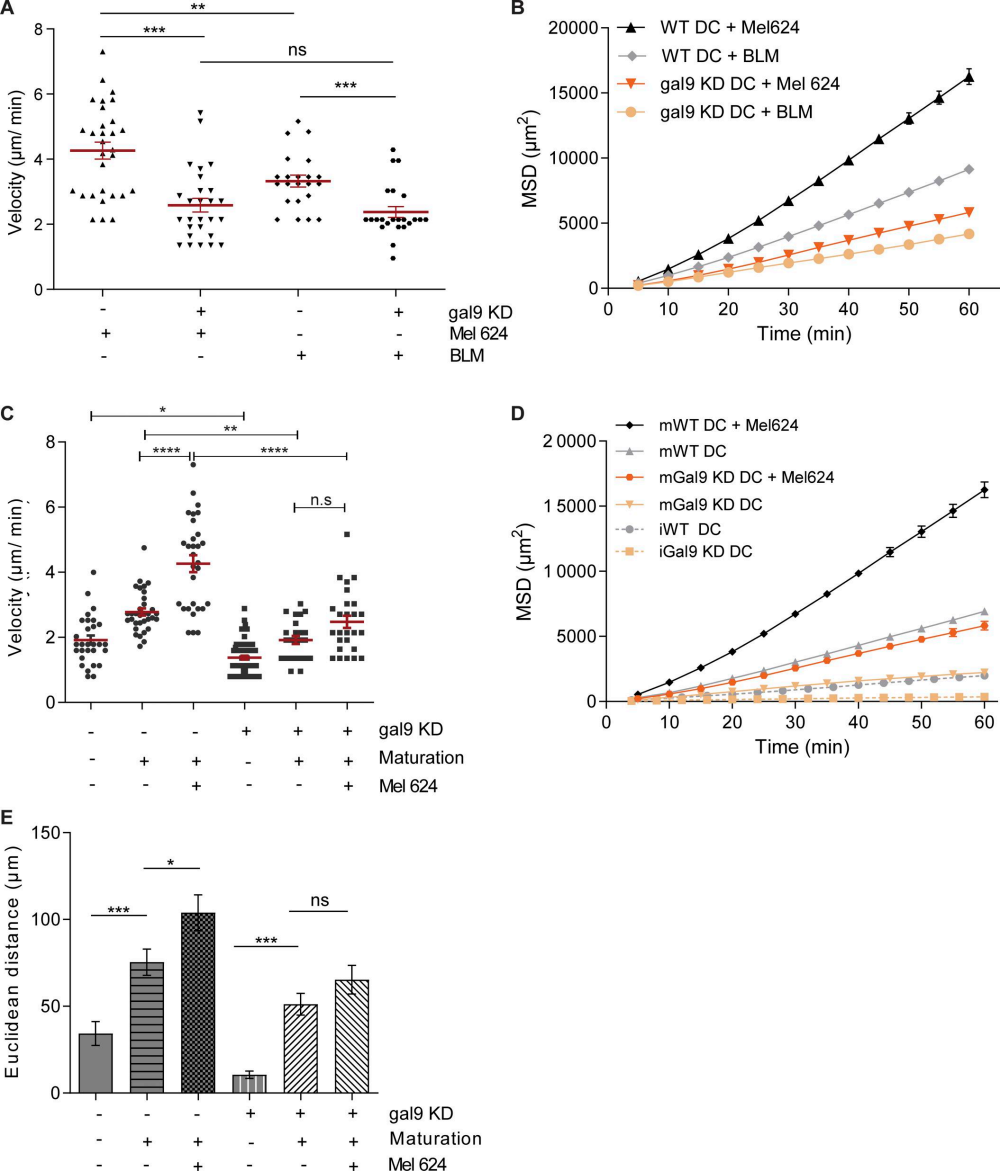
**Galectin-9 depletion diminishes DC 3D migration. (A and B)** WT or gal9 KD moDCs were matured and embedded within a 3D collagen matrix containing either a MEL624 or a BLM melanoma cell spheroid. The median cell velocity (A) and the MSD (B) were calculated. Data represent the mean value ± SEM of a representative donor. At least 20 cells were analyzed per condition. **(C–E)** Migration pattern of WT or gal9 KD moDCs either immature (at day 6 of DC differentiation), mature (at day 8), and mature in the presence of a tumor spheroid (at day 8) was analyzed for one representative donor. The median cell velocity (C), the MSD (D), and the Euclidean distance reached after 60 min of tracking (E) were assessed. In D, m = mature DCs; i = immature DCs. Graphs represent the mean value ± SEM of one representative donor. At least 25 cells were analyzed per condition. One-way ANOVA was performed to compare WT and gal9 KD moDCs. n.s., P > 0.05; *P < 0.05; **P < 0.01; ***P < 0.001; ****P < 0.0001.

**Figure 3.**
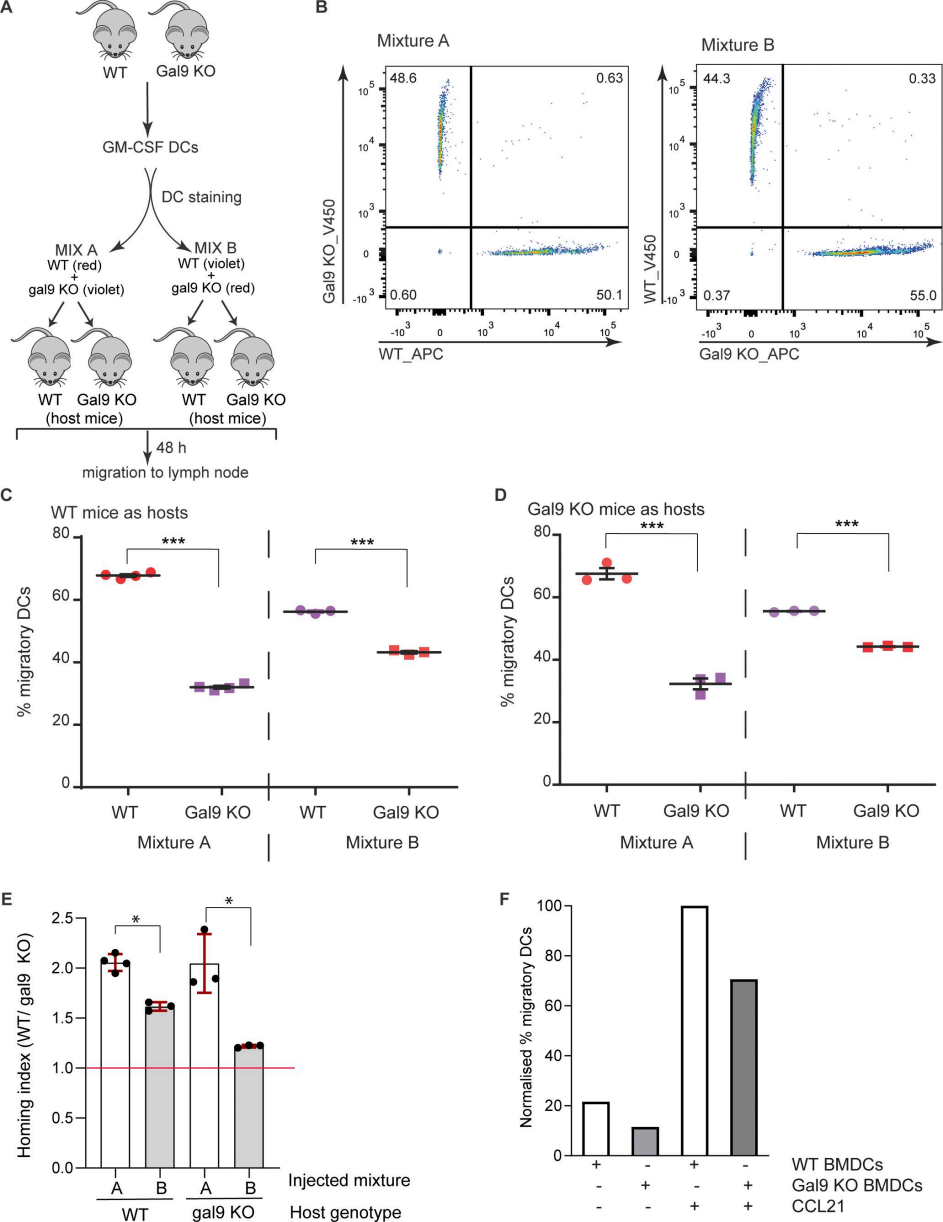
**Galectin-9 function in DC migration is conserved *in vivo*. (A)** Scheme depicting experimental setup. WT and *galectin-9*^−/−^ BMDCs were stained with far-red or violet CFSE dyes, mixed in equal numbers, and injected into the footpad or tail vein of donor mice. 48 h later, draining lymph nodes were isolated and donor BMDCs enumerated by flow cytometry. **(B)** Representative flow cytometry plots depicting the cellular mixtures injected into recipient mice. In mixture A, WT BMDCs were stained with far-red CFSE and galectin-9^−/−^ cells received violet CFSE. For mixture B, colors were interchanged to discard any effect of the dye in cell migration. **(C and D)** DC mixtures from (B) were injected into WT (C) and galectin-9 KO (D) host mice. Data depict the percentage of migratory donor BMDCs ± SEM. Each symbol represents values obtained for one draining lymph node. Statistical significance was determined using one-way ANOVA followed by the Holm–Sidak multiple comparisons test. **(E)** Homing index of WT over galectin-9 KO DCs for each of the injected cell mixtures and host mouse genotype. The homing index was calculated using the following formula: (% far-red signal in tissue/% violet signal in tissue)/(% far-red signal in input/% violet signal in input). Each mixture was injected three times into either WT or gal9 KO host mice. The homing index is depicted. **(F)** BMDCs obtained in A were also subjected to a chemokine transwell assay in the presence or absence of chemokine CCL21. Data show the percentage of moDCs that migrated relative to input. Statistical significance was determined using two-way ANOVA with Tukey’s multiple comparisons test. *P < 0.05; ***P < 0.0001.

Next, we studied the effect of galectin-9 in basal 3D migration using time-lapse microscopy (Fig. 2 A). As shown, the average migratory velocity was significantly impaired upon loss of galectin-9 (Fig. 2, B and C). In addition, we determined the mean square displacement (MSD) as a measurement of particle (i.e., a moDC) confinement within the collagen matrix (Fig. 2 D) and the Euclidean distance as the straight-line distance between the cell starting and end coordinates (Fig. 2 E). Both metrics substantially decreased in gal9 KD DCs compared with WT DCs. Individual cell tracks demonstrate the diminished motility of gal9 KD DCs away from their initial location within the 3D collagen matrix (Fig. 2 F). DCs activate and enhance their motility in the presence of stimuli such as tumors, which in turn secrete specific cytokines that direct DCs toward them (Song et al., 2024). Additional 3D migration assays in the presence of melanoma tumor spheroids were performed to investigate how galectin-9 depletion alters DC function in a physiologically relevant setup (Fig. 2 G). Time-lapse video microscopy analysis demonstrated that also in this context, galectin-9 depletion significantly hampered DC velocity by ∼40% (average speed of 4.1 µm/min in WT *versus* 2.4 µm/min in galectin-9 KD DCs) (Fig. 2 H), as well as their MSD and Euclidean distance (Fig. 2, I and J). This effect was not tumor cell line–specific as 3D assays performed with another melanoma cell line (BLM) yielded similar results (Fig. S2, A and B). Concomitant with a diminished cell velocity, gal9 KD DCs were found in lower numbers in the collagen surrounding the tumor spheroid and displayed a decreased infiltration rate compared with their WT counterparts (Fig. 2 K). WT DCs enhanced their migratory capacity both upon maturation and when present in the vicinity of a tumor, whereas this increase was only marginal in gal9 KD DCs, suggesting that galectin-9 has a broad impact on the capacity of DCs to migrate (Fig. S2, C–E). Next, we sought to investigate whether galectin-9 function in DC migration was evolutionarily conserved using *in situ* migration assays on bone marrow–derived DCs (BMDCs) from WT and *galectin-9*^−/−^ (KO) mice (Fig. 3 A). WT and galectin-9 KO DCs were labeled with far-red or violet carboxyfluorescein succinimidyl ester (CFSE) dyes, mixed in equal numbers, and co-injected into the same footpad or tail vein in host mice (Fig. 3 B). Donor DCs arriving in the draining popliteal and inguinal lymph nodes, respectively, were enumerated 48 h later *via* flow cytometry. To rule out any involvement of galectin-9 present in the recipient animal, both WT and galectin KO were employed as host mice. The number of migratory *galectin-9*^−/−^ BMDCs was significantly reduced compared to WT DCs irrespective of the host genotype (galectin-9 WT or KO) (Fig. 3, C and D). Similar results were obtained in experiments in which violet-labeled WT and far-red–labeled galectin-9 KO BMDCs were employed, ruling out any specific effect of the fluorescent dyes on migration (Fig. 3, C and D). In addition, calculated homing indexes to inguinal and popliteal lymph nodes showed that WT DC motility was around 1.5–2 times higher than that observed for gal9 KO DCs irrespective of the cell labeling or the genotype of the host mice (Fig. 3 E). CCL21 transwell chemotactic assays demonstrated an impairment in murine DC migration upon loss of galectin-9, confirming an evolutionary role of galectin-9 in driving DC motility (Fig. 3 F).

**Figure 4.**
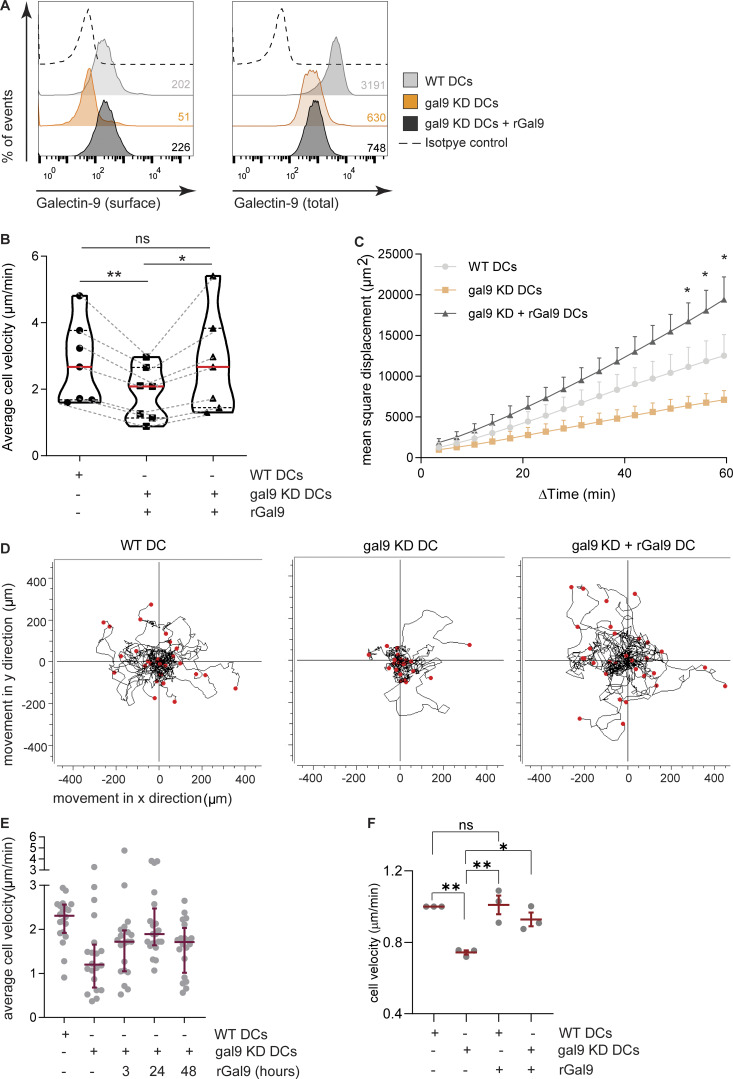
**DC migration relies on membrane-bound galectin-9 fraction. (A)** moDCs were transfected with *LGALS9* or a NT siRNA. 48 h after transfection, gal9 KD cells were treated with 1 µg/ml recombinant galectin-9 (rGal9) protein (gal9 KD + rGal9) or nothing as a negative control for 24–48 h. Surface only (left) and total (right) galectin-9 depletion and treatment with exogenous protein were confirmed by flow cytometry 48 h after treatment. Gray population = WT; orange population = gal9 KD; black population = gal9 KD + rGal9; dotted line = isotype control. Numbers in the inset indicate gMFI. **(B and C)** moDCs treated as per (A) were embedded in 3D collagen matrices followed by time-lapse microscopy imaging to individually track cell migration. At least 20 cells were analyzed for each donor and transfection or treatment. **(B)** Violin plot showing average cell velocity of six individual donors (black symbols). Lines connect paired donors. **(C)** Average MSD ± SEM of five individual donors. **(D)** Individual trajectories of WT, gal9 KD, and gal9 KD + rGal9 moDCs. End points of tracks are indicated by red dots. Data show a representative donor out of six analyzed. **(E)** WT, gal9 KD, and gal9 KD moDCs treated with rGal9 for either 3, 24, or 48 h were embedded in a 3D collagen matrix, and their velocity was calculated. The graph depicts average velocity ± SEM for one representative donor out of 4 analyzed. **(F)** WT and gal9 KD moDCs were treated with rGal9 for 3 h prior to being embedded into 3D collagen matrices, and cell velocity was determined. Data show mean cell velocity ± SEM of three independent experiments (gray symbols). One-way ANOVA with a Bonferroni posttest correction (B, E, and F) or a two-way ANOVA (C) was conducted between WT, gal9 KD, and gal9 KD + rGal9 moDC conditions. n.s., P > 0.05; *P < 0.05; **P < 0.01. gMFI, geometrical mean fluorescence intensity.

Galectin-9 is located both in the cytosol and extracellularly (membrane-bound) in DCs (Querol Cano et al., 2019). To gain further insights into the molecular mechanisms underlying galectin-9 regulation of DC migration, we cultured gal9 KD DCs with exogenous recombinant galectin-9 protein (gal9 KD + rGal9 DCs). Analysis of galectin-9 expression revealed that exogenous protein restored surface-bound levels of galectin-9, while the cytosolic pool remained mostly depleted (Fig. 4 A). We next embedded WT, gal9 KD, and gal9 KD +rGal9 DCs in 3D collagen matrices to characterize their migration capacities. Restoring surface galectin-9 levels rescued the migration deficiency observed in gal9 KD DCs, and no differences were observed in the velocity or the MSD between WT and gal9 KD + rGal9 DCs (Fig. 4, B and C, respectively). Moreover, individual cell tracks illustrate the ability of DCs to migrate upon restoring galectin-9 levels as gal9 KD + rGal9 DCs are indistinguishable from their WT counterparts (Fig. 4 D). Interestingly, the exogenous addition of galectin-9 rescued DC migration also after short incubations confirming the fundamental role of galectin-9 in driving DC migration (Fig. 4 E). Treatment with rGal9 did not enhance WT DC migration in 3D collagen matrices indicating saturating endogenous galectin-9 levels in WT cells and ruling out any contamination of the recombinant galectin-9 protein (Fig. 4 F).

Overall, our results demonstrate that galectin-9 is required for both basal and chemokine-directed migration in DCs. Furthermore, this function appears to be evolutionarily conserved and is likely mediated by the surface-bound fraction of the lectin.

### Galectin-9 controls RhoA-mediated contractility in DCs

**Figure 5.**
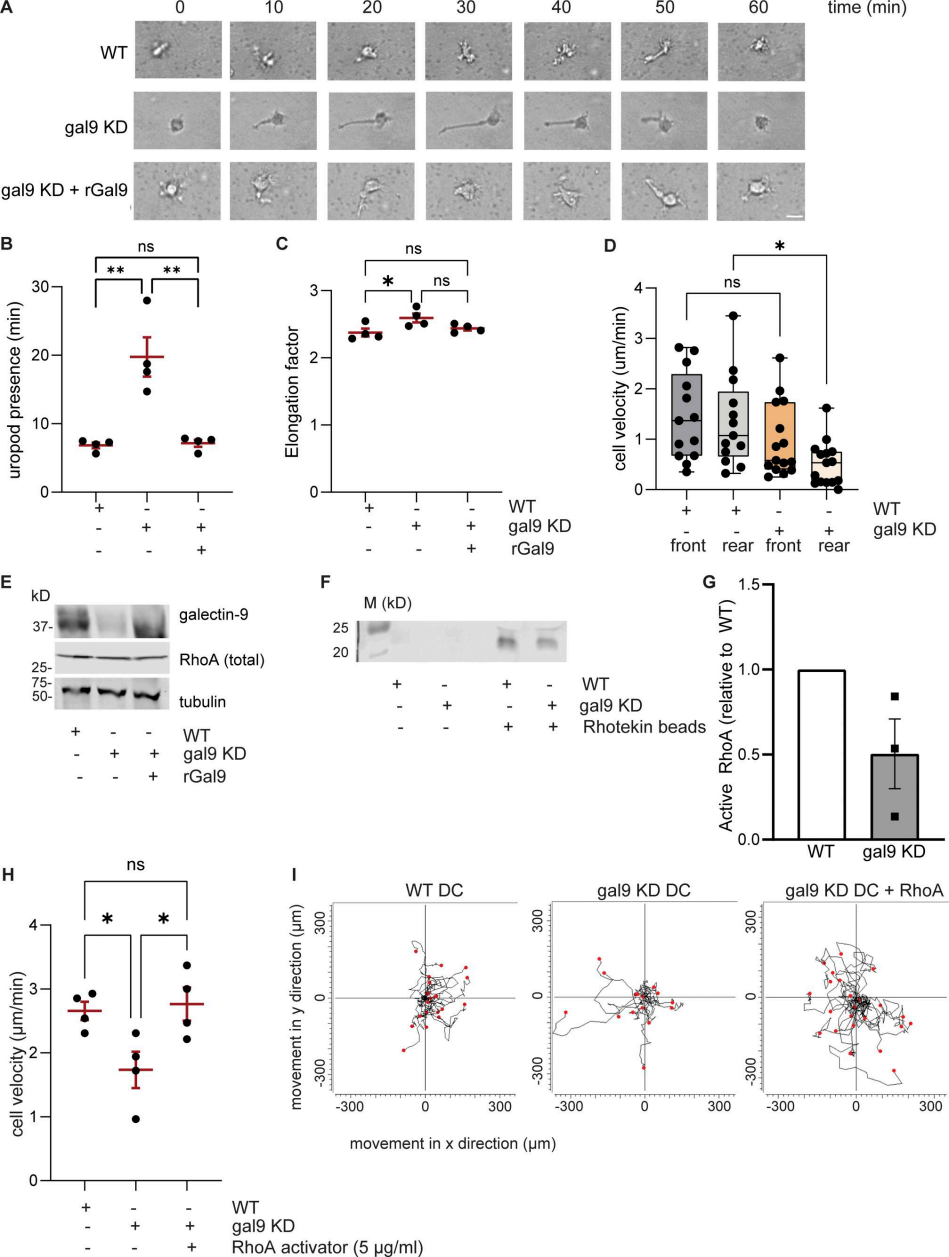
**Galectin-9 regulates uropod contractility. (A)** Time-lapse sequence of representative WT, gal9 KD, and gal9 KD+rGal9 moDCs migrating in a 3D collagen gel. Scale bar = 50 µm. Scale bar = 10 µm. **(B and C)** Mean ± SEM duration (min) of uropod presence (B) and cell elongation factor (C) in WT, gal9 KD, and gal9 KD + rGal9 moDCs for four independent donors (black dots). At least 20 cells were analyzed for each donor and condition. **(D)** Velocity of the front or rear in WT and gal9 KD moDCs. Graphs depict the mean ± SEM for four independent donors (individual cells represented by black symbols). **(E)** Total lysates from WT, gal9 KD, and gal9 KD +rGal9 moDCs were subjected to western blot and galectin-9 and total RhoA expression analyzed. Tubulin was used as a loading control. Immunoblot is representative of four independent donors. **(F)** Levels of active (GTP-bound) RhoA in WT and gal9 KD moDCs detected by immunoblotting. Rhotekin beads alone were used as a negative control (lanes 2 and 3). **(G)** Quantification of data shown in F. The graph depicts relative active RhoA content in galectin-9–depleted DCs (gal9 KD) compared with relevant WT control for three independent experiments. **(H)** WT or gal9 KD moDCs were embedded in 3D collagen matrices prior to being treated with a RhoA activator or PBS as a negative control. The graph depicts mean cell velocity ± SEM for four independent donors (black symbols). At least 20 cells were analyzed for each donor and condition. **(I)** Individual trajectory plots of WT and gal9 KD DCs untreated or treated with the RhoA activator of one representative donor out of four analyzed. End track points are indicated by red dots. The black line indicates the overall movement in x and y direction (µm). One-way ANOVA test with a Bonferroni posttest correction was conducted to test significance. n.s., P > 0.05; *P < 0.05; **P < 0.01. Source data are available for this figure: SourceData F5.

To mechanistically resolve how galectin-9 dictates DC migration, we morphologically characterized migratory WT, gal9 KD, and gal9 KD +rGal9 DCs in a 3D collagen matrix. WT cells contracted the uropod with concomitant forward movement (Fig. 5 A and Video 1), whereas gal9 KD DCs were defective in their ability to contract the cell rear (Fig. 5, A and B; and Video 2). Remarkably, the addition of exogenous galectin-9 protein (rGal9) rescued DC contractility and the uropod could not be detected for abnormal lengths of time in gal9 KD + rGal9 DCs (Fig. 5, A and B; and Video 3). Interestingly, DC elongation was not found to depend on galectin-9 expression (Fig. 5 C), also implying that galectin-9 does not dictate long-term cell length and may be implicated in driving short-lived, dynamic changes in cell shape. Anterograde protrusion at the cell front is functionally dissociated from the retrograde contractility forces that mediate uropod retraction (Lammermann et al., 2008). We did not observe qualitative differences in the leading-edge protrusion formation between WT and gal9 KD DCs, and quantification of the cell front velocity also highlighted no significant differences between both conditions. This is in contrast to the velocity at the cell rear, which was found to be significantly lower upon galectin-9 depletion, in agreement with a retraction defect in those cells (Fig. 5 D).

**Video 1.** **Representative time-lapse confocal microscopy of a WT moDC randomly migrating on 3D collagen matrices.** The movie was acquired in a BD Pathway 855 spinning disk confocal microscope (BD Bioscience) using a 10 × 0.3 NA air objective equipped with cameras and custom-built climate chambers (37°C, 5% CO_2_, humidified). Images were acquired with a time interval of 2 min.

**Video 2.** **Representative time-lapse confocal microscopy of a gal9 KD moDC randomly migrating on 3D collagen matrices.** The movie was acquired in a BD Pathway 855 spinning disk confocal microscope (BD Bioscience) using a 10 × 0.3 NA air objective equipped with cameras and custom-built climate chambers (37°C, 5% CO_2_, humidified). Images were acquired with a time interval of 2 min.

**Video 3.** **Representative time-lapse confocal microscopy of a galectin-9 KD moDC pretreated with rGal9 protein for 48 h randomly migrating on 3D collagen matrices.** The movie was acquired in a BD Pathway 855 spinning disk confocal microscope (BD Bioscience) using a 10 × 0.3 NA air objective equipped with cameras and custom-built climate chambers (37°C, 5% CO_2_, humidified). Images were acquired with a time interval of 2 min.

**Figure S3.**
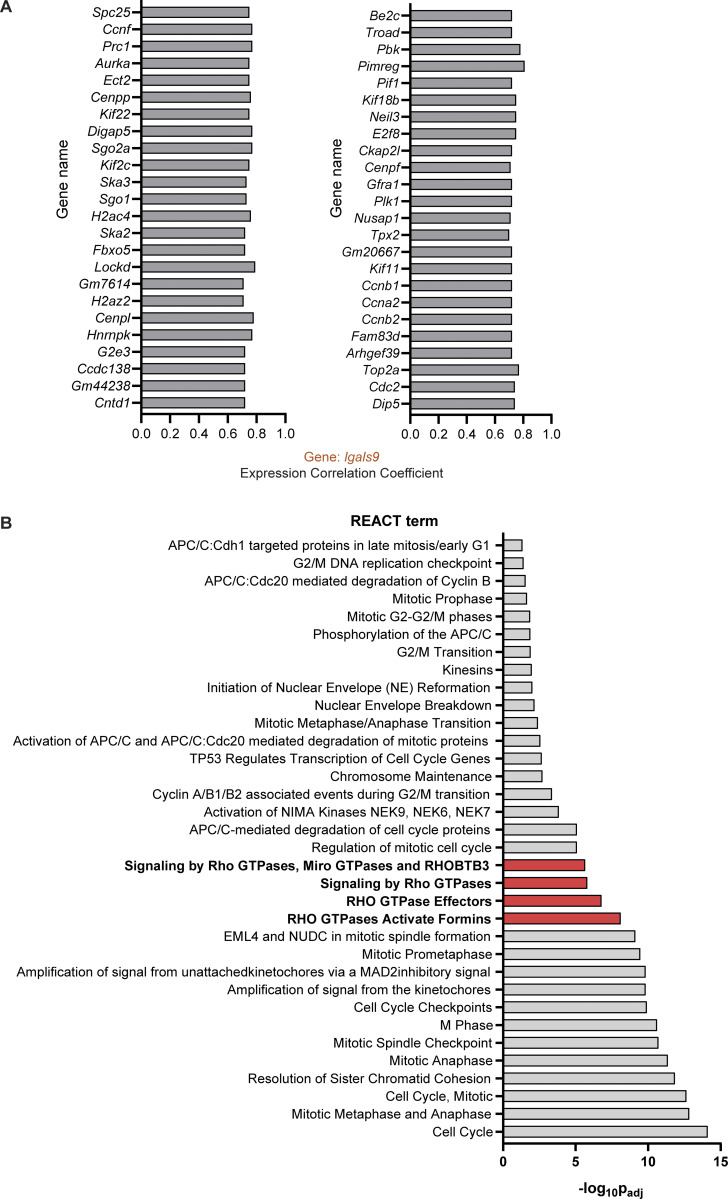
**RhoGTPase-mediated pathways positively correlate with *lgals9* expression. (A)** 50 genes that positively correlated to *lgals9* gene across all immune cell types (GSE109125 dataset) grouped by secondary correlation. **(B)** Functional pathway enrichment analysis (Reactome dataset). Data are shown as −log_10_ of the adjusted P value.

**Figure 6.**
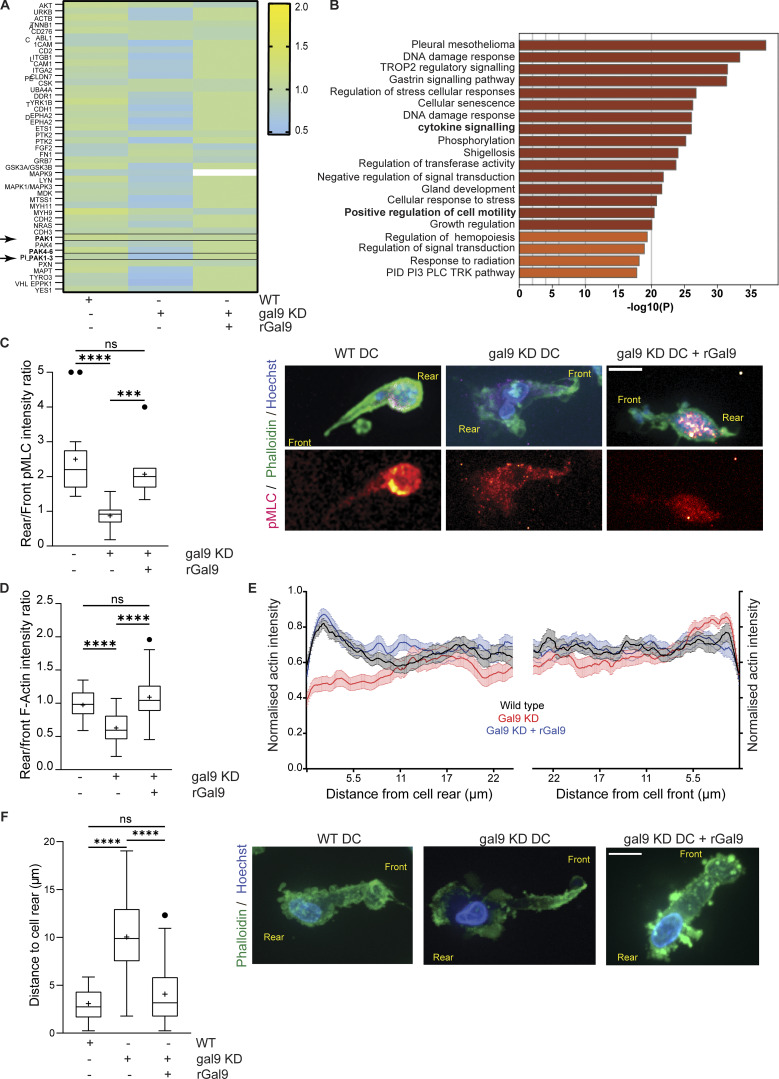
**RhoA downstream signaling is impaired in galectin-9–depleted DCs. (A)** Heat map of proteins involved in cytoskeletal rearrangements analyzed by RPPA. Whole-cell lysates obtained from mature WT, gal9 KD, or gal-9 KD +rGal9 DCs were denatured, arrayed on nitrocellulose-coated slides, and probed with antibodies against the specified protein targets. Data shown represent mean protein expression normalized against the loading control. **(B)** Metascape pathway enrichment analysis from all proteins found to be differentially regulated by RPPA between WT and gal9 KD moDCs. **(C and D)** WT, gal9 KD, or gal9 KD + rGal9 mature DCs were embedded in 3D collagen gels and stained for pMLC, actin (phalloidin), and nucleus (Hoechst). Box-and-whisker plots show mean (as +) pMLC (C) or actin (D) intensity ratio at the rear over the front of the cell (based on the nucleus localization) for 4 independent donors (10–15 cells analyzed per donor). Whiskers are generated using the Tukey method. Representative high-resolution Airyscan images of WT, gal9 KD, or gal9 KD + rGal9 moDCs are shown. Scale bar = 10 µm. Two-way ANOVA followed by Dunnett’s test for multiple comparisons was performed in all panels. **(E)** Average actin intensity for the entire width of WT (black), gal9 KD (red), and gal9 KD + rGal9 (blue) moDCs at each position forward from the rearmost point of the cell (left) and backward from the frontmost point of the cell (right) for the rear and front 25 μm. 15 cells were analyzed across three independent donors with bar representing SEM; all values were normalized to the maximum actin intensity of each cell. **(F)** Distance of the nucleus to the rear of the cell in WT, gal9 KD, and gal9 KD + rGal9 moDCs. Box-and-whisker plot shows mean (as +) nuclear distance for four independent donors (10–15 cells analyzed per donor). Whiskers are generated using the Tukey method. Representative high-resolution Airyscan images of WT, gal9 KD, or gal9 KD + rGal9 moDCs stained for actin (phalloidin) and nucleus (Hoechst) are shown. Scale bar = 10 µm. Significance was determined by one-way ANOVA with Tukey’s multiple comparisons test. n.s., P > 0.05; ***P < 0.001; ****P < 0.0001. n.s., P > 0.05; *P < 0.05; ***P < 0.01; ****P < 0.001.

**Figure S4.**
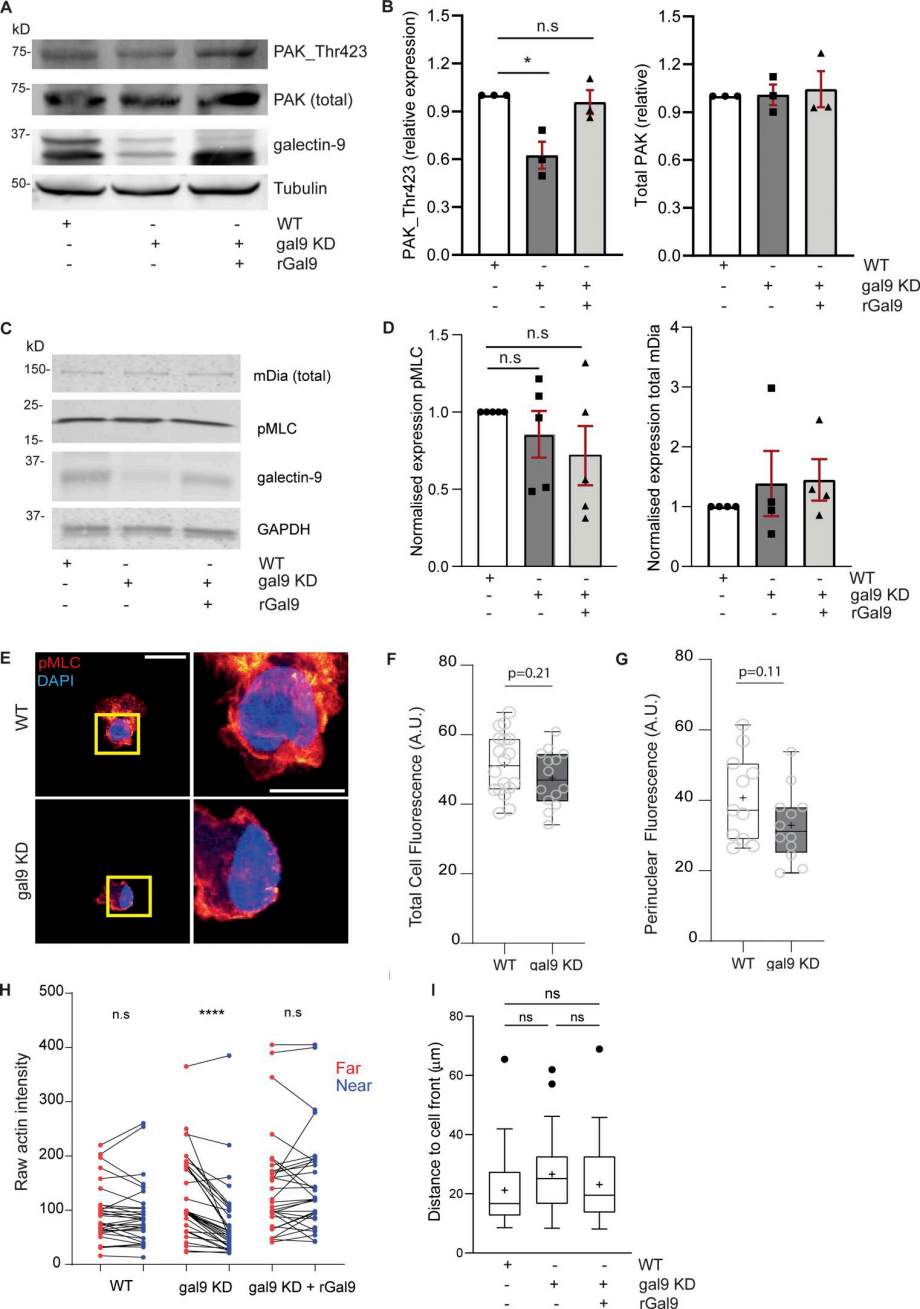
**Galectin-9 depletion alters actomyosin cytoskeleton. (A)** Total lysates from WT, gal9 KD, and gal9 KD + rGal9 moDCs were subjected to western blot and total PAK1, PAK_Thr423, and galectin-9 protein expression analyzed. Tubulin was used as a loading control. Immunoblot is representative of three independent experiments. **(B)** Quantification of mean ± SEM of PAK1_Thr423 and total PAK1 content normalized to corresponding tubulin for three independent donors. One-way ANOVA was performed. **(C)** Total lysates from WT, gal9 KD, and gal9 KD +rGal9 moDCs seeded onto 6-well plates were subjected to western blot and total mDia, pMLC (Ser19), and galectin-9 expression analyzed. Tubulin was used as a loading control. Immunoblot is representative of five independent experiments. **(D)** Quantification of normalized mDia and pMLC content shown in C. A one-sample t test was performed. n.s., P > 0.05; *P < 0.05. **(E)** WT or gal9 KD mature DCs were seeded onto coverslips and stained for pMLC and nucleus (DAPI). ROI in the left panel (yellow square) is depicted in the zoomed right panels. Scale bar = 10 µm; insets = 10 µm. **(F and G)** Quantification of data shown in (E). Box-and-whisker plots show mean (as +) pMLC intensity ratio in the whole cell (F) or at the perinuclear region (G) for two independent donors (10–15 cells analyzed per donor). Whiskers are generated using the Tukey method. Significance was determined by an unpaired *t* test. **(H)** Pairwise comparison of actin intensity at the cell front (red) and rear (blue) of 30 WT, gal9 KD, or gal9 KD + rGal9 moDCs from three independent donors, corresponding to rear/front ratio analysis shown in Fig. 6 D. **(I)** Distance of the nucleus to the front of the cell in WT, gal9 KD, and gal9 KD + rGal9 moDCs. The box-and-whisker plot shows mean (as +) nuclear distance for four independent donors (10–15 cells analyzed per donor). Whiskers are generated using the Tukey method. Significance was determined by one-way ANOVA with Tukey’s multiple comparisons test. n.s > 0.05. In H, two-tailed paired Student’s parametric *t* test was used. Source data are available for this figure: SourceData FS4.

RhoA activity governs uropod contraction (Hind et al., 2016; Meili and Firtel, 2003), and thus, we next examined whether RhoA-mediated signaling was altered upon galectin-9 depletion. Although total RhoA levels did not differ across all conditions (Fig. 5 E), RhoA GTPase activity was markedly decreased in gal9 KD DCs (Fig. 5, F and G) in agreement with the aforementioned impairment in uropod retraction. Pretreatment of gal9 KD DCs with the RhoA activator II prior to being embedded into collagen gels restored their impairment in cell velocity (Fig. 5 H). Individual cell tracks demonstrate the rescue in DC migration upon restoration of RhoA activity, confirming 3D migration defect induced by galectin-9 depletion is due to defective RhoA activation (Fig. 5 I). To further confirm the link between galectin-9 and RhoA, we sought to investigate which genes positively correlate with *lgals9* expression and the pathways they mediate. We obtained a list of the top 50 genes correlating with *lgals9* using the ULI RNA-seq dataset (GSE109125) from the Immunological Genome Project (ImmGen) and performed a functional pathway enrichment analysis using the Reactome dataset (Griss et al., 2020) (Fig. S3 A). Data obtained showed a significant enrichment in both the Rho GTPase effectors and signaling by Rho GTPase pathways confirming a correlation between galectin-9 and RhoA activity (Fig. S3 B). Reverse-phase protein array (RPPA) (Siwak et al., 2019) performed against >450 key functional proteins on WT, gal9 KD, and gal9 KD + rGal9 DC whole-cell lysates revealed a striking decline in either the expression or activity of proteins involved in cytoskeleton rearrangements in gal9 KD cells compared with WT or gal9 KD + rGal9 DCs (Fig. 6 A). Remarkably, minimal differences were detected between WT and gal9 KD + rGal9 DCs, indicating that treatment with exogenous galectin-9 protein rescues the DC signaling signature (Fig. 6 A). Enrichment pathway analysis (Zhou et al., 2019) performed on the differentially expressed proteins confirmed galectin-9 is a positive regulator of cell motility (Fig. 6 B). Validating our analysis, cytokine signaling was also found to be positively regulated in WT compared with gal9 KD DCs, which we have previously reported (Santalla Mendez et al., 2023). RPPA analysis revealed that the active form of P21-activated kinase 1 (PAK1) (PAK_Thr423), an activating Ser/Thr kinase downstream of RhoA, is downregulated in gal9 KD DCs, whereas treatment with exogenous galectin-9 protein (gal9 KD + rGal9 DCs) rescued its levels to those found in WT cells (Fig. 6 A). Total levels of PAK1 did not differ across conditions, suggesting galectin-9 is not involved in regulating its expression. Western blot analysis of phosphorylated and total PAK1 levels in multiple donors validated our RPPA data and RhoA-mediated signaling to be differentially activated in response to galectin-9 cellular levels (Fig. S4, A and B). Through immunoblotting of 2D-seeded moDCs, we were unable to observe differences in total phospho-myosin light chain (pMLC) or mDia expression upon galectin-9 depletion with or without rGal9 treatment (Fig. S4, C and D). Immunofluorescence against pMLC showed subtle (not significant) differences in pMLC localization or intensity between WT and gal9 KD moDCs seeded onto coverslips (Fig. S4, E–G), suggesting that changes in pMLC might be masked by stiffness of the 2D culture (Yang et al., 2017; Lee and Kumar., 2016; Caswell and Zech., 2018). Since western blot only provides the average total expression and to circumvent mechanoresponses induced by plastic or glass-adhered cells, we employed Airyscan microscopy of collagen-embedded moDCs to examine spatial differences in MLC phosphorylation with respect to galectin-9 expression in 3D. Myosin II activity prominently accumulated at the rear of migrating WT DCs, but this was largely abrogated upon galectin-9 depletion, indicative of impaired generation of contractile forces (Fig. 6 C). In agreement with the rescue previously observed in uropod retraction, treatment with rGal9 was sufficient to restore pMLC activity at the cell rear of galectin-9–depleted DCs (Fig. 6 C). We further characterized the architecture of the actomyosin cytoskeleton in collagen-embedded DCs by structured illumination microscopy (SIM) super-resolution and Airyscan microscopy and observed a decreased F-actin intensity at the cell rear in gal9 KD compared with WT DCs (Fig. 6 D, Fig. S4 H, and Video 4). Cell-wide line profile analysis of actin staining revealed that actin intensity was specifically reduced at the cell rear in gal9 KD cells compared with WT and gal9 KD treated with rGal9 counterparts, while the actin intensity at the leading edge was slightly higher in gal9 KD cells, further suggesting a specific rear retraction defect (Fig. 6 E). Interestingly, the cell rear in gal9 KD DCs was further away from the edge of the nucleus than in WT and rGal9-treated DCs further substantiating a rear retraction defect upon galectin-9 depletion (Fig. 6 F and Fig. S4 I). Overall, our data show that galectin-9 regulates uropod contractility by modulating RhoA retrograde activity, directly altering the architecture and dynamics of the actomyosin cytoskeleton at the cell rear.

**Video 4.** **Representative actin staining (phalloidin) for WT (left cell) and galectin-9–depleted (right cell) DCs using a Nikon Ti2 spinning disk confocal microscope with Crest Optics SIM module for super-resolution imaging.** Images were captured using a Teledyne Photometrics Kinetix camera through a 100×/1.45 DIC Plan Apo oil-immersion objective.

**Figure 7.**
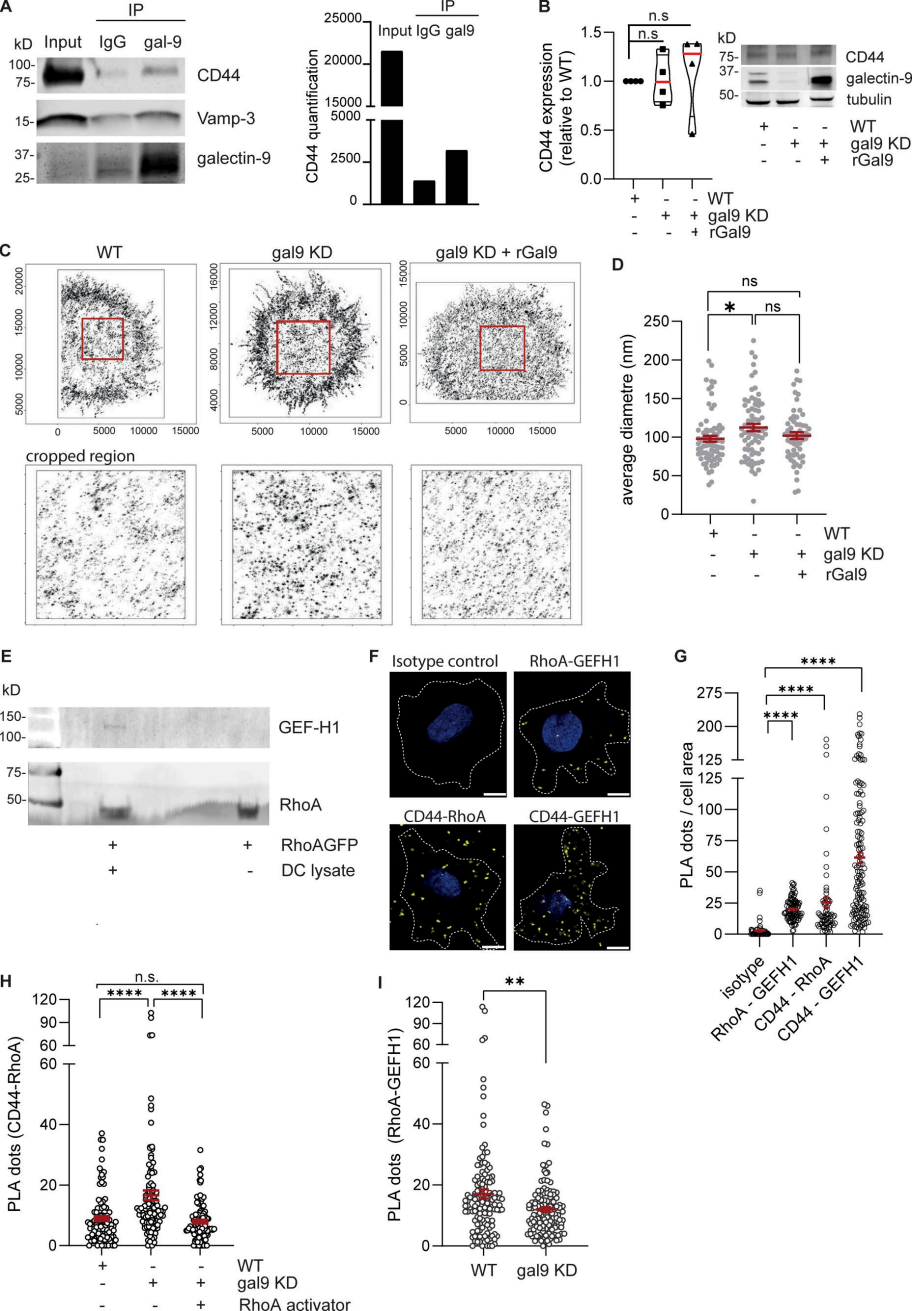
**Galectin-9 interaction with CD44 regulates DC migration. (A)** IP of galectin-9 or isotype negative control in whole-cell lysates obtained from day 7 matured moDCs. Co-IP complexes were resolved and probed against galectin-9, Vamp-3, and CD44. Graph shows quantification of the CD44 signal. Data shown are representative of three independent experiments. **(B)** CD44 expression in WT, gal9 KD, and gal9 KD + rGal9 moDC lysates. Tubulin was used as a loading control. Graph shows mean CD44 expression of four independent donors (black symbols), and immunoblot is representative of four independent experiments. **(C)** Super-resolution dSTORM images of the basal membranes of day 7 matured WT, gal9 KD, and gal9 KD + rGal9 moDCs stained for CD44. ROI (red square) in the upper panel is depicted in the zoomed bottom panels (5,000 × 5,000 nm) to visualize CD44 nanoscale organization. **(D)** Mean ± SEM CD44 cluster diameter in WT, gal9 KD, and gal9 KD + rGal9 was calculated per cell (gray dots) based on pair correlation analysis for 4 independent donors. One-way ANOVA with Tukey’s multiple comparisons was performed. **(E)** RhoA_GFP was incubated with either whole DC lysates from three independent donors pulled together (+) or nothing (−), and immunocomplexes were resolved by western blot and probed with specific antibodies against GEF-H1 and RhoA. Western blot is representative of two independent experiments. **(F)** Confocal microscopy of PLA assessing the proximity of CD44 to RhoA and GEF-H1 in mature moDCs. RhoA-GEF-H1 and isotypes were used as a positive and negative control, respectively. Scale bar: 5 µm. **(G)** Quantification of the number of PLA foci per cell area (black symbols) from images shown in F. Data represent the mean ± SEM from four independent donors (15–20 cells were analyzed/condition). One-way ANOVA with Dunn’s multiple comparison correction was performed. **(H)** Quantification of the number of PLA foci (CD44-RhoA) per cell (black symbols) in WT or gal9 KD moDCs untreated or treated with 5 µg/ml of RhoA activator II. Data represent the mean ± SEM from two independent donors. 25–30 cells were analyzed per condition. One-way ANOVA was performed. **(I)** Quantification of the number of PLA foci (RhoA-GEF-H1) per cell (black symbols) in WT or gal9 KD moDCs. Data represent the mean ± SEM from three independent donors (20 cells were analyzed per condition). An unpaired *t* test was used to assess significance. n.s, P > 0.05; *P < 0.05; **P < 0.01; ****P < 0.0001. IP, immunoprecipitation. Source data are available for this figure: SourceData F7.

The transmembrane adhesion glycoprotein CD44 has been postulated to mediate RhoA activity *via* interactions with RhoGEF through its cytoplasmic tail. We performed co-immunoprecipitation experiments using a galectin-9–specific antibody and identified CD44 to interact with galectin-9 in moDCs (Fig. 7 A). As a positive control, Vamp-3 was also enriched in the galectin-9 IP compared with isotype control as previously reported (Santalla Mendez et al., 2023). Importantly, galectin-9 depletion or supplementation did not affect CD44 expression (Fig. 7 B). Next, we analyzed CD44 nanoscale organization at the surface of WT and gal9 KD DCs using super-resolution direct stochastic optical reconstruction microscopy (dSTORM) (Fig. 7 C). dSTORM images of CD44 clearly show CD44 exists in nanoclusters and in nonclustered form at the surface of DCs. The CD44 cluster size was found to be significantly higher in gal9 KD DCs compared with WT counterparts, and restoring surface galectin-9 levels reverted CD44 cluster size toward that of WT cells (Fig. 7 D). Overall, these data confirm CD44 and galectin-9 interact at the DC surface and suggest galectin-9 aids in modulating CD44 membrane clustering.

CD44 has been linked to RhoA activity before, but the guanine nucleotide exchange factors (GEFs) responsible for specifically activating RhoA in moDCs are not known. To uncover RhoA_GFP binding proteins, we employed GFP-trap pull-down experiments on DC whole-cell lysates followed by mass spectrometry and identified GEF-H1 (or ARHGEF2) to be the most abundant RhoGEF bound to RhoA in DCs. This interaction was confirmed by western blot (Fig. 7 E). GEF-H1 is strongly polarized to trailing-edge regions and is the main GEF driving uropod retraction (Kopf et al., 2020). Most Rho GTPases can be readily localized through staining, but membrane-bound RhoA cannot be distinguished from the cytosolic pool (Michaelson et al., 2001), and thus, to shed light on its interaction with CD44, we turned to proximity ligation assays (PLA) that allow for *in situ* detection of endogenous protein interactions within 40-nm distance. RhoA-GEF-H1 interaction and isotypes were used as positive and negative control, respectively (Fig. 7, F and G). A specific signal indicative of interaction was observed between CD44 and RhoA and between CD44 and GEF-H1 (Fig. 7, F and G), suggesting CD44 forms a complex with RhoA and GEF-H1 in DCs. Based on our time-lapse data, activation of RhoA *via* treatment with RhoA activator was sufficient to rescue the effects of galectin-9 depletion in moDC motility. Therefore, to delineate how the activation status of RhoA determines its interaction with CD44, we analyzed CD44-RhoA binding following gal9 KD in the absence or presence of the RhoA activator (Fig. 7 H). Binding of RhoA to CD44 was enhanced by galectin-9 depletion, which was reduced to WT levels upon treatment with the RhoA activator, indicating CD44 preferentially binds to GDP-bound RhoA. We also employed PLA to further explore RhoA functional interaction with GEF-H1 in response to galectin-9 depletion and demonstrate that RhoA association with GEF-H1 was significantly diminished in gal9 KD DCs, in line with a decreased RhoA activity upon loss of galectin-9 (Fig. 7 I). We conclude galectin-9–organized CD44 binds to and primes inactive RhoA to be activated by GEF-H1 to drive uropod retraction.

### Galectin-9 is sufficient to rescue migration in tumor-immunosuppressed primary DCs

**Figure S5.**
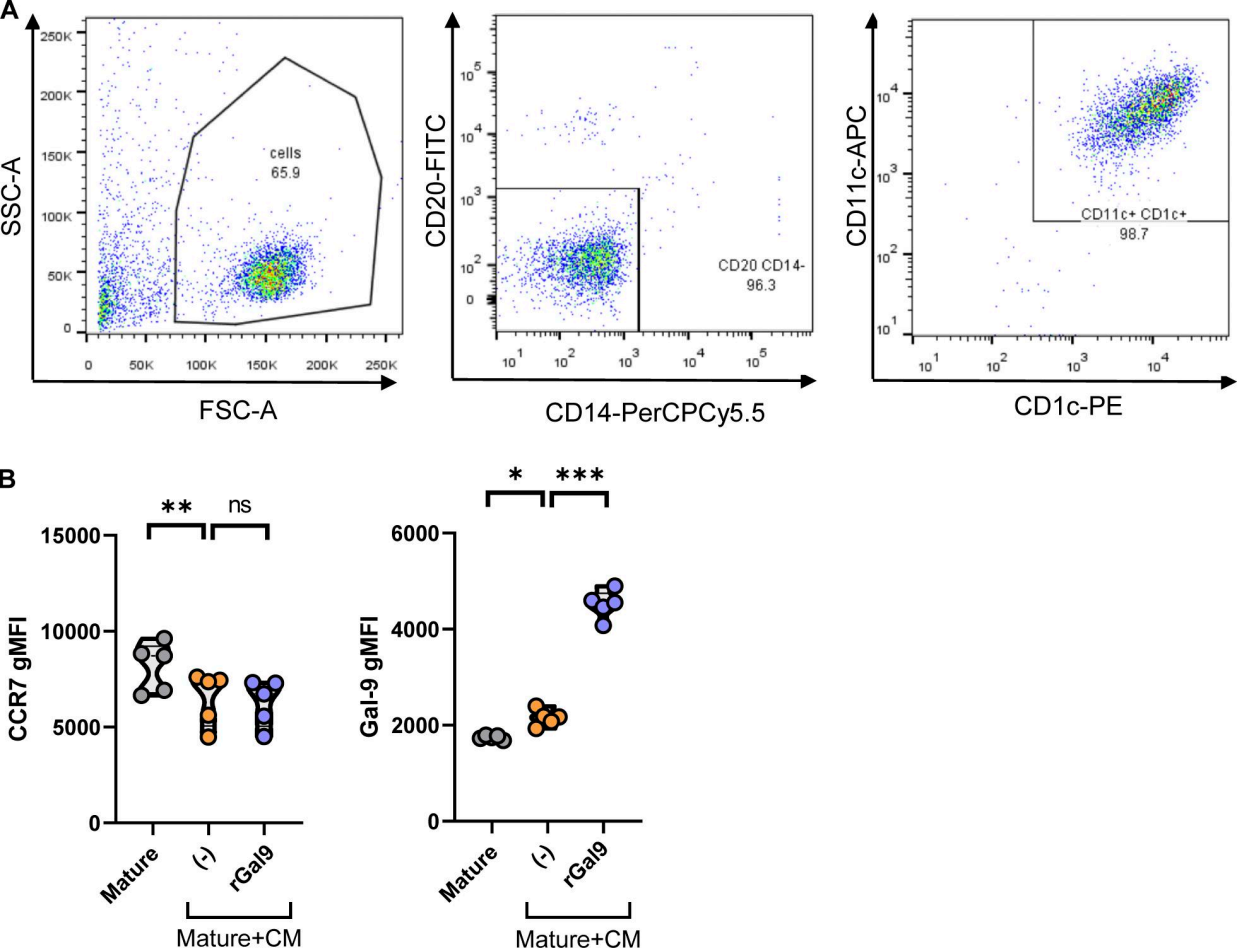
**cDC2 cell purity and supplementary phenotype analysis. (A)** Gating strategy to determine cDC2 purity after isolation from PBMCs. cDC2s were stained for CD1c, CD11c, CD14, and CD20. A first gate is set based on the physical FCS-A/SSC-A parameters followed by selecting on negative cells for CD14 and CD19. cDC2s are then identified as positive cells for CD11c and CD1c. **(B)** Surface galectin-9 and CCR7 expression in galectin-9 and CCR7-positive cDC2s. Graph shows average ± SEM gMFI of five individual donors. One-way ANOVA followed by Dunnett’s test for multiple comparisons was performed. *P < 0.05; **P < 0.01; ***P < 0.001. gMFI, geometric mean fluorescence intensity.

**Figure 8.**
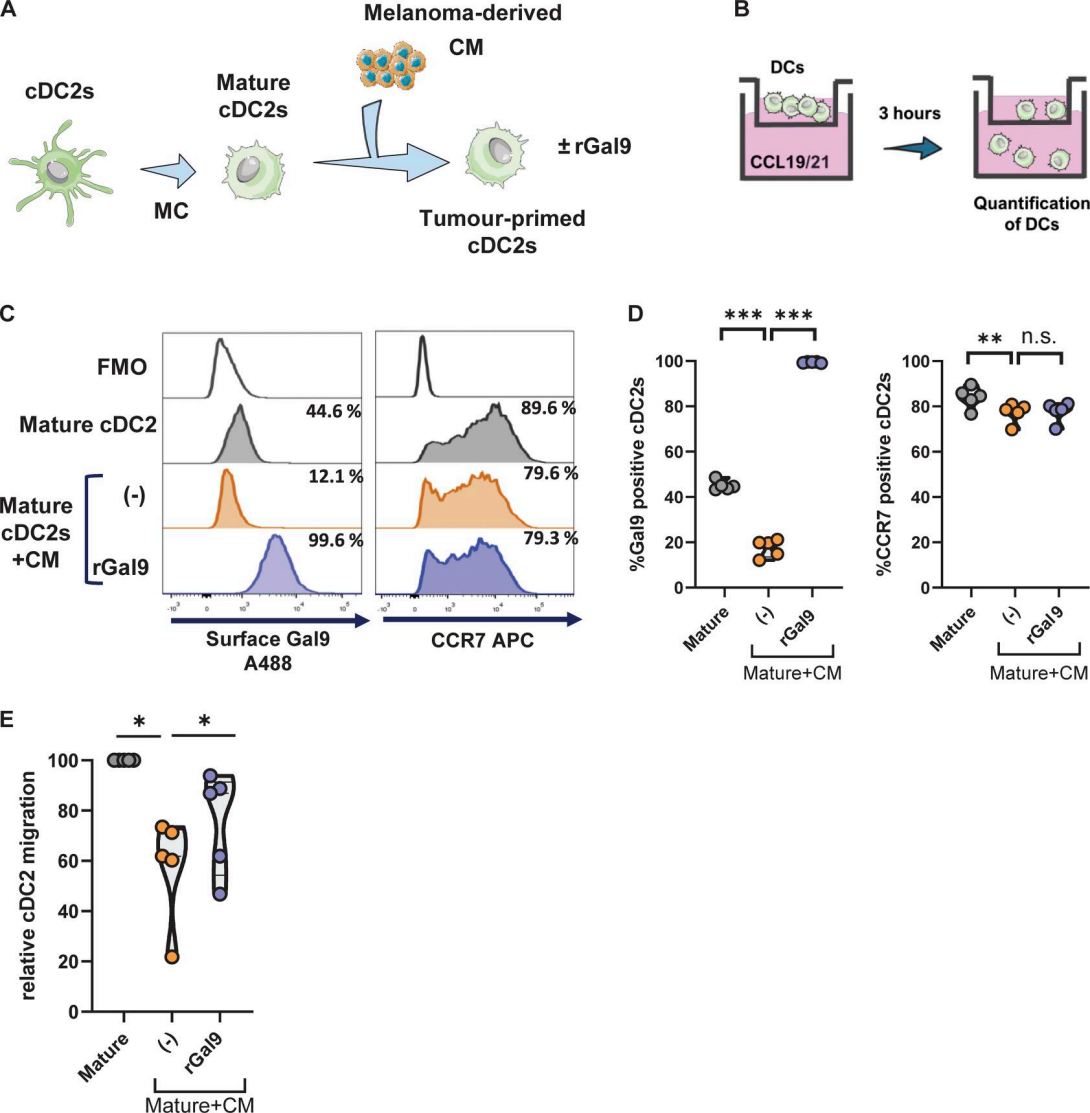
**Galectin-9 restores chemokine-driven cDC2 migration. (A)** Schematic representation of the experimental setup. Primary human cDC2s were matured overnight with a MC for 24 h prior to being harvested and replated in the presence of melanoma-derived CM for 24 h. Exogenous galectin-9 was supplemented for the last 2 h of cDC2 incubation with melanoma-derived CM. Tumor-primed cDC2s were collected and analyzed for the surface expression levels of galectin-9 and CCR7 or for their migratory capacity. **(B)** Schematic representation of the transwell migration assay. cDC2s were seeded in the top chamber of a transwell chamber containing a 5-µm porous membrane and subjected to a chemokine gradient of the CCR7 ligands CCL19 and CCL21. Migratory cDC2s were collected after 3 h and quantified. **(C)** Histograms showing the surface expression of galectin-9 and CCR7 of a representative cDC2 donor analyzed by flow cytometry. **(D)** Percentage of positive cDC2s for galectin-9 and CCR7 for each of the indicated treatments. **(E)** Relative cDC2 migration under each treatment was determined by normalizing each treatment to the migration given by mature cDC2s unexposed to the melanoma-derived CM for every donor. Violin plots in D and E show mean of five independent donors. One-way ANOVA followed by Dunnett’s test for multiple comparisons was performed. *P < 0.05; **P < 0.01; ***P < 0.001. MC, maturation cocktail.

Having defined molecular basis of galectin-9–mediated uropod retraction, we validated the importance of galectin-9 on DC migration using human blood conventional DCs type 2 (cDC2s) as a model to better recapitulate a physiological setup (Fig. S5 A). Mature cDC2s were treated with melanoma-derived conditioned medium (CM) to impair their migratory capacity, after which rGal9 was provided to assess its ability to rescue cDC2 migration (Fig. 8 A). Migratory capacity of DCs was determined in a transwell migration assay toward the chemokines CCL19 and CCL21 for 3 h (Fig. 8.B). Exposure of mature cDC2s to melanoma-derived CM led to the downregulation of surface galectin-9 and CCR7 expression levels (Fig. 8, C and D). In line with the phenotype data, tumor-primed cDC2s exhibited a lower migratory capacity toward the chemokines CCL19 and CCL21 compared with untreated mature cDC2s (Fig. 8 E). Remarkably, the addition of exogenous galectin-9 rescued cDC2 migration toward the chemokines CCL19 and CCL21 (Fig. 8 E). CCR7 expression levels remained unchanged during galectin-9 addition (Fig. 8 D and Fig. S5 B), indicating this superior migratory capacity to be dependent on galectin-9 presence and not on an altered CCR7 expression. Taken together, these results validate the relevance of galectin-9 for the migration capacity of naturally occurring DC subsets in a tumor model and illustrate the therapeutic value of intervening galectin-9 signaling axis to restore the migration of tumor-immunocompromised DCs.

## Discussion

Single-cell migration is a ubiquitous phenomenon in mammalian cell biology with cells mostly displaying either a mesenchymal or an amoeboid migration mode. The latter is employed by DCs, allowing for a fast and autonomous migration, driven by actin polymerization and actomyosin contractility forces. This is essential to rapidly shuttle antigens from peripheral tissues to lymphoid organs. However, how environmental cues and cell membrane organization integrate and modulate cytoskeleton remodeling and contractility remains poorly characterized.

**Figure 9.**
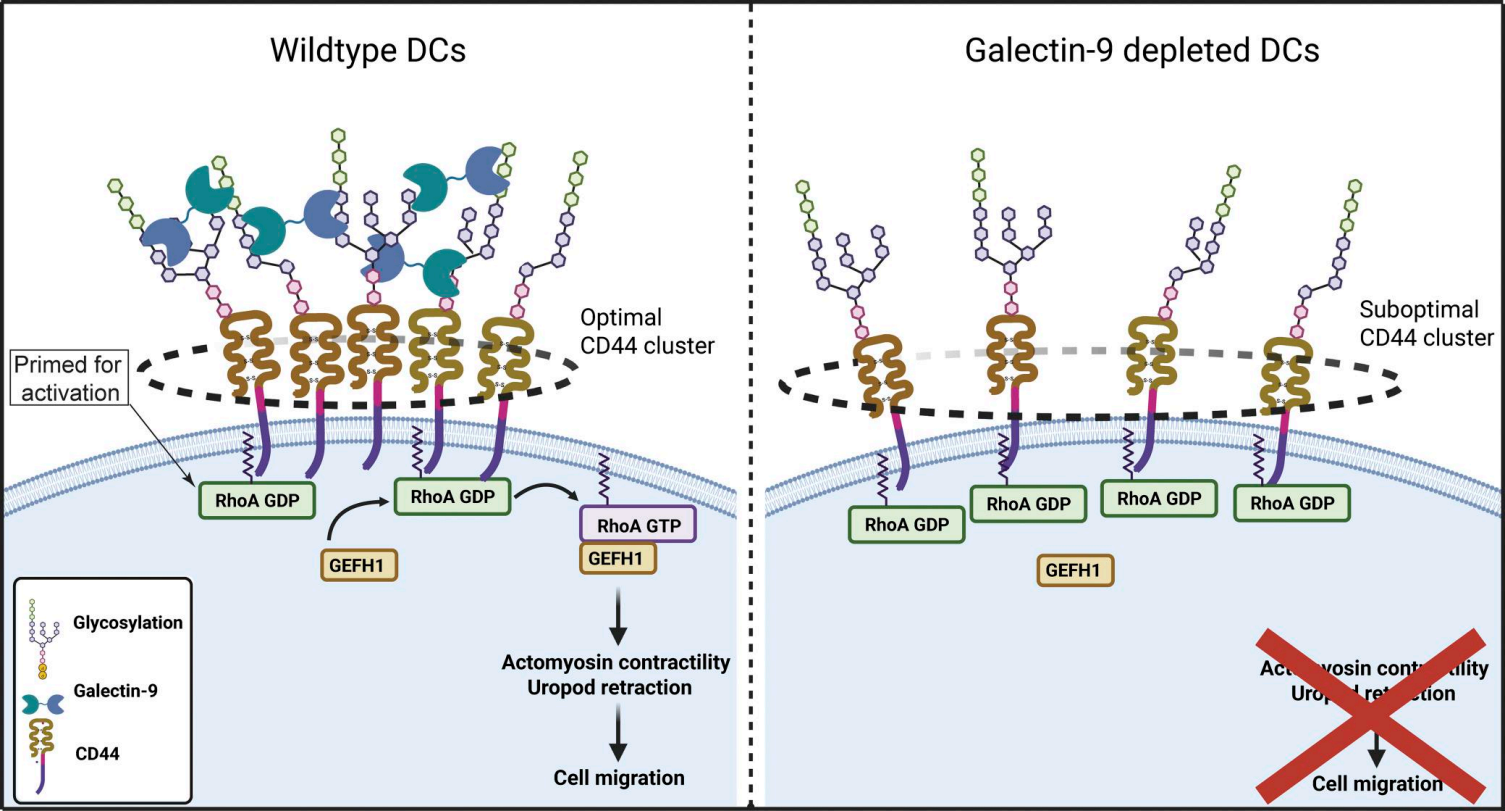
**Graphical abstract. Galectin-9 controls cell rear contractility.** Left: Galectin-9 binds to glycosylated residues on CD44 inducing CD44 clustering. This facilitates a functional interaction between CD44 and RhoA-GDP, priming RhoA for activation by GEF-H1. RhoA activation leads to actin contractility at the cell rear and migration. In the absence of galectin-9 (right panel), CD44 clustering is deficient, which results in defective RhoA activation and impaired uropod retraction and migration. Created in BioRender. Franken, G. (2025) https://BioRender.com/9zh1jyh.

In this study, we uncovered a role of galectin-9 in DC migration and report a novel function in controlling RhoA-mediated rear actomyosin contractility *via* modulating CD44 membrane organization and RhoA association. Through the use of 3D cultures and *in vivo* techniques, we have dissected the role of galectin-9 in actomyosin contractility in physiologically relevant settings (although we found galectin-9 function to be partially conserved in 2D migration). We demonstrate that galectin-9 regulates basal and chemokine-driven DC motility in humans and mice, suggesting a conserved function for this lectin. We propose that CD44 preferentially associates with inactive RhoA (GDP-bound) at the intracellular side of the plasma membrane. Upon activation, GEF-H1 dissociates from microtubules (Kopf et al., 2020; Renkawitz et al., 2019), thereby triggering RhoA disengagement from CD44 and the initiation of downstream signaling cascades that result in uropod contractility and cell movement (Fig. 9). We also reveal that immunosuppressed blood cDC2 cells express low levels of galectin-9 resulting in an impaired motility that can be rescued upon restoring galectin-9 levels. These data validate our results obtained using *in vitro* and *in vivo* models and highlight the importance of galectin-9 in cell migration in naturally occurring human blood DCs.

Galectin-9 acts as an immune suppressor in B and T cells (Cao et al., 2018; Zhu et al., 2005), while we and others have reported a stimulatory function for galectin-9 in DCs. For instance, the addition of rGal9 induced DC maturation (Dai et al., 2005) and we have demonstrated galectin-9 is necessary for optimal phagocytic capacity and cytokine secretion in DCs (Querol Cano et al., 2019; Santalla Mendez et al., 2023). Interestingly, those functions are mediated by the intracellular fraction of galectin-9, whereas data presented here indicate the surface-bound pool of galectin-9 is responsible for its role in cell migration. Earlier reports have shown galectin-9 promotes T_H_2 migration *via* modulation of integrin CD61 activity (Bi et al., 2011). Similarly, galectin-9 enhanced neutrophil adhesion *via* the regulation of integrin (CD11b and CD18) activity at the cell membrane (Iqbal et al., 2022). This is in contrast to data illustrating a role of intracellular galectin-3 in DC migration (Hsu et al., 2009; Kataoka et al., 2019), which highlights how different galectins exert distinct functions and that their mechanisms of action might be cell-specific. Many surface adhesion receptors are glycosylated, and it cannot be excluded that galectin-9 depletion alters their membrane dynamics, causing an abnormal overadhesive phenotype that in turn results in an elongated uropod. In addition, our data do not exclude that intracellular galectin-9, which remains largely depleted after treatment with the exogenous protein, is also involved in DC migration (Querol Cano et al., 2019). Endogenous galectin-9 is highly susceptible to being cleaved in solution, and therefore, the used commercial rGal9 has a shortened linker for additional stability, which may affect its binding or cross-linking abilities. Overall, data presented here indicate that the formation of galectin-9–mediated functional CD44 domains at the DC membrane may act as a scaffold to functionally cluster RhoA and GEF-H1, enabling proper downstream RhoA signaling crucial for leukocyte propulsion.

CD44 is located to uropods in migrating cells and can directly modulate RhoA activity *via* its cytosolic tail (Bourguignon, 2008; Bourguignon et al., 2010; Zhang et al., 2014). Here, we demonstrate that galectin-9 interacts with CD44 in DCs in line with data in neutrophils, and natural killer and T cells (Iqbal et al., 2022; Rahmati et al., 2023). CD44 is highly glycosylated, and galectin-9 binding directly induced CD44 downstream signaling in natural killer cells and neutrophils, but mechanistic insights are largely lacking (Dunsmore et al., 2021; Rahmati et al., 2023). CD44 exhibits a nonrandom membrane distribution independent of its binding to ligand, pointing toward an intrinsic organization of the protein (Sil et al., 2020). Our super-resolution dSTORM data revealed enlarged CD44 clusters upon loss of galectin-9, which was partially rescued with rGal9. Given that membrane receptor clustering involves a complex interplay of molecular pathways (van Deventer et al., 2021), the effects observed on CD44 clustering are likely multifactorial. For instance, galectin-9 loss might be permissive to other clustering proteins such as flotillins or tetraspanins to organize CD44 into high-order structures. On the other hand, galectin-9 might anchor CD44 in specific nanodomains, and thus, galectin-9 depletion may lead to random encountering of CD44 molecules, similar to DC-SIGN upon loss of its N-glycosylation site (Torreno-Pina et al., 2014). This is in line with reports indicating that interactions mediated by CD44 extracellular domain ensure lower receptor diffusion (Sil et al., 2020). In addition, actomyosin perturbations caused by galectin-9 loss may also affect CD44 clustering through its intracellular interactions with actin, thereby contributing to its altered cluster pattern in galectin-9 KD DCs. Although further experimental investigation is warranted to delineate the precise structural organization and dynamics of CD44–galectin-9 complexes, our data show that enlarged CD44 clustering is accompanied by decreased RhoA activity suggesting an optimal CD44 cluster size is required to effectively induce downstream signaling.

We demonstrate that the active (GTP-bound) fraction of RhoA diminishes in gal9 KD DCs, providing the molecular basis for the uropod retraction defect as RhoA-mediated cytoskeletal reorganization is indispensable for DC migration. The spatiotemporal activation of Rho GTPase signaling is dynamically regulated by many GEFs and GAPs, which in turn form complexes with a variety of other proteins (Lawson and Ridley, 2018). We identified GEF-H1 as the main RhoA-GEF in DCs, and our phosphoproteomics and STRING analysis on naïve or LPS-stimulated moDCs revealed that GEF-H1 is connected to PAK1 in moDCs to regulate cell adhesion (Warner et al., 2024). Further supporting our findings, GEF-H1 is the main GEF driving actomyosin retraction in amoeboid migration (Kopf et al., 2020; Renkawitz et al., 2019). GEF-H1 release is induced upon microtubule destabilization in DCs and is required for DC maturation and activation (Kashyap et al., 2019; Krendel et al., 2002). The expression of surface DC maturation markers was not affected by galectin-9 loss, suggesting GEF-H1–associated transcriptomic changes are independent of galectin-9. Nonetheless, it would be interesting to examine the crosstalk between GEF-H1 and microtubule formation in galectin-9 KD DCs to determine how RhoA activation status contributes to this process. Furthermore, nuclear repositioning in 3D amoeboid migration is regulated through GEF-H1–driven actomyosin contractility and myosin polarization to the rear of the nucleus (Kroll et al., 2023). Loss of myosin function resulted in a random nucleus-to-microtubule-organizing center axis configuration, reminiscent of the lack of nucleus repositioning to the cellular rear upon galectin-9 loss.

RhoA signaling is known to be dynamically activated at both the leading edge and uropod of migrating cells (Pertz et al., 2006). In our study, galectin-9 depletion selectively impaired uropod contraction, while migration at the cell front was largely unaffected. This suggests other small GTPases, such as Rac1 or cdc42, may (partially) compensate for reduced RhoA activity at the cell front (Machacek et al., 2009), whereas no such compensatory mechanism exists at the rear. Furthermore, we identified a functional association between RhoA and GEF-H1, which selectively regulates uropod contractility without influencing the leading edge (Heasman et al., 2010), underscoring the spatial specificity of RhoA-mediated signaling during cell migration. The most well-studied signaling pathway downstream of RhoA is that mediated by ROCK, leading to pMLC and increased actomyosin contractility. RhoA can also directly bind and activate mDia to enhance actin polymerization (Clayton and Ridley, 2020; Vargas et al., 2016). No changes in pMLC protein levels were detected in 2D-seeded cells, possibly related to the stiffness of the 2D culture (Yang et al., 2017; Lee and Kumar., 2016), but its subcellular 3D localization appears to be modulated in response to galectin-9 expression. pMLC failure to accumulate at the rear of 3D migrating gal9 KD DCs underlies the impairment in actomyosin contractility and abnormal actin distribution observed upon galectin-9 depletion. Furthermore, the activity of other RhoA downstream targets like PAK1 was found to correlate with galectin-9 levels, suggesting a specific regulation of the PAK pathway by galectin-9–dependent interactions. PAK1 is involved in cytoskeletal remodeling and is activated by phosphorylation at Thr423 mediated by Rac1, Cdc42, and RhoA (Mayhew et al., 2007; Szabo et al., 2022; Zhang et al., 2018). Decreased phosphorylation at this site indicates a lack of RhoA activity, and in general a decreased polarized state of gal9 KD cells. Interestingly, our analysis of publicly available datasets confirmed a positive correlation between RhoA transcript levels and galectin-9 treatment, suggesting that galectin-9 control over RhoA signaling is conserved across immune cell types (Wang et al., 2016).

DC trafficking from the tumor site to lymph node structures is crucial for the effective induction of antitumor responses (Liu et al., 2021). However, the suppressive tumor microenvironment is known to foster DC dysfunction, impeding DC motility and subsequent launching of adaptive immunity, thereby enabling tumor progression (Imai et al., 2012; Villablanca et al., 2010). How galectin-9 shapes the immune compartment within the tumor microenvironment has scarcely been addressed despite its relevant role driving tumorigenic processes (Yang et al., 2021; Zhang et al., 2020). Here, we demonstrate that exposure of cDC2s to tumor CM induced galectin-9 downregulation and impaired cell migration. Treatment with rGal9 was sufficient to restore chemokine-driven migration in immunosuppressed blood DCs, which underlies the general relevance of galectin-9 in the context of multiple immunological settings.

Taken together, our study demonstrates the importance of galectin-9 in DC basal and directed migration toward lymph nodes and tumors. Furthermore, we provide for the first time evidence that galectin-9 regulates cell polarity and uropod contraction, namely by modulating RhoA activation in response to CD44 binding. Lastly, data presented here highlight a role of galectin-9 in promoting DC motility in the tumor microenvironment, underscoring galectin-9 as a target in DC-mediated antitumor immunity.

## Materials and methods

### Generation of moDCs

DCs were derived from peripheral blood monocytes isolated from a buffy coat (Sanquin, Nijmegen, The Netherlands) (de Vries et al., 2002). Blood samples were obtained from donors irrespective of age and gender, as these variables were not expected to influence the outcomes of the study. Monocytes isolated from healthy blood donors (informed consent obtained) were cultured for up to 8 days in RPMI 1640 medium (Life Technologies, Bleiswijk, The Netherlands) containing 10% fetal bovine serum (FBS, Greiner Bio-One, Alphen aan den Rijn, The Netherlands), 1 mM ultraglutamine (BioWhittaker), antibiotics (100 U/ml penicillin, 100 µg/ml streptomycin, and 0.25 µg/ml amphotericin B; Life Technologies), IL-4 (500 U/ml; Miltenyi Biotec), and granulocyte macrophage–colony-stimulating factor (GM-CSF; 800 U/ml, #130-093-868; Miltenyi Biotec) in a humidified, 5% CO_2_ incubator. On day 3, medium was refreshed with new IL-4 (500 U/ml; Miltenyi Biotec) and GM-CSF (800 U/ml; Miltenyi Biotec). On day 6, moDCs were supplemented with a maturation cocktail: IL-6 (15 ng/ml, #130-093-933; Miltenyi Biotec), TNF-α (10 ng/mg, #130-094-014; Miltenyi Biotec), IL-1β (5 ng/ml, #130-093-898; Miltenyi Biotec), and PGE2 (10 µg/ml; Pfizer). When necessary, moDCs were treated with recombinant galectin-9 protein (AF2045; R&D Systems) at a final concentration of 1 µg/ml.

### Isolation and culture of primary cells

Human cDC2s were isolated from peripheral blood mononuclear cells derived from healthy individuals (Sanquin, Nijmegen, The Netherlands) using the MACS CD1c^+^ isolation kit (130-119-475; Miltenyi Biotec) according to the manufacturer’s instructions. Cell purity was determined by flow cytometry (Fig. S5 A) using antibodies specific against CD20-FITC (1:100 345792; BD Biosciences), CD14-PerCP (1:50, 325632; BioLegend), CD11c-APC (1:50 559877; BD Biosciences), and CD1c-PE (1:50, #130-113-302; Miltenyi Biotec). After isolation, fresh cDC2s were cultured in X-VIVO-15 (Lonza) supplemented with 2% human serum (HS, Sigma-Aldrich) at a concentration of 0.5 × 10^6^ cells/ml. cDC2s were matured overnight with a maturation cocktail composed of 50 U/ml GM-CSF, 100 U/ml IL-6, 100 U/ml IL-1β, 50 U/ml TNFα, and 200 nM of PGE2. The next day, cDC2s were harvested, washed, and replated in media composed of 50% fresh X-VIVO-15 supplemented with 2% HS and 50% melanoma cell line A375–derived CM for 24 h. When necessary, 1 µg/ml of recombinant galectin-9 was provided for the final 2 h of cDC2 coculture with melanoma-derived CM. Next, cDC2s were harvested and washed prior to further analysis.

For BMDCs, bone marrow was taken from the tibias and femurs of 9-wk-old C57BL/6 *Lgals9*^−/−^ mice or WT litter mates and cultured in RPMI containing 10% FBS and 3% murine GM-CSF (PeproTech) for 7 days. Cells were treated with 1 µg/ml LPS for 16 h prior to being used. All murine studies complied with European legislation (Directive 2010/63/EU of the European Commission) and were approved by local authorities (CCD, The Hague, The Netherlands) for the care and use of animals with related codes of practice. Power calculations were performed to determine the minimum number of animals required per group to detect statistically significant differences, ensuring adequate power while minimizing animal use.

### Generation of tumor spheroids and CM preparation

The melanoma cell lines MEL624 (ATCC)  (RRID:CVCL_8054) and BLM (AIMM Therapeutics) (RRID:CVCL_7035) were cultured in Gibco DMEM high-glucose medium (Life Technologies) supplemented with 10% FBS and 0.5% antibiotics (100 U/ml penicillin, 100 µg/ml streptomycin, and 0.25 µg/ml amphotericin B; Life Technologies). A375 melanoma cell line (ATCC)  (RRID:CVCL_0132) was cultured in DMEM (high glucose, GlutaMAX; Gibco) and further supplemented with 10% FBS (HyClone) and 1% antibiotic–antimycotic (Gibco). To generate tumor spheroids, cells were harvested using PBS containing 0.25% trypsin and 4 mM EDTA and collected in Gibco DMEM high-glucose medium (Life Technologies) containing 10% FBS. For spheroid production, tumor cells were cultured in 30 µl droplets containing 4,000 tumor cells resuspended in spheroid medium (60% growth medium and 40% low-viscosity methyl cellulose medium (25cp viscosity); Sigma-Aldrich Life Science), to which 3.6 µl of PureCol Type I Collagen (3.1 mg/ml stock solution; Advanced BioMatrix) was added.

To obtain melanoma-derived CM, A375 cells were seeded at a cell concentration of 0.25 × 10^6^ cells/ml. After 72 h, A375 CM was harvested and centrifuged at 400 *g* for 5 min to get rid of cellular debris and frozen until further use.

Cell lines were regularly authenticated by STR profiling.

### Small interfering RNA knockdown

On day 3 of DC differentiation, cells were harvested and subjected to electroporation. Three custom stealth small interfering RNAs (siRNAs) were used to silence galectin-9 (LGALS9HSS142807, LGALS9HSS142808, and LGALS9HSS142809) (Invitrogen). Equal amounts of the siRNA ON-TARGETplus NT siRNA#1 (Thermo Fisher Scientific) were used as a control. Cells were washed twice in PBS and once in Opti-MEM without phenol red (Invitrogen). A total of 15 μg siRNA (5 μg from each siRNA) was transferred to a 4-mm cuvette (Bio-Rad), and 5–10 × 10^6^ DCs were added in 200 μl Opti-MEM and incubated for 3 min before being pulsed with an exponential decay pulse at 300 V, 150 mF, in a Gene Pulser Xcell (Bio-Rad), as previously described (Querol Cano et al., 2019; Santalla Mendez et al., 2023). Immediately after electroporation, cells were transferred to preheated (37°C) phenol red–free RPMI 1640 culture medium supplemented with 1% ultraglutamine, 10% (vol/vol) FCS, IL-4 (300 U/ml), and GM-CSF (450 U/ml) and seeded at a final density of 5 × 10^5^ cells/ml.

### Chemotaxis assays

Day 6 mature NT or *LGALS9* siRNA-transfected moDCs (1 × 10^5^ cells in 50 µl) were seeded in the top chamber of a 24-well transwell containing a polycarbonate filter of 5 µm pore size (#CLS3421; Corning). 550 µl of RPMI medium supplemented with 1 µg/ml recombinant human CCL21 (#582208; BioLegend) or nothing as a negative control was added to the lower chambers. Plates were incubated for the specified time points at 37°C, 5% CO_2_ after which migrated cells in the bottom chamber were collected and acquired on MACSQuant Analyzer 10 Flow Cytometer using MACSQuantify software (Miltenyi Biotec). The percentage of specific migration was calculated by dividing the number of cells migrated to the lower well by the total cell input (50-µl cell suspension directly measured on MACSQuant Flow Cytometer). When relevant, cells were stained with 1 µl CellTrace Far Red (Invitrogen) for 30 min at 37°C prior to being seeded in the top chamber of a transwell. After 24-h incubation, collagen gels were fixed with 4% PFA for 20 min at RT and imaged using Zeiss Laser Scanning Microscope 900, equipped with a 10× objective (Zeiss). Z-stacks were made at 5.4-µm intervals.

Chemotactic assays using cDC2s were performed using the transwell 96-well plate 5 µm pore size (#CLS3388-2EA; Corning). 3 × 10^4^ cDC2s were seeded in the top chamber of the transwell. The lower compartment of the transwell plate was loaded with 200 µl of X-VIVO-15 supplemented with 2 % HS and 100 ng/ml of CCL19 and CCL21 (#582102, #582202; BioLegend). To determine passive cDC2 migration, media without CCL19 or CCL21 were used. The plate was incubated for 3 h at 37°C, 5% CO_2_ after which migrated cells in the bottom chamber were collected and acquired on MACSQuant Analyzer 10 Flow Cytometer together with the initially loaded cDC2s for each condition. The percentage of cDC2 migration was assessed by dividing the number of migrated cDC2s by the number of initially loaded cDC2s for each condition. Relative cDC2 migration was determined by dividing the percentage of migrated cDC2s of each condition by the percentage of migrated mature cDC2s unexposed to melanoma-derived CM × 100.

### 3D migration assays

A collagen mixture was generated using *PureCol* Type I Bovine Collagen Solution (Advanced BioMatrix, final concentration 1.7 mg/ml), α-modified minimal essential medium (Sigma-Aldrich), and sodium bicarbonate. Collagen mixture was allowed to prepolymerize for 5 min at 37°C prior to adding a 45-µl cell suspension containing 30,000 mature day 8 NT or *LGALS9* siRNA-transfected moDCs in phenol red–free RPMI 1640 culture medium supplemented with 1% ultraglutamine and 10% FBS. The total mixture (100 µl/well) was transferred to a 96-well black plate (#655090; Greiner Bio-One) and incubated for 45 min at 37°C to allow collagen polymerization. Afterward, 100 µl of phenol red–free RPMI 1640 culture medium supplemented with 1% ultraglutamine and 10% FBS was added on top of the matrices. When appropriate, WT or galectin-9–depleted moDCs were pretreated with 1 µg/ml recombinant galectin-9 for 3, 16, 24, or 48 h prior to being embedded into the collagen gel. When applicable, 5 µg/ml of the RhoA activator II (#CD03; Cytoskeleton) was added to the collagen gel after polymerization. When relevant, moDC suspension was mixed with one tumor spheroid prior to being added to the collagen matrix and the microscopy plate was inverted after every 7–10 min during collagen polymerization to prevent the spheroid from sinking into the matrix. In collagen gels containing tumor spheroids, moDCs were stained with PKH-26 (Sigma-Aldrich) or CellTrace CFSE or CellTrace Far Red (Invitrogen) according to the manufacturer’s instructions and to distinguish them from tumor cells. After 1 day of incubation with spheroids, collagen gels were first fixed with 2% PFA in PBS for 5 min, then 4% PFA in PBS for 20 min, both at 37°C. To localize the spheroid, collagen gels were subsequently incubated with Alexa Fluor 488 phalloidin (A12379; Invitrogen) or phalloidin-iFluor 647 (ab176759; Abcam) in PBS + 3% bovine serum albumin (BSA), 0.1 M glycine, and 0.3% Triton for 3 h at room temperature. Imaging was performed with a Zeiss LSM880 confocal microscope, using a 10× 0.45 NA air objective (Zeiss) to make z-stacks with 5-μm intervals. To quantify spheroid infiltration and the number of moDCs surrounding the spheroid, one plane was taken about 50 μm into the spheroid and moDC count was performed by first setting a threshold and then by the Analyze Particles feature in Fiji (ImageJ). For moDCs surrounding the spheroid, a circular band with a fixed surface area surrounding the spheroid was taken for analysis.

Time-lapse video microscopy was performed using the BD Pathway 855 spinning disk confocal microscope (BD Bioscience), atto vision software (BD Bioscience), and the Plan-Neofluar 10 × 0.3 NA air objective (Olympus). Sequential images were acquired every 4–5 min for 10 h using the 548/20 excitation filter (Chroma), emission filter 84101 (Chroma), and dichroic filter 84000 (Chroma). When appropriate, time-lapse microscopy was performed using the Celldiscoverer 7 (Zeiss), using the 5 × 0.35 NA objective with 2× tube lens or the BD Pathway 855 spinning disk confocal microscope (BD Bioscience), the atto vision software (BD Bioscience), and the Plan-Neofluar 10 × 0.3 NA air objective (Olympus). Time-lapse sequences were analyzed with the ImageJ manual tracking plugin to measure cell velocity and to track individual cells.

### Chemotactic glass chambers

Migration chamber was prepared as previously described (Friedl and Brocker, 2004; Wolf et al., 2013). Briefly, 50,000 day 8 NT or *LGALS9* siRNA-transfected DCs were mixed into collagen gels as before. The cell-containing collagen solution was added to the migration chamber until this was 2/3 filled. The migration chamber was then placed in an upright position at 37°C for 30 min after which the chamber was filled with prewarmed medium containing 1 µg/ml recombinant human CCL21 or nothing as a negative control. The migration chamber was then sealed with an additional lane of wax. Time-lapse microscopy was performed using a live-cell microscopy setup (Okolab) with a Plan-Neofluar 10 × 0.3 NA air objective.

### Immunofluorescence

Cells embedded into collagen gels were fixed with 4% PFA for 15 min at 37°C, washed with PBS, permeabilized with 0.2% (vol/vol) Triton in PBS for 15 min, followed by another wash with PBS, and blocked for 1 h in 3% BSA (wt/vol) in PBS. Gels were then incubated with 1:40 phalloidin-A488 (#A12379; Sigma-Aldrich) for 16 h, washed three times with PBS followed by Hoechst (1:200, #33342), and washed twice with PBS. Gels were then incubated with 1:50 pMLC2 (Thr18/Ser19) (#3674; Cell Signaling Technology) for 24 h, washed three times with PBS, labeled with anti-rabbit Cy3 (#111-165-144; Jackson ImmunoResearch) for 16 h, and washed three times prior to imaging.

Immunofluorescence imaging was performed on a Nikon Ti2 spinning disk confocal microscope with Crest Optics SIM module for super-resolution imaging. Cells were imaged using a fully motorized Nikon Ti2 inverted microscope stand, fitted with a Prior Scientific NanoScan SP600 piezo insert. The microscope stand is equipped with Crest Optics X-light V3 spinning disk (50 µm pinhole size) confocal unit and Crest Optics DeepSIM X-Light with Standard Mask (37 images). Images were sampled by spinning disk confocal (for analysis) or SIM super-resolution modalities (for Video 4). Images were captured using a Teledyne Photometrics Kinetix camera through a 60×/1.42 DIC Plan Apo oil-immersion objective (for quantified images), or a 100×/1.45 DIC Plan Apo oil-immersion objective for super-resolution 3D stacks (Video 4) with Ibidi Immersion Oil 2. Specific fluorescence was collected by illuminating the sample using a 89North LDI multiwavelength laser diode, with discrete wavelength channels; 405, 470, and 555 were used in combination with a Penta Band Exciter, 1CRPENT1. The emission light was passed through a Penta polychroic filter, 1CRPENT3, and further narrowed down with a (DAPI 440/40, FITC 510/50, or Cy5 655LP) filter from Chroma using a filter wheel in the light path. The NIS-Elements AR 5.42.06 acquisition software was used to acquire the data, which were saved in the ND2 file format.

### *In vivo* adoptive transfer

WT and galectin-9^−/−^ DCs were labeled with 5 µM CFSE violet and far-red dyes following the manufacturer’s instructions (#C34571 and C34572; Invitrogen, respectively), mixed in equal numbers (1 × 10^6^ each in 50 µl), and co-injected into the same footpad or tail vein of either WT or galectin-9^−/−^ recipient mice. Donor DCs arriving in the draining lymph node (popliteal and inguinal, respectively) were enumerated 48 h later by flow cytometry using BD FACSLyric Flow Cytometer (BD BioSciences). The homing index was calculated using the following formula: (% far-red signal in tissue/% violet signal in tissue)/(% far-red signal in input/% violet signal in input).

### Reverse-phase protein array

Cellular proteins were denatured in a 1% SDS + 2-mercaptoethanol buffer solution and diluted in five twofold serial dilutions in dilution lysis buffer. Serially diluted lysates were arrayed on nitrocellulose-coated slides (Grace Bio-Labs) by Quanterix (Aushon) 2470 Arrayer (Quanterix Corporation), and each slide was probed with a validated primary antibody plus a biotin-conjugated secondary antibody (https://www.mdanderson.org/research/research-resources/core-facilities/functional-proteomics-rppacore/antibody-information-and-protocols.html). Signal detection was amplified using an Agilent GenPoint staining platform (Agilent Technologies) and visualized by DAB colorimetric reaction. The slides were scanned (Huron TissueScope, Huron Digital Pathology) and quantified using customized software (Array-Pro Analyzer, Media Cybernetics) to generate spot intensity. The relative protein level for each sample was determined by RPPA SPACE (Shehwana et al., 2022) (developed by MD Anderson Department of Bioinformatics and Computational Biology, https://bioinformatics.mdanderson.org/public-software/rppaspace/). The protein concentrations of each set of slides were then normalized for protein loading. The correction factor was calculated by (1) median centering across samples of all antibody experiments; and (2) median centering across antibodies for each sample. Results were then normalized across RPPA sets by replicate-based normalization as described previously (Akbani et al., 2014). Details of the RPPA platform as performed by the RPPA Core are described in Siwak et al. (2019). Pathway enrichment analysis was performed using the Metascape platform and the analysis outlined in Zhou et al. (2019).

### Flow cytometry

To determine depletion of galectin-9 following siRNA transfection, single-cell suspensions were stained with a goat anti-galectin-9 antibody (AF2045; R&D Systems) at 8 μg/ml or isotype control as a negative control for 30 min at 4°C. Before staining, moDCs were incubated with 2% human serum for 10 min on ice to block nonspecific interaction of the antibodies with Fc receptors. A donkey anti-goat secondary antibody conjugated to Alexa Fluor 488 was used (#A-11055, 1:400; Invitrogen [vol/vol]).

moDCs were incubated for 30 min on ice with antibodies against HLA-DR (#555811, clone G46-6, FITC-labeled; BD BioSciences), CD80 (#557227, clone C3H, PE-labeled; BD BioSciences), CD86 (#555658, clone 2331, PE-labeled; BD BioSciences), CD83 (#130-094-186, clone HB15, APC-labeled; Miltenyi), and CCR7 (#130-094-286; Miltenyi Biotec). All antibodies were used at a final 1:25 (vol/vol) dilution in cold PBS containing 0.1% BSA, 0.01% NaH_3_ (PBA) supplemented with 2% HS.

To phenotype cDC2s, harvested cDC2s were first blocked in PBA buffer supplemented with 2% HS for 15 min. After blocking, cDC2s were stained in PBA buffer with anti-galectin-9 antibody (1:50, AF2045; R&D Systems) and anti-CCR7 (1:200) for 20 min. Next, cells were washed and stained with a secondary antibody, a donkey anti-goat antibody conjugated to Alexa 488 (1:400; Invitrogen) for 20 min. Stained cDC2s were analyzed by flow cytometry using a FACS Verse (BD) and later analyzed using FlowJo software (BD).

### Proximity ligation assays

250,000 NT or *LGALS9* siRNA moDCs were seeded onto coverslips and after overnight LPS treatment (1 µg/ml) fixed with 4% PFA at room temperature for 20 min. When applicable, 5 µg/ml of the RhoA activator II (#CD03; Cytoskeleton) was added 3 h before fixation. Afterward, PLA were performed using Duolink *In Situ* PLA Orange kit (Sigma-Aldrich) according to the manufacturer’s instructions. Cells were blocked with Duolink blocking solution with 2% human serum, for 60 min at 37°C. Then, cells were incubated with Duolink Antibody Diluent with 2% human serum supplemented with primary antibodies against RhoA (1:300, #sc-418; Santa Cruz and 1:200, #2117S; Cell Signaling), GEF-H1 (1:600, #GTX125893; GeneTex), and CD44 (1:500, #8E2F3; Novus Biologicals) for 30 min at 37°C. After washing with Wash Buffer A, cells were stained with PLUS and MINUS PLA probes diluted 1:5 in Duolink Antibody Diluent with 2% human serum for 1 h at 37°C. Cells were washed again with Wash Buffer A before and after ligation was performed in 1x Duolink ligation buffer for 30 min at 37°C. Next, rolling circle amplification was performed using 1x Orange amplification buffer for 100 min at 37°C. After washing with Wash Buffer B, samples were stained with DAPI, washed, and embedded in Mowiol. Acquisition was done using Zeiss LSM880 using a 63 × 1.4 NA oil-immersion objective. Image analysis and PLA spot quantification were performed in Fiji.

### RhoA pull-down

2–3 × 10^6^ day 6 WT, galectin-9 KD, or galectin-9 rDCs were collected. RhoA GTPase activity was measured using RhoA Pull-Down Activation Assay Biochem Kit (#BK036; Cytoskeleton) according to the manufacturer’s instructions. Active RhoA protein was quantified using the Image Studio Lite software (LI-COR).

### Western blot

Day 6 or 7 moDCs were lysed in lysis buffer for 30 min on ice prior to being spun down at 9,400 × *g* for 5 min. The BCA protein assay (Pierce, Thermo Fisher scientific) was conducted to determine protein concentration and following the manufacturer’s instructions, and for each sample, 20 µg of total protein was diluted using SDS sample buffer (62.5 mM Tris, pH 6.8, 2% SDS, 10% glycerol).

Proteins were separated by SDS-PAGE and blotted onto PVDF membranes. Membranes were blocked in TBS containing 3% BSA at room temperature for 1 h prior to be stained with specific antibodies. Antibody signals were detected with fluorophore-coupled secondary antibodies and developed using Odyssey CLx (LI-COR) following the manufacturer’s instructions. Images were retrieved using Image Studio Lite 5.0 software. The following primary antibodies were used for western blotting: goat anti-galectin-9 (AF2045; R&D Systems, Minneapolis, Minnesota) at 1:1,000 (vol/vol), rabbit anti-phospho-(Thr 423) Pak1 (#2601; Cell Signaling Technology) at 1:500 (vol/vol), rabbit anti-Pak1 (#2602; Cell Signaling Technology) at 1:500 (vol/vol), rabbit anti-GAPDH (#2118; Cell Signaling Technology) at 1:500 (vol/vol), mouse anti-pMLC (#3675; Cell Signaling Technology) at 1:500 (vol/vol), rabbit anti-mDia (#DP4471; BD/ECM Biosciences) at 1:500 (vol/vol), and rat anti-tubulin (Novus Biological) at 1:2,000 (vol/vol). The following secondary antibodies were used: donkey anti-goat IRDye 680 (920-32224; LI-COR), donkey anti-rabbit IRDye 800 (926-32213; LI-COR), donkey anti-rabbit IRDye 680 (926-68073; LI-COR), goat anti-rabbit IRDye 800 (926-32211; LI-COR), goat anti-rat IRDye 680 (A21096; Invitrogen), donkey anti-mouse IRDye 680 (926-68072; LI-COR). All secondary antibodies were used at 1:5,000 (vol/vol).

### dSTORM acquisition

dSTORM was performed as follows. 100,000 day 8 matured WT, galectin-9 KD, and galectin-9 KD + rGal-9 moDCs were resuspended in 100 µl PBS and placed on poly-L-lysine–coated #1.5 German glass coverslips (#72290-12; Electron Microscopy Sciences) for 30 min at 4°C before fixation with 4% PFA +0.1% glutaraldehyde in 0.2 M phosphate buffer, pH 7.4, for 30 min at room temperature. For subsequent imaging, coverslips were washed with PBS and quenched with 0.1% Triton X-100, 100 mM glycine, 100 mM NH_4_Cl in PBS for 15 min at room temperature. Cells were then blocked for 1 h and subsequently stained with anti-CD44 antibody (10 µg/ml, #NBP1-47386AF647; Novus Biologicals) in 50 mM glycine + 3% BSA + 2% human serum. dSTORM acquisition and analysis were performed as described before (Neviani et al., 2020). Briefly, coverslips were washed with PBS and mounted in OxEA buffer. Localization data were extracted using ThunderSTORM processing in Fiji, and pair correlation analysis was performed with the SpatStat package in R.

### Data analysis

Cell tracking was performed using the manual tracking plugin of Fiji (ImageJ) with adjusted microscope-specific time and calibration parameters. Individual cells were tracked for at least 90 min 5 h after being embedded in the collagen to allow adaptation to the environment. The MSD over time intervals was determined as previously described (van Rijn et al., 2016). In short, the MSD was calculated per time interval for each cell. The average per time interval was calculated for all cells corrected for the tracking length of the cells. The Euclidean distance reached by a cell after 60 min of tracking with respect to their starting position was calculated using Chemotaxis and Migration software (Ibidi) after adjusting the X/Y calibration and time acquisition interval. Tracking plots were generated using Chemotaxis and Migration software (Ibidi). For time-lapse tracking experiments, the direction of movement was used to define the uropod rear as the space between the cell most distant rear point and the approximate location of the nucleus as defined by the widest point of the cell (the nucleus being the largest and stiffest organelle) (Janota et al., 2020). For leading edge and rear tracking, the leading edge was first tracked for the duration of the movie of that cell. The rear of the cell was then tracked for that same cell, for the duration of the movie of that cell.

Cell directionality was quantified by measuring the angle of cell displacement relative to the direction of the CCL21 gradient. An angle of 0° indicates movement directly up the gradient, the absence of directionality results in an average angle of 90°, and upon chemotaxis, lower angles will be more frequent. The border of the CCL21-expressing region was identified on the microscope image and used as the reference for analysis. Tracks were then rotated and shifted such that the border aligned with the x axis and the chemokine gradient with the y axis.

For the pathway enrichment analysis, we identified the top 50 genes correlating with the *lgals9* gene across all immune cells using the ULI RNA-seq dataset (GSE109125) and the gene constellation tool from the ImmGen. We then conducted a functional pathway enrichment analysis using gProfiler (Kolberg et al., 2023) across the Reactome dataset (Griss et al., 2020).

For actin and pMLC subcellular analysis, cells were imaged using a 1,024 × 1,024 ROI and a maximum-intensity z-project was performed using ImageJ of all channels. The distance from the cell front or rear edge to the nucleus was manually calculated using the line tool in ImageJ for the rear and front polarized positions of the cell (where rear was always the shorter distance). For actin intensity at rear and front positions, the actin channel (488) of the maximum-intensity projection was analyzed with the 3D surface plot in ImageJ, using a grid size of 128, a smoothing of 3.0, and no further scaling; the intensity at the regions corresponding to the near and far regions was then manually read from this 3D surface plot. Similar 3D surface plot analysis was performed for pMLC intensity quantification, where images were subjected to 2 × 2 binning while acquiring. For the line profile analysis of actin, the ImageJ macro from Hetmanski et al. (2025) was adapted. The 488 phalloidin channel was used to threshold the cells, with background pixels assigned NaN and the Fill Holes command used to include the whole cells in the quantified region. The thresholded binary image was then multiplied by the raw 488-phalloidin channel so that all the pixels within the cell had an actin intensity value and all the background pixels were set to NaN. Then, the line profiles from the rearmost and forwardmost points of the cell were created for a line wider than the total width of the cell, such that each value represented the average actin intensity across the entire width of the cell for that pixel position relative to the rear or front. The from-rear and from-front 220-pixel-long line profiles averaged across 15 cells in each condition were plotted, corresponding to the rear and front 25 μm of the cell.

All data were processed using Excel 2019 (Microsoft) and plotted using GraphPad Prism 8 software. All statistical analysis was done using Prism 8. All data are expressed as mean ± SEM unless otherwise stated. The statistical test used to analyze each data set is described in the corresponding figure legend. Statistical significance was considered for P values <0.05.

### Online supplemental material

Fig. S1 shows that galectin-9 depletion does not result in maturation defects in moDCs. It also depicts data demonstrating an impairment in chemotactic migration toward CCL21 in transwell assays and individual donors analyzed in main Fig. 1 H. Fig. S2 shows that impairment in migration toward tumor spheroids upon galectin-9 depletion is not dependent on the tumor cell line, and knockdown of galectin-9 diminishes DC migration at all maturation stages. Fig. S3 shows Rho GTPase–mediated pathways positively correlate with *lgals9* expression using GSE109125 dataset grouped by secondary correlation. Fig. S4 shows nucleus distance to the front and rear edges of migrating WT, gal9 KD, and gal9 KD + rGal9 DCs embedded in 3D collagen matrices. It also shows protein levels (by western blot) for pMLC, and total mDia in WT, galectin-9 depleted, and gal9 KD + rGal9 moDCs. Fig. S5 shows gating strategy followed to determine cDC2 purity after isolation from peripheral blood mononuclear cells, as well as surface galectin-9 and CCR7 expression in cDC2s. Video 1 shows representative time-lapse confocal microscopy of a WT moDC randomly migrating on a 3D collagen matrix. Video 2 shows time-lapse confocal microscopy of a gal9 KD moDC randomly migrating on a 3D collagen matrix. Movie shows that knocking down galectin-9 results in a retraction defect of the cell rear. Video 3 shows representative time-lapse confocal microscopy of a galectin-9 KD moDC treated with the rGal9 protein for 48 h randomly migrating on a 3D collagen matrix. Movie shows that treatment with rGal9 protein can rescue uropod retraction defect in galectin-9–depleted moDCs. Video 4 shows representative actin staining (phalloidin) for WT (left cell) and galectin-9–depleted (right cell) DCs using SIM super-resolution.

## Supplementary Material

SourceData F5 is the source file for Fig. 5.

SourceData F7 is the source file for Fig. 7.

SourceData FS1 is the source file for Fig. S1.

SourceData FS4 is the source file for Fig. S4.

## Data Availability

All data are available from the corresponding author upon reasonable request. The code used to analyze the chemotaxis data (as well as all raw data) is available at https://github.com/KoertS/gal-9-directionality-analysis.git.

## References

[bib1] Acton, S.E., J.L. Astarita, D. Malhotra, V. Lukacs-Kornek, B. Franz, P.R. Hess, Z. Jakus, M. Kuligowski, A.L. Fletcher, K.G. Elpek, . 2012. Podoplanin-rich stromal networks induce dendritic cell motility via activation of the C-type lectin receptor CLEC-2. Immunity. 37:276–289. 10.1016/j.immuni.2012.05.022 22884313 PMC3556784

[bib2] Akbani, R., K.-F. Becker, N. Carragher, T. Goldstein, L. de Koning, U. Korf, L. Liotta, G.B. Mills, S.S. Nishizuka, M. Pawlak, . 2014. Realizing the promise of reverse phase protein arrays for clinical, translational, and basic research: A workshop report: The RPPA (reverse phase protein array) society. Mol. Cell. Proteomics 13:1625–1643. 10.1074/mcp.O113.034918 24777629 PMC4083105

[bib3] Arikawa, T., N. Saita, S. Oomizu, M. Ueno, A. Matsukawa, S. Katoh, K. Kojima, K. Nagahara, M. Miyake, A. Yamauchi, . 2010. Galectin-9 expands immunosuppressive macrophages to ameliorate T-cell-mediated lung inflammation. Eur. J. Immunol. 40:548–558. 10.1002/eji.200939886 19902429

[bib4] Banchereau, J., and R.M. Steinman. 1998. Dendritic cells and the control of immunity. Nature. 392:245–252. 10.1038/32588 9521319

[bib5] Benvenuti, F., S. Hugues, M. Walmsley, S. Ruf, L. Fetler, M. Popoff, V.L.J. Tybulewicz, and S. Amigorena. 2004. Requirement of Rac1 and Rac2 expression by mature dendritic cells for T cell priming. Science. 305:1150–1153. 10.1126/science.1099159 15326354

[bib6] Bi, S., P.W. Hong, B. Lee, and L.G. Baum. 2011. Galectin-9 binding to cell surface protein disulfide isomerase regulates the redox environment to enhance T-cell migration and HIV entry. Proc. Natl. Acad. Sci. USA. 108:10650–10655. 10.1073/pnas.1017954108 21670307 PMC3127870

[bib7] Bourguignon, L.Y.W. 2008. Hyaluronan-mediated CD44 activation of RhoGTPase signaling and cytoskeleton function promotes tumor progression. Semin. Cancer Biol. 18:251–259. 10.1016/j.semcancer.2008.03.007 18450475 PMC2505114

[bib8] Bourguignon, L.Y.W., P.A. Singleton, H. Zhu, and F. Diedrich. 2003. Hyaluronan-mediated CD44 interaction with RhoGEF and Rho kinase promotes Grb2-associated binder-1 phosphorylation and phosphatidylinositol 3-kinase signaling leading to cytokine (macrophage-colony stimulating factor) production and breast tumor progression. J. Biol. Chem. 278:29420–29434. 10.1074/jbc.M301885200 12748184

[bib9] Bourguignon, L.Y.W., G. Wong, C. Earle, K. Krueger, and C.C. Spevak. 2010. Hyaluronan-CD44 interaction promotes c-Src-mediated twist signaling, microRNA-10b expression, and RhoA/RhoC up-regulation, leading to rho-kinase-associated cytoskeleton activation and breast tumor cell invasion. J. Biol. Chem. 285:36721–36735. 10.1074/jbc.M110.162305 20843787 PMC2978601

[bib10] Cao, A., N. Alluqmani, F.H.M. Buhari, L. Wasim, L.K. Smith, A.T. Quaile, M. Shannon, Z. Hakim, H. Furmli, D.M. Owen, . 2018. Galectin-9 binds IgM-BCR to regulate B cell signaling. Nat. Commun. 9:3288. 10.1038/s41467-018-05771-8 30120235 PMC6098130

[bib11] Caswell, P.T., and T. Zech. 2018. Actin-based cell protrusion in a 3D matrix. Trends Cell Biol. 28:823–834. 10.1016/j.tcb.2018.06.003 29970282 PMC6158345

[bib12] Clayton, N.S., and A.J. Ridley. 2020. Targeting rho GTPase signaling networks in cancer. Front. Cell Dev. Biol. 8:222. 10.3389/fcell.2020.00222 32309283 PMC7145979

[bib13] Coppin, L., A. Vincent, F. Frénois, B. Duchêne, F. Lahdaoui, L. Stechly, F. Renaud, Villenet C., I. Van Seuningen, E. Leteurtre, . 2017. Galectin-3 is a non-classic RNA binding protein that stabilizes the mucin MUC4 mRNA in the cytoplasm of cancer cells. Sci. Rep. 7:43927. 10.1038/srep43927 28262838 PMC5338267

[bib14] Dai, S.-Y., R. Nakagawa, A. Itoh, H. Murakami, Y. Kashio, H. Abe, S. Katoh, K. Kontani, M. Kihara, S.-L. Zhang, . 2005. Galectin-9 induces maturation of human monocyte-derived dendritic cells. J. Immunol. 175:2974–2981. 10.4049/jimmunol.175.5.2974 16116184

[bib15] Dardalhon, V., A.C. Anderson, J. Karman, L. Apetoh, R. Chandwaskar, D.H. Lee, M. Cornejo, N. Nishi, A. Yamauchi, F.J. Quintana, . 2010. Tim-3/galectin-9 pathway: Regulation of Th1 immunity through promotion of CD11b^+^Ly^−^6G^+^ myeloid cells. J. Immunol. 185:1383–1392. 10.4049/jimmunol.0903275 20574007 PMC2925247

[bib16] de Vries, I.J.M., A.A.O. Eggert, N.M. Scharenborg, J.L.M. Vissers, W.J. Lesterhuis, O.C. Boerman, C.J.A. Punt, G.J. Adema, and C.G. Figdor. 2002. Phenotypical and functional characterization of clinical grade dendritic cells. J. Immunother. 25:429–438. 10.1097/00002371-200209000-00007 12218781

[bib17] Delgado, M.-G., and A.-M. Lennon-Dumenil. 2022. How cell migration helps immune sentinels. Front. Cell Dev. Biol. 10:932472. 10.3389/fcell.2022.932472 36268510 PMC9577558

[bib18] Dunsmore, G., E.P. Rosero, S. Shahbaz, D.M. Santer, J. Jovel, P. Lacy, S. Houston, and S. Elahi. 2021. Neutrophils promote T-cell activation through the regulated release of CD44-bound Galectin-9 from the cell surface during HIV infection. PLoS Biol. 19:e3001387. 10.1371/journal.pbio.3001387 34411088 PMC8407585

[bib19] Friedl, P., and E.-B. Bröcker. 2004. Reconstructing leukocyte migration in 3D extracellular matrix by time-lapse videomicroscopy and computer-assisted tracking. Methods Mol. Biol. 239:77–90. 10.1385/1-59259-435-2:77 14573911

[bib20] Giovannone, N., J. Liang, A. Antonopoulos, J. Geddes Sweeney, S.L. King, S.M. Pochebit, N. Bhattacharyya, G.S. Lee, A. Dell, H.R. Widlund, . 2018. Galectin-9 suppresses B cell receptor signaling and is regulated by I-branching of N-glycans. Nat. Commun. 9:3287. 10.1038/s41467-018-05770-9 30120234 PMC6098069

[bib21] Griss, J., G. Viteri, K. Sidiropoulos, V. Nguyen, A. Fabregat, and H. Hermjakob. 2020. ReactomeGSA - Efficient multi-omics comparative pathway analysis. Mol. Cell. Proteomics 19:2115–2125. 10.1074/mcp.TIR120.002155 32907876 PMC7710148

[bib22] Heasman, S.J., L.M. Carlin, S. Cox, T. Ng, and A.J. Ridley. 2010. Coordinated RhoA signaling at the leading edge and uropod is required for T cell transendothelial migration. J. Cell Biol. 190:553–563. 10.1083/jcb.201002067 20733052 PMC2928012

[bib23] Hetmanski, J.H.R., M.J. Jones, M. Hartshorn, P.T. Caswell, and M.C. Jones. 2025. Differential roles of cyclin-CDK1 complexes in cell migration and invasion. J. Cell Sci. 138:jcs263697. 10.1242/jcs.263697 40518827 PMC12301658

[bib24] Hind, L.E., W.J.B. Vincent, and A. Huttenlocher. 2016. Leading from the back: The role of the uropod in neutrophil polarization and migration. Dev. Cell. 38:161–169. 10.1016/j.devcel.2016.06.031 27459068 PMC4982870

[bib25] Ho, J., T. Tumkaya, S. Aryal, H. Choi, and A. Claridge-Chang. 2019. Moving beyond P values: Data analysis with estimation graphics. Nat. Methods. 16:565–566. 10.1038/s41592-019-0470-3 31217592

[bib26] Hong, M.-H., I.-C. Weng, F.-Y. Li, W.-H. Lin, and F.-T. Liu. 2021. Intracellular galectins sense cytosolically exposed glycans as danger and mediate cellular responses. J. Biomed. Sci. 28:16. 10.1186/s12929-021-00713-x 33663512 PMC7931364

[bib27] Hsu, D.K., A.I. Chernyavsky, H.-Y. Chen, L. Yu, S.A. Grando, and F.-T. Liu. 2009. Endogenous galectin-3 is localized in membrane lipid rafts and regulates migration of dendritic cells. J. Invest. Dermatol. 129:573–583. 10.1038/jid.2008.276 18843294 PMC2645233

[bib28] Hsu, Y.-L., M.-Y. Wang, L.-J. Ho, C.-Y. Huang, and J.-H. Lai. 2015. Up-regulation of galectin-9 induces cell migration in human dendritic cells infected with dengue virus. J. Cell. Mol. Med. 19:1065–1076. 10.1111/jcmm.12500 25754930 PMC4420608

[bib29] Imai, K., Y. Minamiya, S. Koyota, M. Ito, H. Saito, Y. Sato, S. Motoyama, T. Sugiyama, and J.-I. Ogawa. 2012. Inhibition of dendritic cell migration by transforming growth factor-β1 increases tumor-draining lymph node metastasis. J. Exp. Clin. Cancer Res. 31:3. 10.1186/1756-9966-31-3 22233831 PMC3298529

[bib30] Iqbal, A.J., F. Krautter, I.A. Blacksell, R.D. Wright, S.N. Austin-Williams, M.-B. Voisin, M.T. Hussain, H.L. Law, T. Niki, M. Hirashima, . 2022. Galectin-9 mediates neutrophil capture and adhesion in a CD44 and β2 integrin-dependent manner. FASEB J. 36:e22065. 10.1096/fj.202100832R 34847625

[bib31] Janota, C.S., F.J. Calero-Cuenca, and E.R. Gomes. 2020. The role of the cell nucleus in mechanotransduction. Curr. Opin. Cell Biol. 63:204–211. 10.1016/j.ceb.2020.03.001 32361559

[bib32] Johannes, L., R. Jacob, and H. Leffler. 2018. Galectins at a glance. J. Cell Sci. 131:jcs208884. 10.1242/jcs.208884 29717004

[bib33] John, S., and R. Mishra. 2016. Galectin-9: From cell biology to complex disease dynamics. J. Biosci. 41:507–534. 10.1007/s12038-016-9616-y 27581941

[bib34] Johnson, L.A., S. Banerji, B.C. Lagerholm, and D.G. Jackson. 2021. Dendritic cell entry to lymphatic capillaries is orchestrated by CD44 and the hyaluronan glycocalyx. Life Sci. Alliance. 4:e202000908. 10.26508/lsa.202000908 33687996 PMC8008951

[bib35] Kashyap, A.S., L. Fernandez-Rodriguez, Y. Zhao, G. Monaco, M.P. Trefny, N. Yoshida, K. Martin, A. Sharma, N. Olieric, P. Shah, . 2019. GEF-H1 signaling upon microtubule destabilization is required for dendritic cell activation and specific anti-tumor responses. Cell Rep. 28:3367–3380.e8. 10.1016/j.celrep.2019.08.057 31553907 PMC6876861

[bib36] Kataoka, Y., Y. Ohshio, K. Teramoto, T. Igarashi, T. Asai, and J. Hanaoka. 2019. Hypoxia-induced galectin-3 enhances RhoA function to activate the motility of tumor cells in non-small cell lung cancer. Oncol. Rep. 41:853–862. 10.3892/or.2018.6915 30535445 PMC6312936

[bib37] Kolberg, L., U. Raudvere, I. Kuzmin, P. Adler, J. Vilo, and H. Peterson. 2023. g:Profiler-interoperable web service for functional enrichment analysis and gene identifier mapping (2023 update). Nucleic Acids Res. 51:W207–W212. 10.1093/nar/gkad347 37144459 PMC10320099

[bib38] Kopf, A., J. Renkawitz, R. Hauschild, I. Girkontaite, K. Tedford, J. Merrin, O. Thorn-Seshold, D. Trauner, H. Häcker, K.-D. Fischer, . 2020. Microtubules control cellular shape and coherence in amoeboid migrating cells. J. Cell Biol. 219:e201907154. 10.1083/jcb.201907154 32379884 PMC7265309

[bib39] Krendel, M., F.T. Zenke, and G.M. Bokoch. 2002. Nucleotide exchange factor GEF-H1 mediates cross-talk between microtubules and the actin cytoskeleton. Nat. Cell Biol. 4:294–301. 10.1038/ncb773 11912491

[bib40] Kroll, J., R. Hauschild, A. Kuznetcov, K. Stefanowski, M.D. Hermann, J. Merrin, L. Shafeek, A. Müller-Taubenberger, and J. Renkawitz. 2023. Adaptive pathfinding by nucleokinesis during amoeboid migration. EMBO J. 42:e114557. 10.15252/embj.2023114557 37987147 PMC10711653

[bib41] Lakshminarayan, R., C. Wunder, U. Becken, M.T. Howes, C. Benzing, S. Arumugam, S. Sales, N. Ariotti, V. Chambon, C. Lamaze, . 2014. Galectin-3 drives glycosphingolipid-dependent biogenesis of clathrin-independent carriers. Nat. Cell Biol. 16:595–606. 10.1038/ncb2970 24837829

[bib42] Lämmermann, T., B.L. Bader, S.J. Monkley, T. Worbs, R. Wedlich-Söldner, K. Hirsch, M. Keller, R. Förster, D.R. Critchley, R. Fässler, and M. Sixt. 2008. Rapid leukocyte migration by integrin-independent flowing and squeezing. Nature. 453:51–55. 10.1038/nature06887 18451854

[bib43] Lämmermann, T., J. Renkawitz, X. Wu, K. Hirsch, C. Brakebusch, and M. Sixt. 2009. Cdc42-dependent leading edge coordination is essential for interstitial dendritic cell migration. Blood. 113:5703–5710. 10.1182/blood-2008-11-191882 19190242

[bib44] Lawson, C.D., and A.J. Ridley. 2018. Rho GTPase signaling complexes in cell migration and invasion. J. Cell Biol. 217:447–457. 10.1083/jcb.201612069 29233866 PMC5800797

[bib45] Lee, S., and S. Kumar. 2016. Actomyosin stress fiber mechanosensing in 2D and 3D. F1000 Res. 5:1000 Faculty Rev-2261. 10.12688/f1000research.8800.1 PMC5017290 27635242

[bib46] Leffler, H., S. Carlsson, M. Hedlund, Y. Qian, and F. Poirier. 2002. Introduction to galectins. Glycoconj. J. 19:433–440. 10.1023/B:GLYC.0000014072.34840.04 14758066

[bib47] Li, L., X. Xu, X. Wang, S. Zhang, W. Yao, J. Liu, Z. Liu, and P. Yang. 2023. Galectin-9 in synergy with NF-κB inhibition restores immune regulatory capability in dendritic cells of subjects with food allergy. Clin. Exp. Immunol. 213:155–163. 10.1093/cei/uxad062 37279535 PMC10361740

[bib48] Liu, F.-T., R.J. Patterson, and J.L. Wang. 2002. Intracellular functions of galectins. Biochim. Biophys. Acta. 1572:263–273. 10.1016/s0304-4165(02)00313-6 12223274

[bib49] Liu, F.-T., and S.R. Stowell. 2023. The role of galectins in immunity and infection. Nat. Rev. Immunol. 23:479–494. 10.1038/s41577-022-00829-7 36646848 PMC9842223

[bib50] Liu, J., X. Zhang, Y. Cheng, and X. Cao. 2021. Dendritic cell migration in inflammation and immunity. Cell. Mol. Immunol. 18:2461–2471. 10.1038/s41423-021-00726-4 34302064 PMC8298985

[bib51] Machacek, M., L. Hodgson, C. Welch, H. Elliott, O. Pertz, P. Nalbant, A. Abell, G.L. Johnson, K.M. Hahn, and G. Danuser. 2009. Coordination of Rho GTPase activities during cell protrusion. Nature. 461:99–103. 10.1038/nature08242 19693013 PMC2885353

[bib52] Matsumoto, R., H. Matsumoto, M. Seki, M. Hata, Y. Asano, S. Kanegasaki, R.L. Stevens, and M. Hirashima. 1998. Human ecalectin, a variant of human galectin-9, is a novel eosinophil chemoattractant produced by T lymphocytes. J. Biol. Chem. 273:16976–16984. 10.1074/jbc.273.27.16976 9642261

[bib53] Mayhew, M.W., E.D. Jeffery, N.E. Sherman, K. Nelson, J.M. Polefrone, S.J. Pratt, J. Shabanowitz, J.T. Parsons, J.W. Fox, D.F. Hunt, and A.F. Horwitz. 2007. Identification of phosphorylation sites in betaPIX and PAK1. J. Cell Sci. 120:3911–3918. 10.1242/jcs.008177 17989089 PMC4627702

[bib54] Meili, R., and R.A. Firtel. 2003. Two poles and a compass. Cell. 114:153–156. 10.1016/s0092-8674(03)00553-1 12887916

[bib55] Michaelson, D., J. Silletti, G. Murphy, P. D’Eustachio, M. Rush, and M.R. Philips. 2001. Differential localization of Rho GTPases in live cells: Regulation by hypervariable regions and RhoGDI binding. J. Cell Biol. 152:111–126. 10.1083/jcb.152.1.111 11149925 PMC2193662

[bib56] Neviani, V., S. van Deventer, T.P. Wörner, K.T. Xenaki, M. van de Waterbeemd, R.N.P. Rodenburg, I.M.N. Wortel, J.K. Kuiper, S. Huisman, J. Granneman, . 2020. Site-specific functionality and tryptophan mimicry of lipidation in tetraspanin CD9. FEBS J. 287:5323–5344. 10.1111/febs.15295 32181977 PMC7818406

[bib57] Pertz, O., L. Hodgson, R.L. Klemke, and K.M. Hahn. 2006. Spatiotemporal dynamics of RhoA activity in migrating cells. Nature. 440:1069–1072. 10.1038/nature04665 16547516

[bib58] Querol Cano, L., V.-M.E. Dunlock, F. Schwerdtfeger, and A.B. van Spriel. 2024. Membrane organization by tetraspanins and galectins shapes lymphocyte function. Nat. Rev. Immunol. 24:193–212. 10.1038/s41577-023-00935-0 37758850

[bib59] Querol Cano, L., O. Tagit, Y. Dolen, A. van Duffelen, S. Dieltjes, S.I. Buschow, T. Niki, M. Hirashima, B. Joosten, K. van den Dries, . 2019. Intracellular Galectin-9 controls dendritic cell function by maintaining plasma membrane rigidity. iScience. 22:240–255. 10.1016/j.isci.2019.11.019 31786520 PMC6906692

[bib60] Rahmati, A., S. Bigam, and S. Elahi. 2023. Galectin-9 promotes natural killer cells activity via interaction with CD44. Front. Immunol. 14:1131379. 10.3389/fimmu.2023.1131379 37006235 PMC10060867

[bib61] Randolph, G.J., V. Angeli, and M.A. Swartz. 2005. Dendritic-cell trafficking to lymph nodes through lymphatic vessels. Nat. Rev. Immunol. 5:617–628. 10.1038/nri1670 16056255

[bib62] Renkawitz, J., A. Kopf, J. Stopp, I. de Vries, M.K. Driscoll, J. Merrin, R. Hauschild, E.S. Welf, G. Danuser, R. Fiolka, and M. Sixt. 2019. Nuclear positioning facilitates amoeboid migration along the path of least resistance. Nature. 568:546–550. 10.1038/s41586-019-1087-5 30944468 PMC7217284

[bib63] Renkawitz, J., K. Schumann, M. Weber, T. Lämmermann, H. Pflicke, M. Piel, J. Polleux, J.P. Spatz, and M. Sixt. 2009. Adaptive force transmission in amoeboid cell migration. Nat. Cell Biol. 11:1438–1443. 10.1038/ncb1992 19915557

[bib64] Sánchez-Madrid, F., and J.M. Serrador. 2009. Bringing up the rear: Defining the roles of the uropod. Nat. Rev. Mol. Cell Biol. 10:353–359. 10.1038/nrm2680 19373240

[bib65] Santalla Méndez, R., A. Rodgers Furones, R. Classens, K. Fedorova, M. Haverdil, M. Canela Capdevila, A. van Duffelen, C.G. Spruijt, M. Vermeulen, M. Ter Beest, . 2023. Galectin-9 interacts with Vamp-3 to regulate cytokine secretion in dendritic cells. Cell Mol. Life Sci. 80:306. 10.1007/s00018-023-04954-x 37755527 PMC10533640

[bib66] Senbanjo, L.T., and M.A. Chellaiah. 2017. CD44: A multifunctional cell surface adhesion receptor is a regulator of progression and metastasis of cancer cells. Front. Cell Dev. Biol. 5:18. 10.3389/fcell.2017.00018 28326306 PMC5339222

[bib67] Shehwana, H., S.V. Kumar, J.M. Melott, M.A. Rohrdanz, C. Wakefield, Z. Ju, D.R. Siwak, Y. Lu, B.M. Broom, J.N. Weinstein, . 2022. Rppa space: An R package for normalization and quantitation of reverse-phase protein array data. Bioinformatics. 38:5131–5133. 10.1093/bioinformatics/btac665 36205581 PMC9665860

[bib68] Sil, P., N. Mateos, S. Nath, S. Buschow, C. Manzo, K.G.N. Suzuki, T. Fujiwara, A. Kusumi, M.F. Garcia-Parajo, and S. Mayor. 2020. Dynamic actin-mediated nano-scale clustering of CD44 regulates its meso-scale organization at the plasma membrane. Mol. Biol. Cell. 31:561–579. 10.1091/mbc.E18-11-0715 31577524 PMC7202065

[bib69] Siwak, D.R., J. Li, R. Akbani, H. Liang, and Y. Lu. 2019. Analytical platforms 3: Processing samples via the RPPA pipeline to generate large-scale data for clinical studies. Adv. Exp. Med. Biol. 1188:113–147. 10.1007/978-981-32-9755-5_7 31820386

[bib70] Skandalis, S.S. 2023. CD44 intracellular domain: A long tale of a short tail. Cancers. 15:5041. 10.3390/cancers15205041 37894408 PMC10605500

[bib71] Song, M.-S., J.-H. Nam, K.-E. Noh, and D.-S. Lim. 2024. Dendritic cell-based immunotherapy: The importance of dendritic cell migration. J. Immunol. Res. 2024:7827246. 10.1155/2024/7827246 38628676 PMC11019573

[bib72] Suszczyk, D., W. Skiba, A. Pawlowska, G. Polak, R. Tarkowski, and I. Wertel. 2023. Expression of Gal-9 on dendritic cells and soluble forms of TIM-3/Gal-9 in patients suffering from endometriosis. Int. J. Mol. Sci. 24:5948. 10.3390/ijms24065948 36983021 PMC10056739

[bib73] Szabo, K., D. Varga, A.G. Vegh, N. Liu, X. Xiao, L. Xu, L. Dux, M. Erdelyi, L. Rovo, and A. Keller-Pinter. 2022. Syndecan-4 affects myogenesis via Rac1-mediated actin remodeling and exhibits copy-number amplification and increased expression in human rhabdomyosarcoma tumors. Cell. Mol. Life Sci. 79:122. 10.1007/s00018-021-04121-0 35128576 PMC8818642

[bib74] Torreno-Pina, J.A., B.M. Castro, C. Manzo, S.I. Buschow, A. Cambi, and M.F. Garcia-Parajo. 2014. Enhanced receptor-clathrin interactions induced by N-glycan-mediated membrane micropatterning. Proc. Natl. Acad. Sci. USA. 111:11037–11042. 10.1073/pnas.1402041111 25030450 PMC4121791

[bib75] Türeci, O., H. Schmitt, N. Fadle, M. Pfreundschuh, and U. Sahin. 1997. Molecular definition of a novel human galectin which is immunogenic in patients with Hodgkin’s disease. J. Biol. Chem. 272:6416–6422. 10.1074/jbc.272.10.6416 9045665

[bib76] van Deventer, S., A.B. Arp, and A.B. van Spriel. 2021. Dynamic plasma membrane organization: A complex symphony. Trends Cell Biol. 31:119–129. 10.1016/j.tcb.2020.11.004 33248874

[bib77] van Rijn, A., L. Paulis, J. te Riet, A. Vasaturo, I. Reinieren-Beeren, A. van der Schaaf, A.J. Kuipers, L.P. Schulte, B.C. Jongbloets, R.J. Pasterkamp, . 2016. Semaphorin 7A promotes chemokine-driven dendritic cell migration. J. Immunol. 196:459–468. 10.4049/jimmunol.1403096 26597008

[bib78] Vargas, P., P. Maiuri, M. Bretou, P.J. Sáez, P. Pierobon, M. Maurin, M. Chabaud, D. Lankar, D. Obino, E. Terriac, . 2016. Innate control of actin nucleation determines two distinct migration behaviours in dendritic cells. Nat. Cell Biol. 18:43–53. 10.1038/ncb3284 26641718 PMC5885286

[bib79] Villablanca, E.J., L. Raccosta, D. Zhou, R. Fontana, D. Maggioni, A. Negro, F. Sanvito, M. Ponzoni, B. Valentinis, M. Bregni, . 2010. Tumor-mediated liver X receptor-alpha activation inhibits CC chemokine receptor-7 expression on dendritic cells and dampens antitumor responses. Nat. Med. 16:98–105. 10.1038/nm.2074 20037595

[bib80] Wang, Y., J. Sun, C. Ma, W. Gao, B. Song, H. Xue, W. Chen, X. Chen, Y. Zhang, Q. Shao, . 2016. Reduced expression of Galectin-9 contributes to a poor outcome in Colon cancer by inhibiting NK cell chemotaxis partially through the Rho/ROCK1 signaling pathway. PloS One. 11:e0152599. 10.1371/journal.pone.0152599 27028892 PMC4814049

[bib81] Warner, H., G. Franciosa, G. van der Borg, B. Coenen, F. Faas, C. Koenig, R. de Boer, R. Classens, S. Maassen, M.V. Baranov, . 2024. Atypical cofilin signaling drives dendritic cell migration through the extracellular matrix via nuclear deformation. Cell Rep. 43:113866. 10.1016/j.celrep.2024.113866 38416638

[bib82] Wculek, S.K., F.J. Cueto, A.M. Mujal, I. Melero, M.F. Krummel, and D. Sancho. 2020. Dendritic cells in cancer immunology and immunotherapy. Nat. Rev. Immunol. 20:7–24. 10.1038/s41577-019-0210-z 31467405

[bib83] Wolf, K., M. Te Lindert, M. Krause, S. Alexander, J. Te Riet, A.L. Willis, R.M. Hoffman, C.G. Figdor, S.J. Weiss, and P. Friedl. 2013. Physical limits of cell migration: Control by ECM space and nuclear deformation and tuning by proteolysis and traction force. J. Cell Biol. 201:1069–1084. 10.1083/jcb.201210152 23798731 PMC3691458

[bib84] Wong, K., O. Pertz, K. Hahn, and H. Bourne. 2006. Neutrophil polarization: Spatiotemporal dynamics of RhoA activity support a self-organizing mechanism. Proc. Natl. Acad. Sci. USA. 103:3639–3644. 10.1073/pnas.0600092103 16537448 PMC1450135

[bib85] Wortel, I.M.N., S. Kim, A.Y. Liu, E.C. Ibarra, and M.J. Miller. 2022. Listeria motility increases the efficiency of epithelial invasion during intestinal infection. PLoS Pathog. 18:e1011028. 10.1371/journal.ppat.1011028 36584235 PMC9836302

[bib86] Yang, N., J. Williams, V. Pekovic-Vaughan, P. Wang, S. Olabi, J. McConnell, N. Gossan, A. Hughes, J. Cheung, C.H. Streuli, and Q.J. Meng. 2017. Cellular mechano-environment regulates the mammary circadian clock. Nat. Commun. 8:14287. 10.1038/ncomms14287 28134247 PMC5290282

[bib87] Yang, R., L. Sun, C.-F. Li, Y.-H. Wang, J. Yao, H. Li, M. Yan, W.-C. Chang, J.M. Hsu, J.-H. Cha, . 2021. Galectin-9 interacts with PD-1 and TIM-3 to regulate T cell death and is a target for cancer immunotherapy. Nat. Commun. 12:832. 10.1038/s41467-021-21099-33547304 PMC7864927

[bib88] Zhang, C.-X., D.-J. Huang, V. Baloche, L. Zhang, J.-X. Xu, B.-W. Li, X.-R. Zhao, J. He, H.-Q. Mai, Q.-Y. Chen, . 2020. Galectin-9 promotes a suppressive microenvironment in human cancer by enhancing STING degradation. Oncogenesis. 9:65. 10.1038/s41389-020-00248-0 32632113 PMC7338349

[bib89] Zhang, W., B.P. Bhetwal, and S.J. Gunst. 2018. Rho kinase collaborates with p21-activated kinase to regulate actin polymerization and contraction in airway smooth muscle. J. Physiol. 596:3617–3635. 10.1113/JP275751 29746010 PMC6092288

[bib90] Zhang, Y., H. Xia, X. Ge, Q. Chen, D. Yuan, Q. Chen, W. Leng, L. Chen, Q. Tang, and F. Bi. 2014. CD44 acts through RhoA to regulate YAP signaling. Cell. Signal. 26:2504–2513. 10.1016/j.cellsig.2014.07.031 25101858

[bib91] Zhou, Y., B. Zhou, L. Pache, M. Chang, A.H. Khodabakhshi, O. Tanaseichuk, C. Benner, and S.K. Chanda. 2019. Metascape provides a biologist-oriented resource for the analysis of systems-level datasets. Nat. Commun. 10:1523. 10.1038/s41467-019-09234-6 30944313 PMC6447622

[bib92] Zhu, C., A.C. Anderson, A. Schubart, H. Xiong, J. Imitola, S.J. Khoury, X.X. Zheng, T.B. Strom, and V.K. Kuchroo. 2005. The Tim-3 ligand galectin-9 negatively regulates T helper type 1 immunity. Nat. Immunol. 6:1245–1252. 10.1038/ni1271 16286920

