# Supplementary figures and images for: Galectin-9 regulates dendritic cell polarity and uropod contraction by modulating RhoA activity
Source: J Cell Biol. 2025 Sep 23;224(11):e202404079. doi: 10.1083/jcb.202404079 (PMC12456409; doi:10.1083/jcb.202404079)

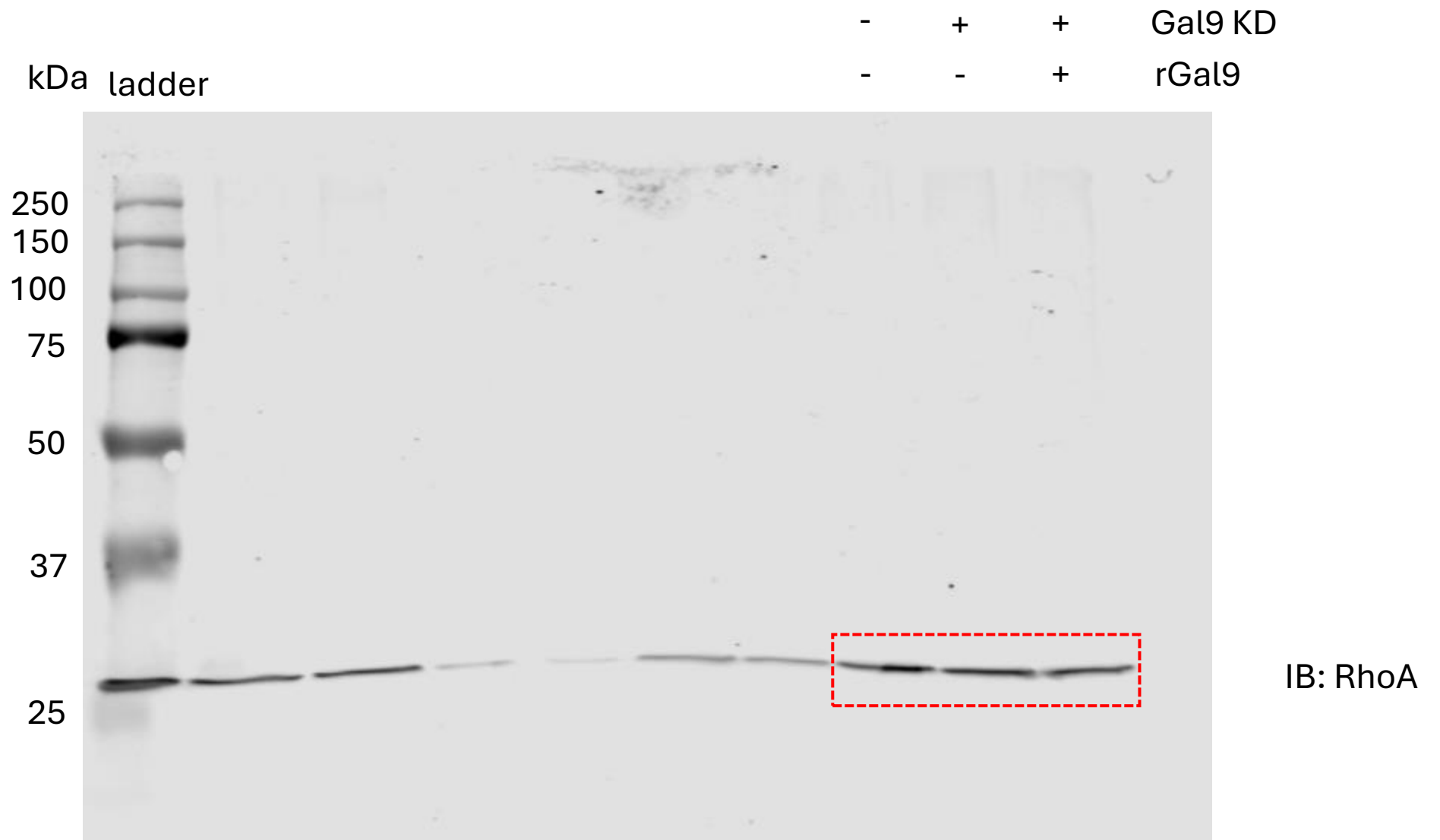

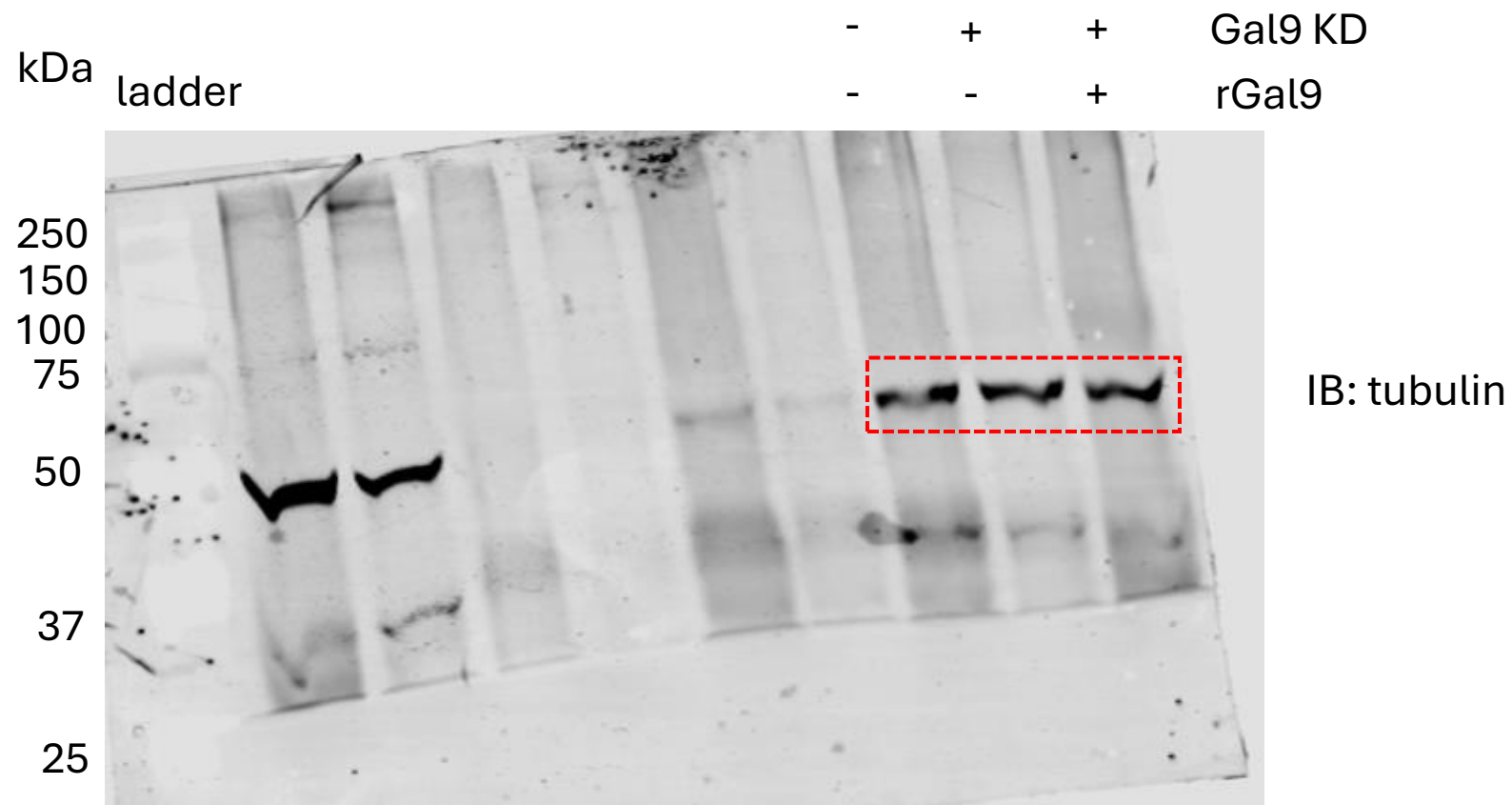

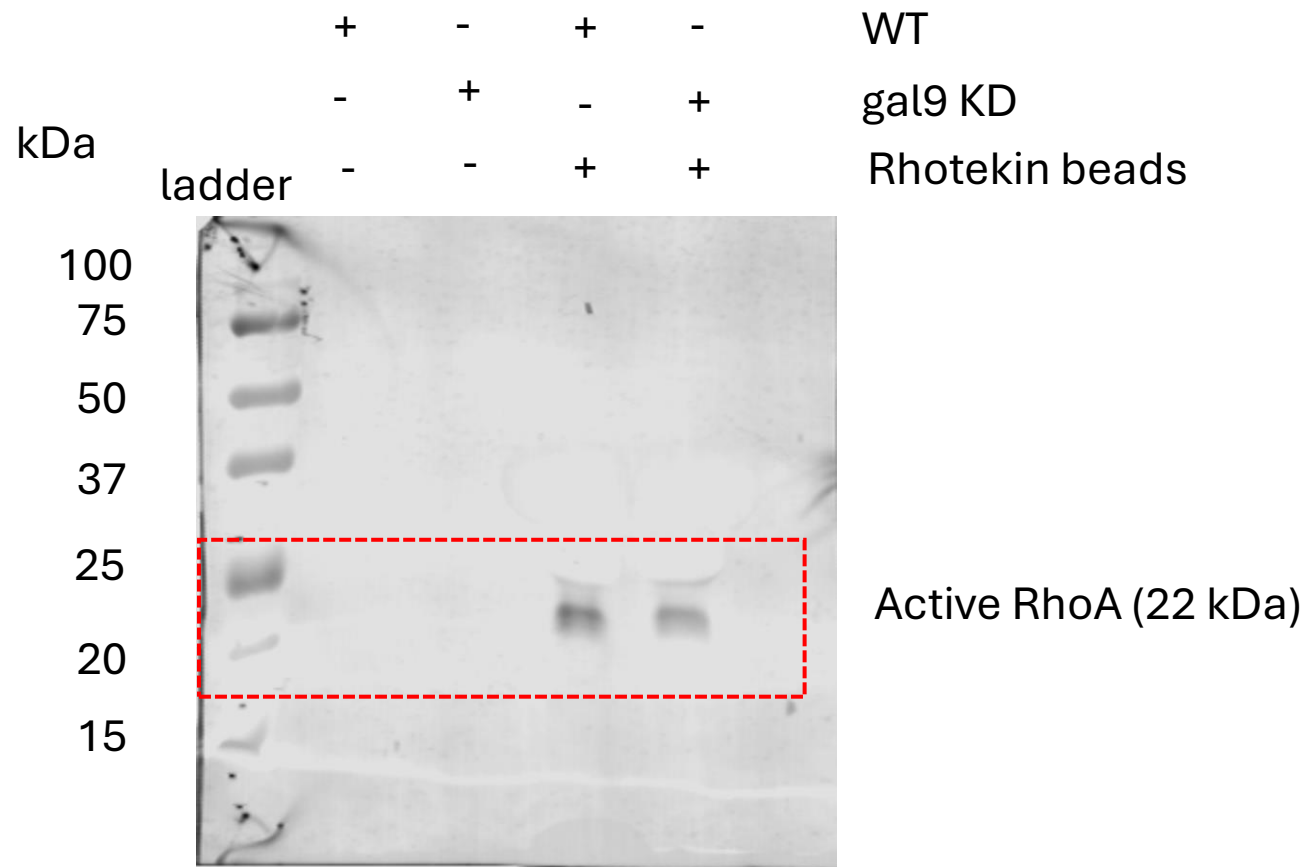

|     |        |   |   |   |         |
|-----|--------|---|---|---|---------|
|     |        | - | + | + | Gal9 KD |
| kDa | ladder | - | - | + | rGal9   |

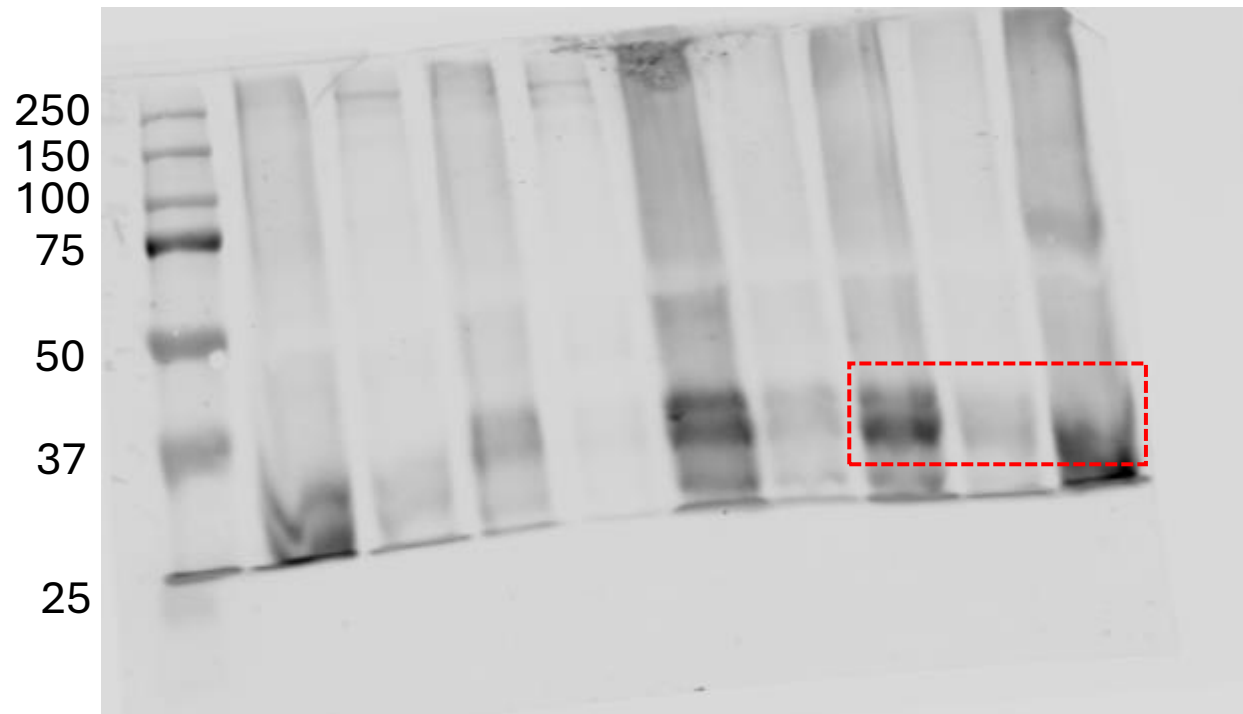

IB: galectin-9

Supplement: SourceData F5 — is the source file for Fig. 5. [file jcb_202404079_sourcedataf5.pdf]
