# Supplementary material for: Galectin-9 regulates dendritic cell polarity and uropod contraction by modulating RhoA activity
Source: J Cell Biol. 2025 Sep 23;224(11):e202404079. doi: 10.1083/jcb.202404079 (PMC12456409; doi:10.1083/jcb.202404079)
Supplement: SourceData F7 — is the source file for Fig. 7. [file jcb_202404079_sourcedataf7.pdf]

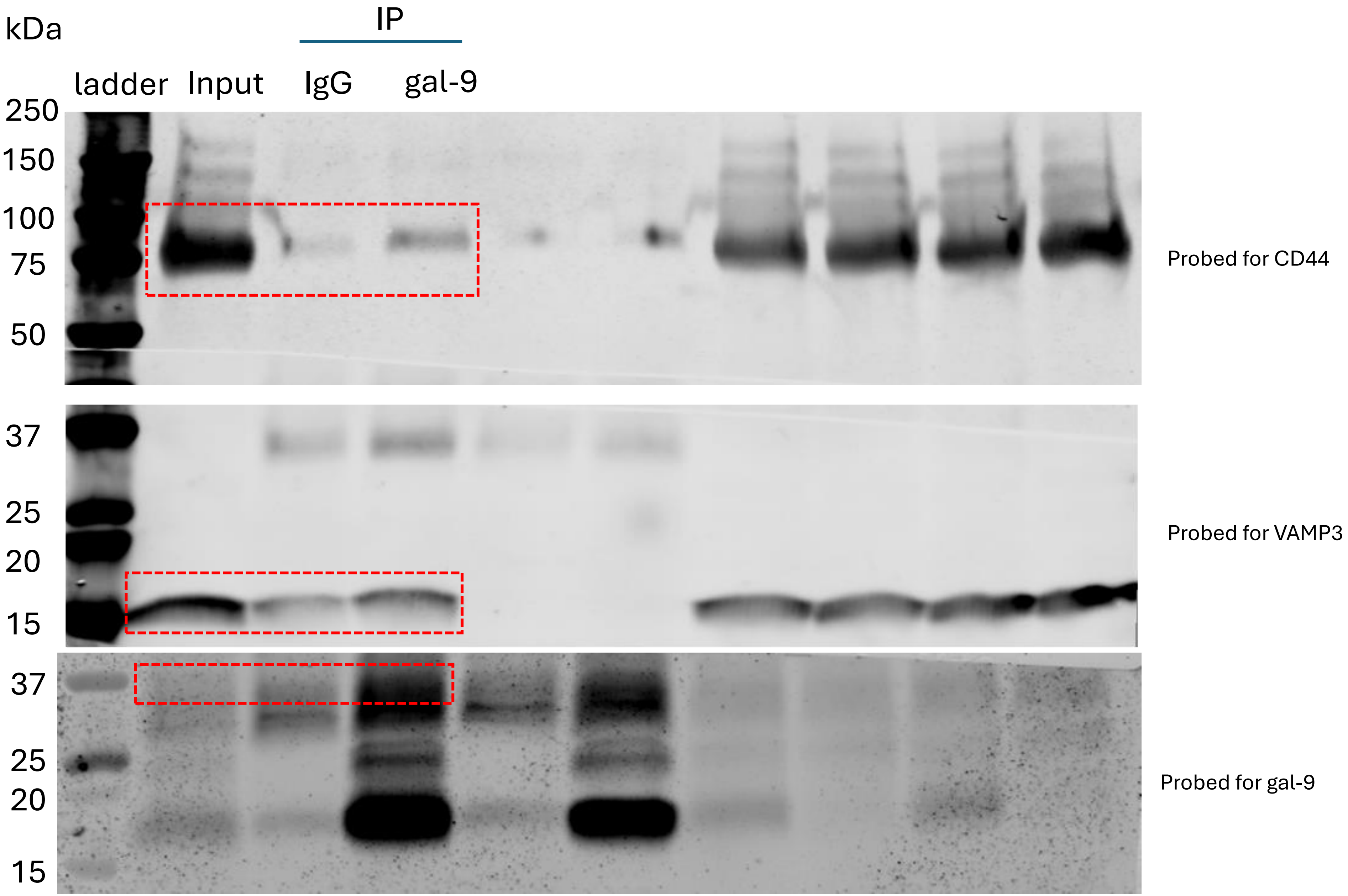
kDa
IP
ladder
Input
IgG
gal-9
250
150
100
75
50
37
25
20
15
37
25
20
15
Probed for CD44
Probed for VAMP3
Probed for gal-9

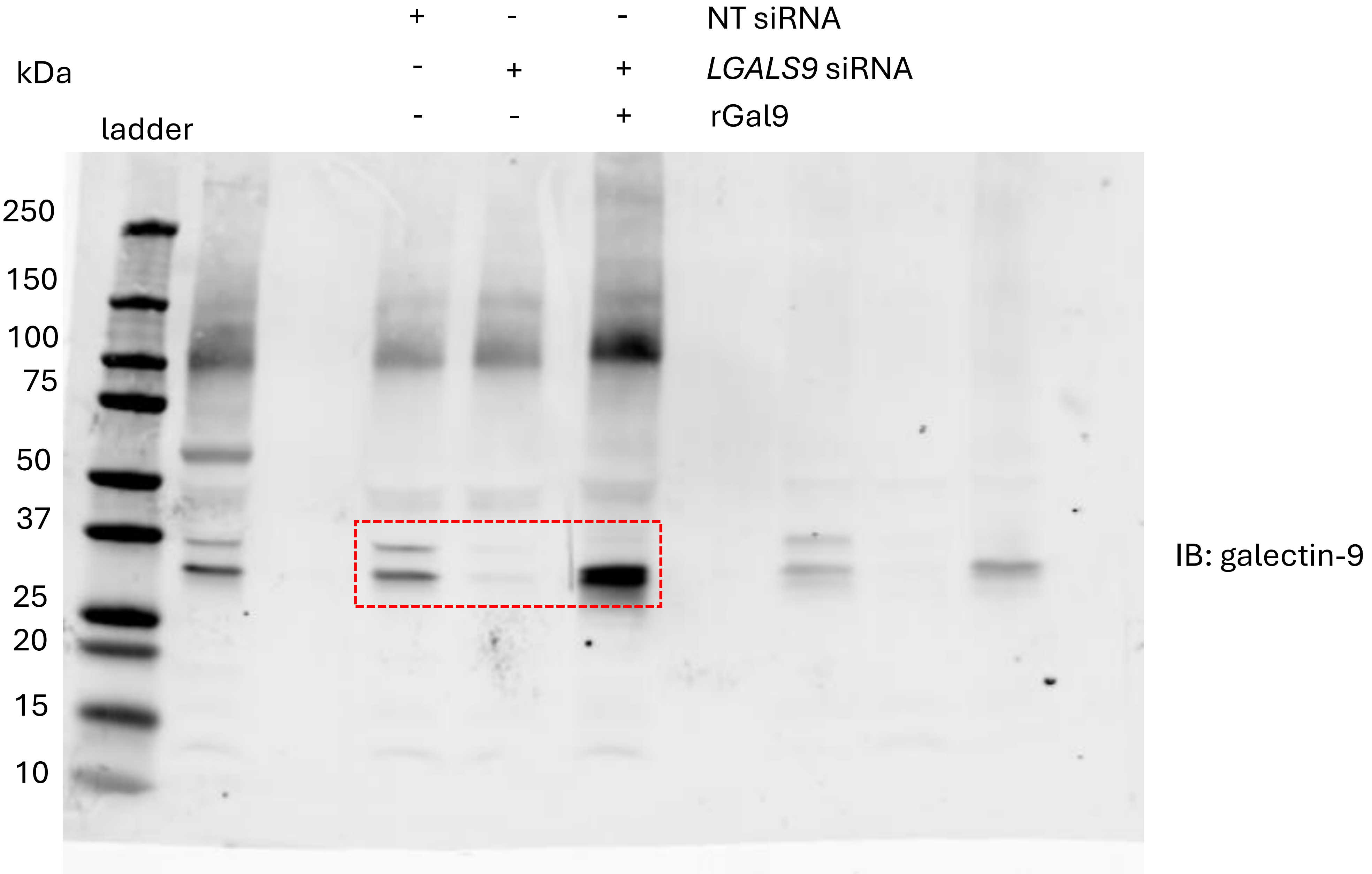
NT siRNA + - -
LGALS9 siRNA - + +
rGal9 - - +
kDa
ladder
250
150
100
75
50
37
25
20
15
10
IB: galectin-9

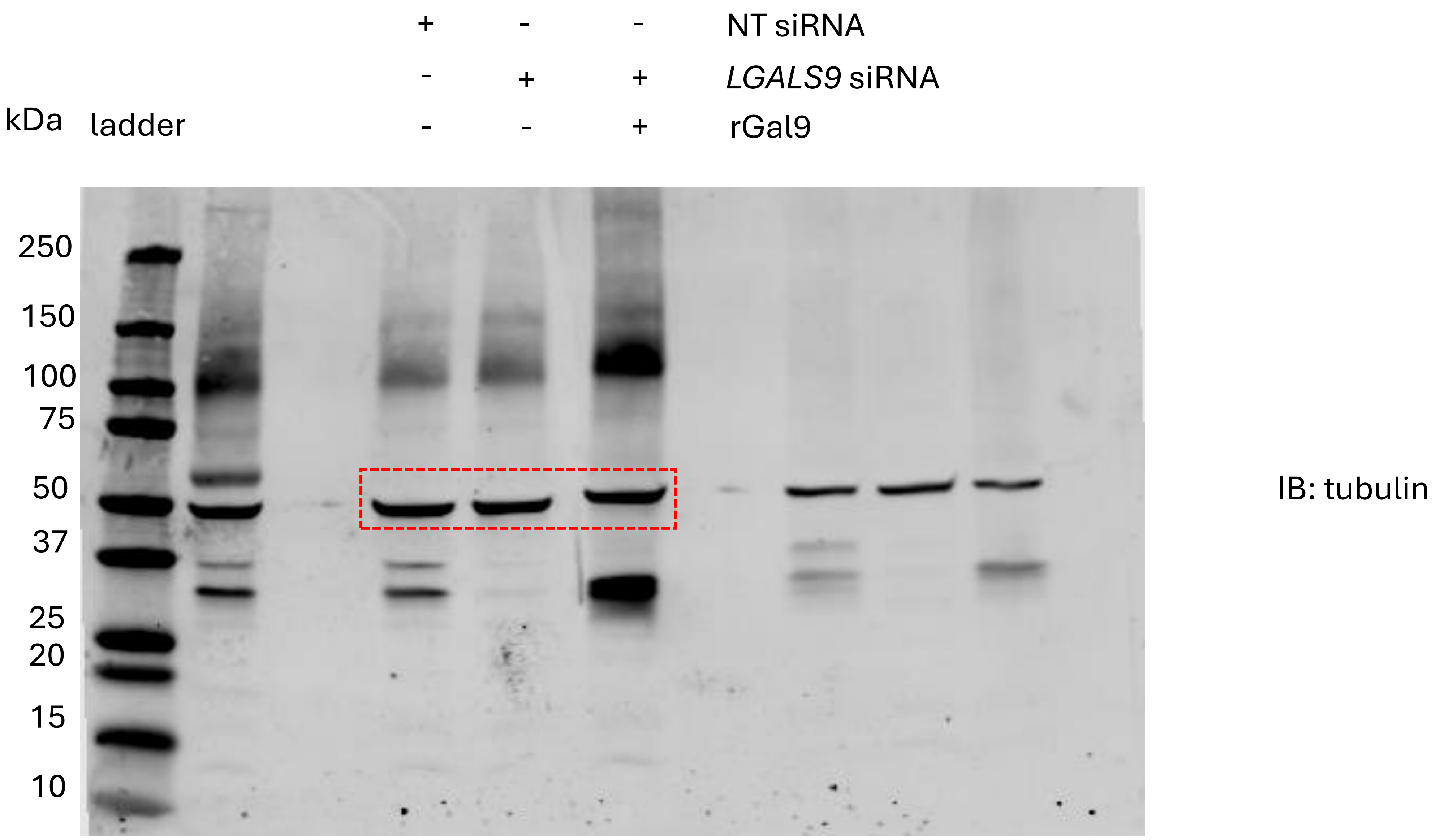
+ - - NT siRNA
- + + LGALS9 siRNA
kDa ladder - - + rGal9
250
150
100
75
50
37
25
20
15
10
IB: tubulin

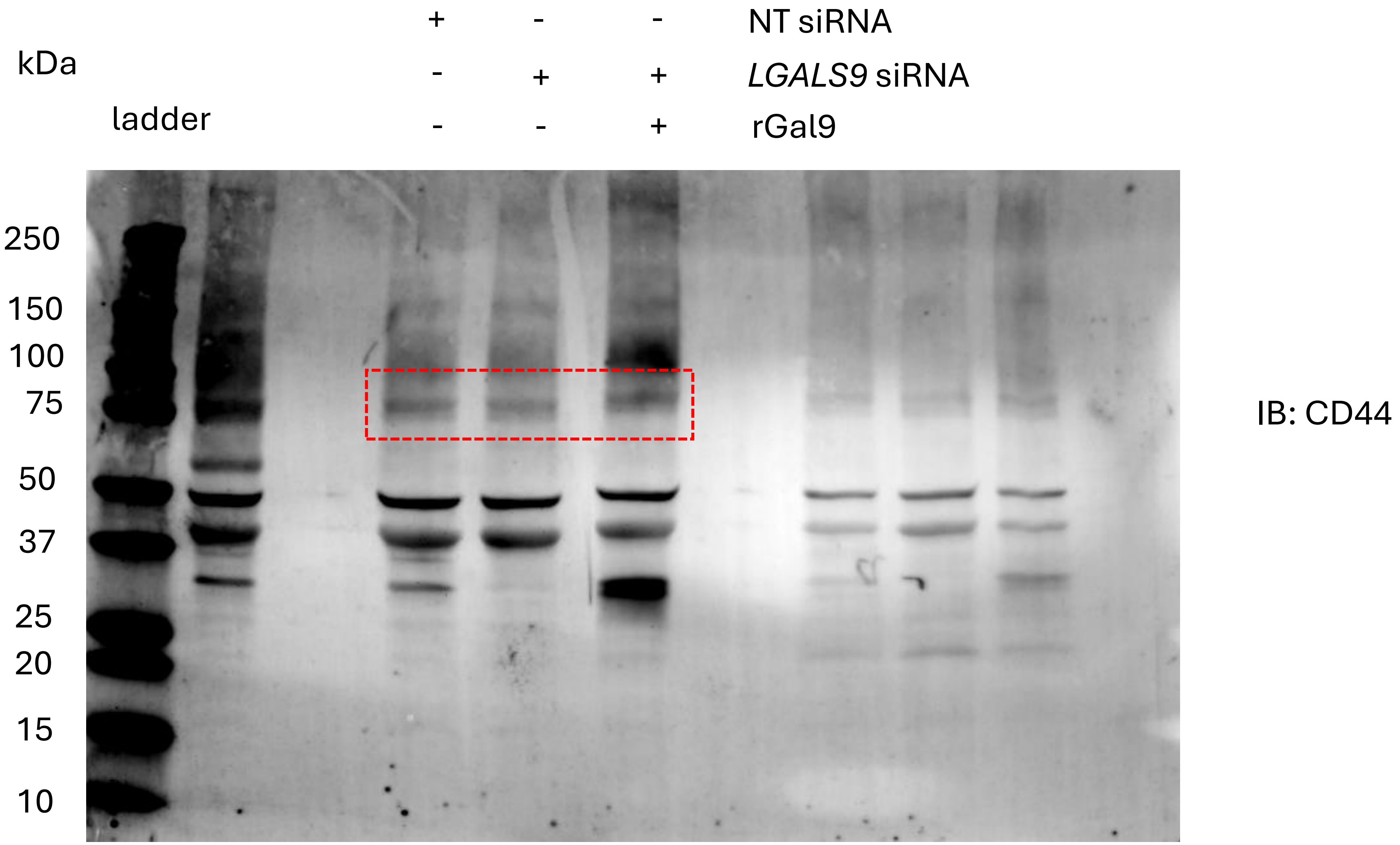
+ - - NT siRNA
- + + LGALS9 siRNA
- - + rGal9
kDa
ladder
250
150
100
75
50
37
25
20
15
10
IB: CD44

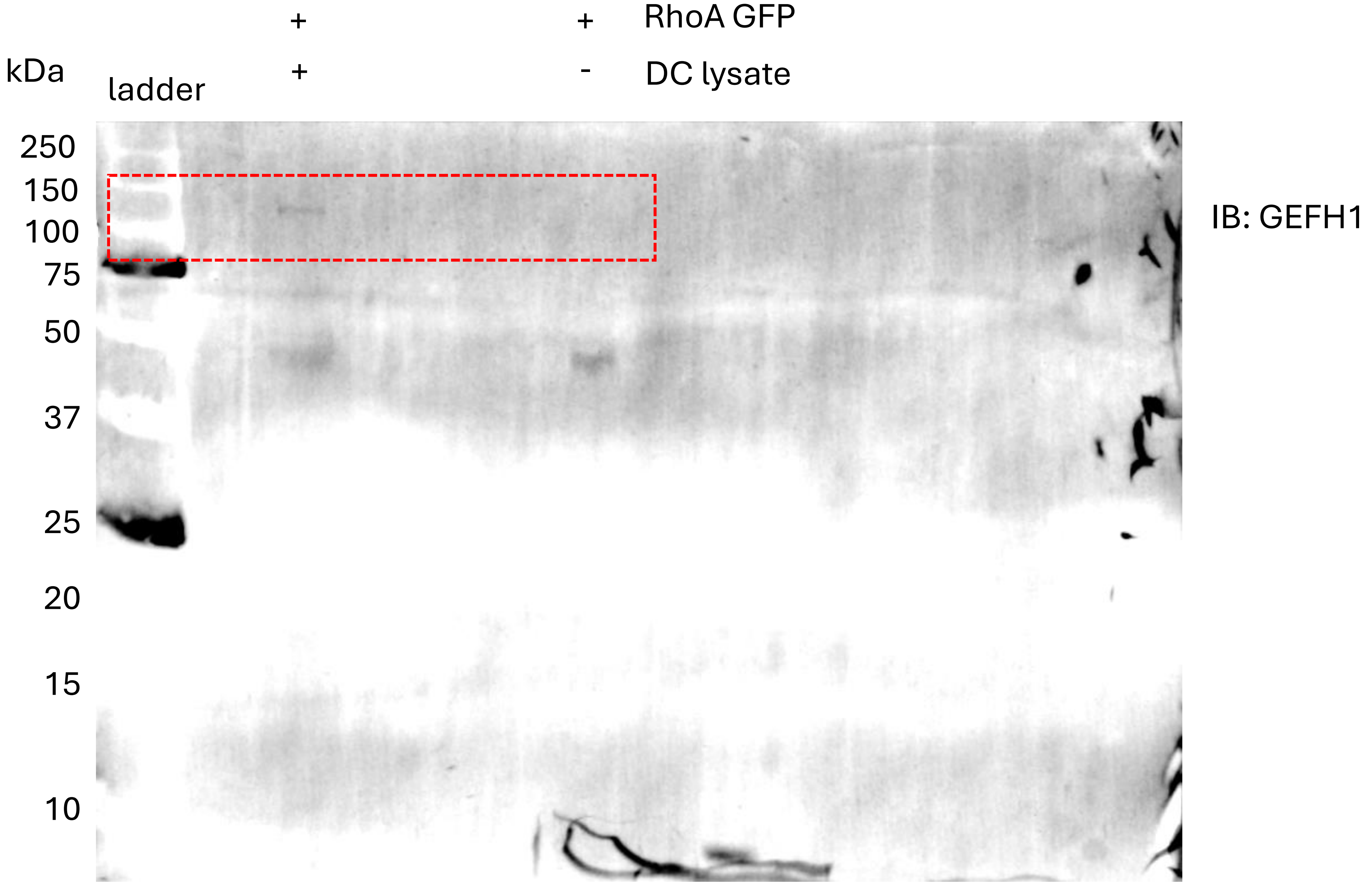

+ + RhoA GFP
kDa ladder + - DC lysate
250
150
100
75
50
37
25
20
15
10
IB: GEFH1

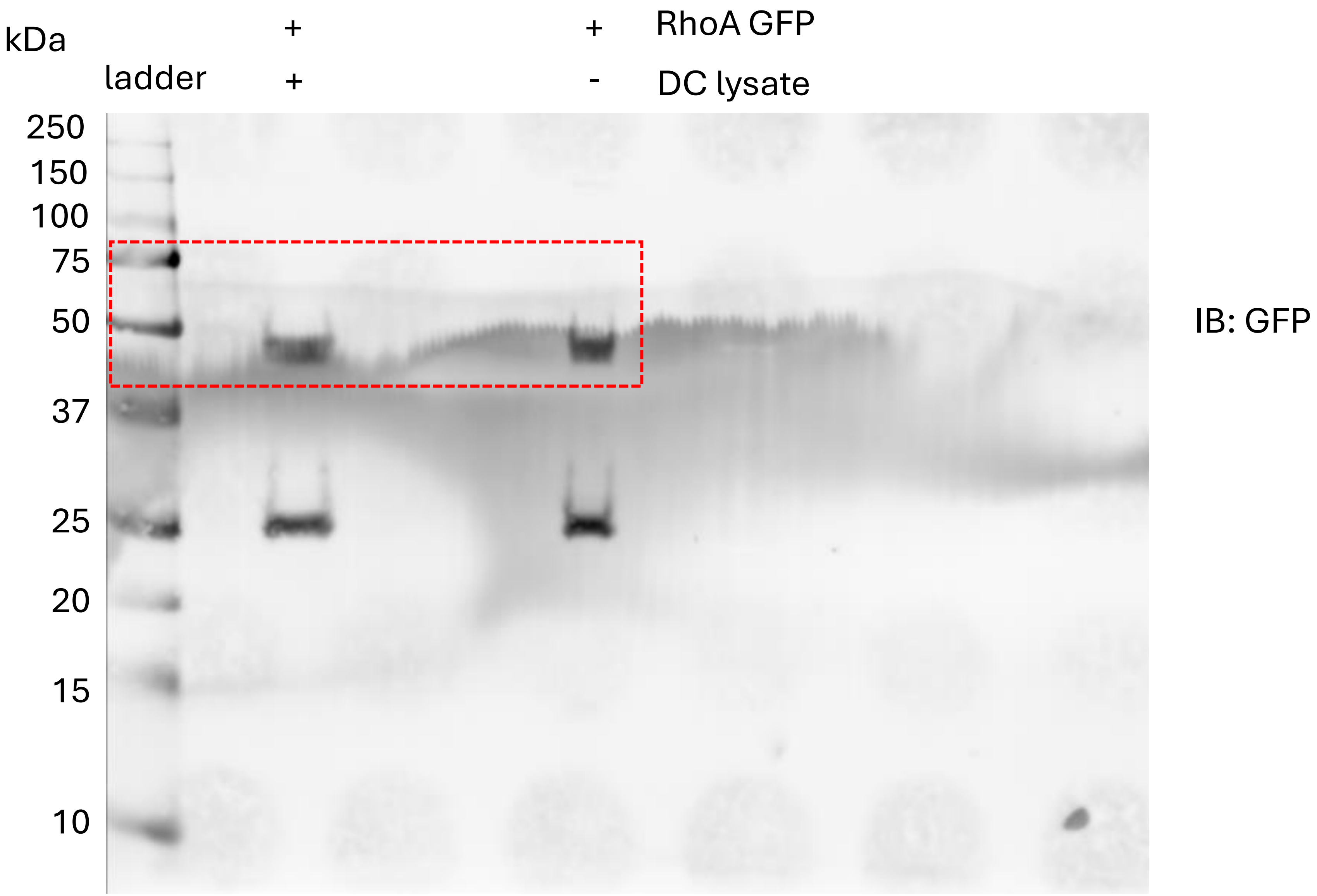
kDa
ladder
+
+
+
-
RhoA GFP
DC lysate
250
150
100
75
50
37
25
20
15
10
IB: GFP
