# Supplementary material for: Galectin-9 regulates dendritic cell polarity and uropod contraction by modulating RhoA activity
Source: J Cell Biol. 2025 Sep 23;224(11):e202404079. doi: 10.1083/jcb.202404079 (PMC12456409; doi:10.1083/jcb.202404079)
Supplement: SourceData FS1 — is the source file for Fig. S1. [file jcb_202404079_sourcedatafs1.pdf]

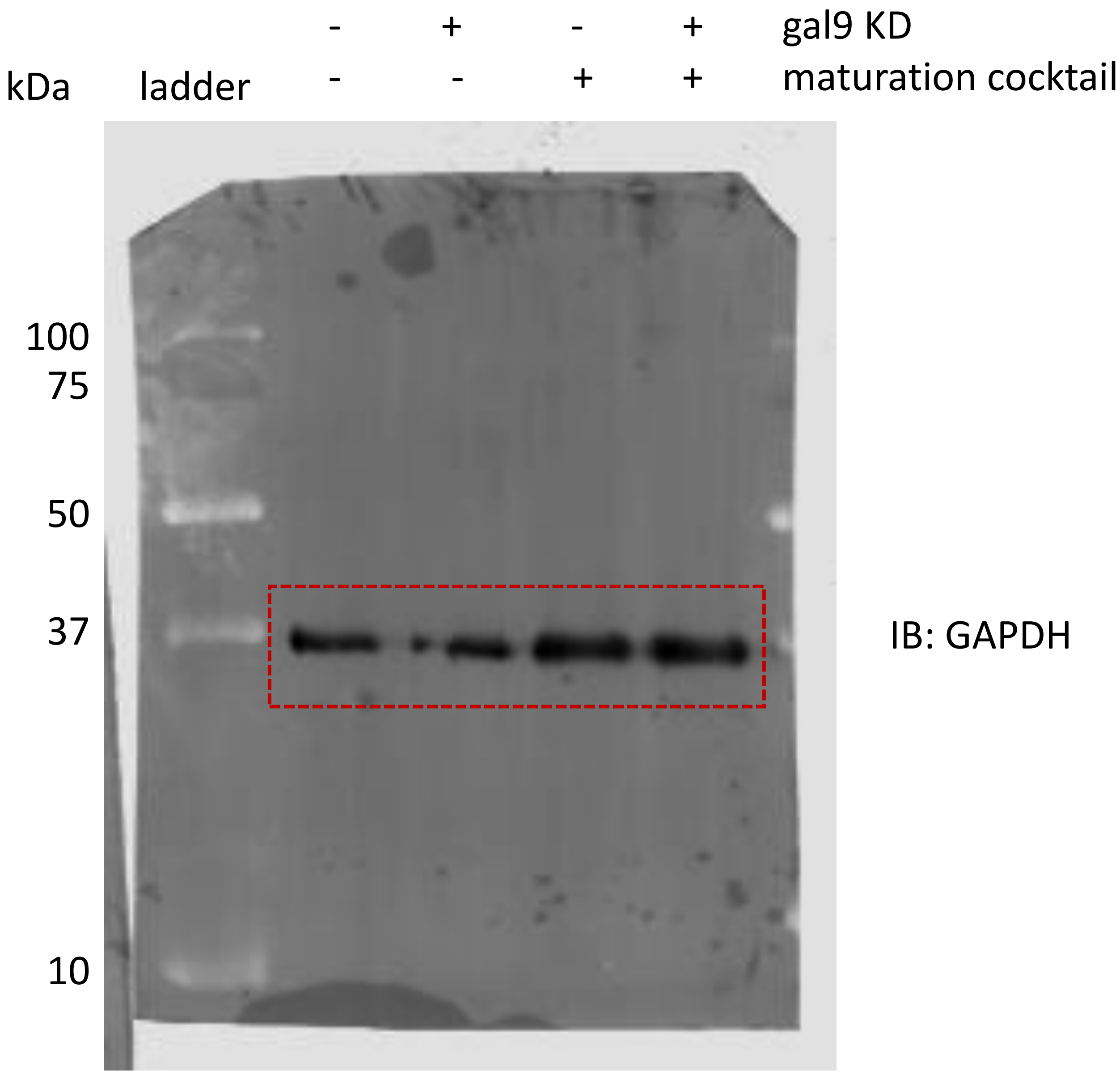
- + - + gal9 KD
kDa ladder - - + + maturation cocktail
100
75
50
37
10
IB: GAPDH

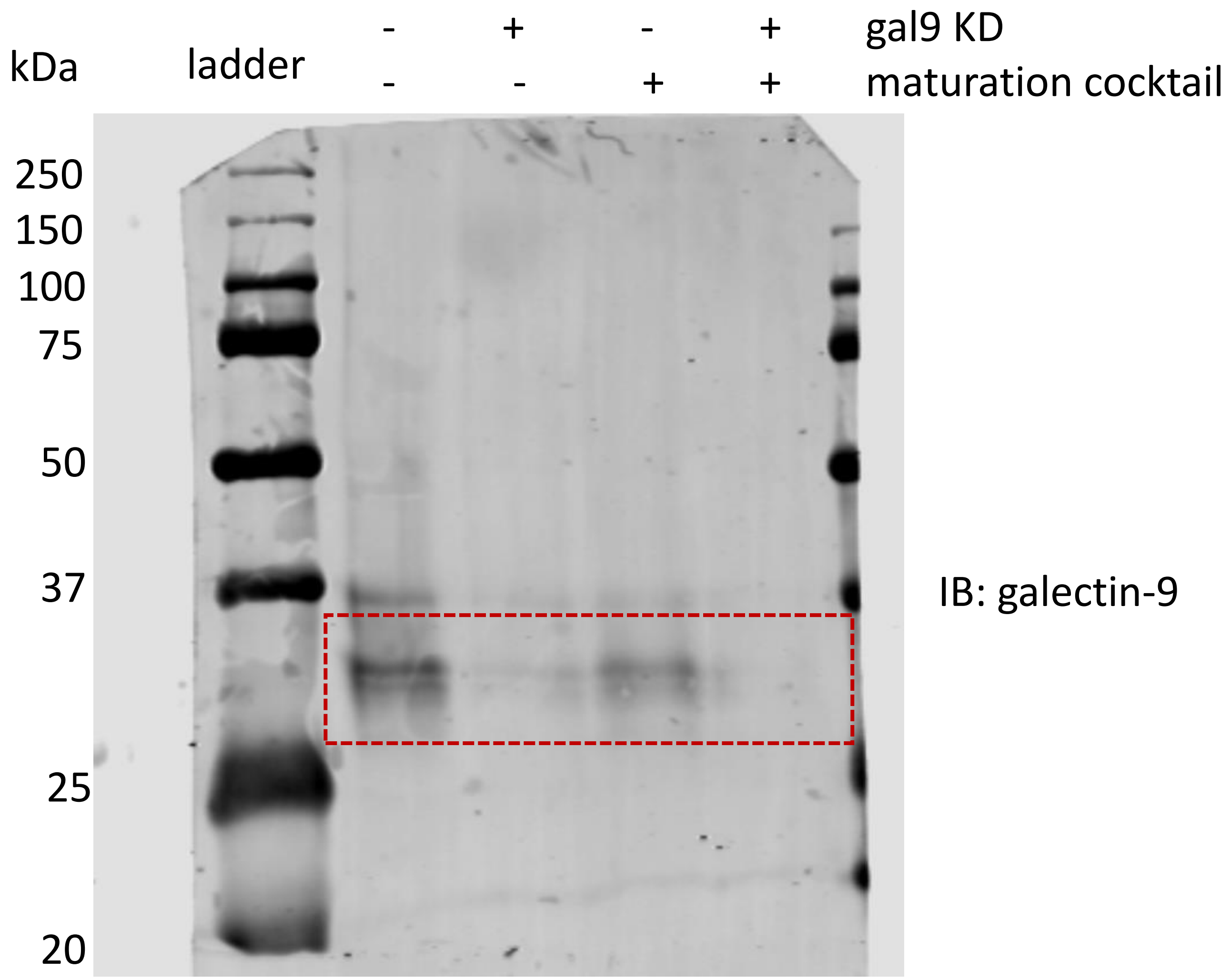
- + - + gal9 KD
kDa ladder - - + + maturation cocktail
250
150
100
75
50
37
25
20
IB: galectin-9
