# Supplementary material for: Galectin-9 regulates dendritic cell polarity and uropod contraction by modulating RhoA activity
Source: J Cell Biol. 2025 Sep 23;224(11):e202404079. doi: 10.1083/jcb.202404079 (PMC12456409; doi:10.1083/jcb.202404079)
Supplement: SourceData FS4 — is the source file for Fig. S4. [file jcb_202404079_sourcedatafs4.pdf]

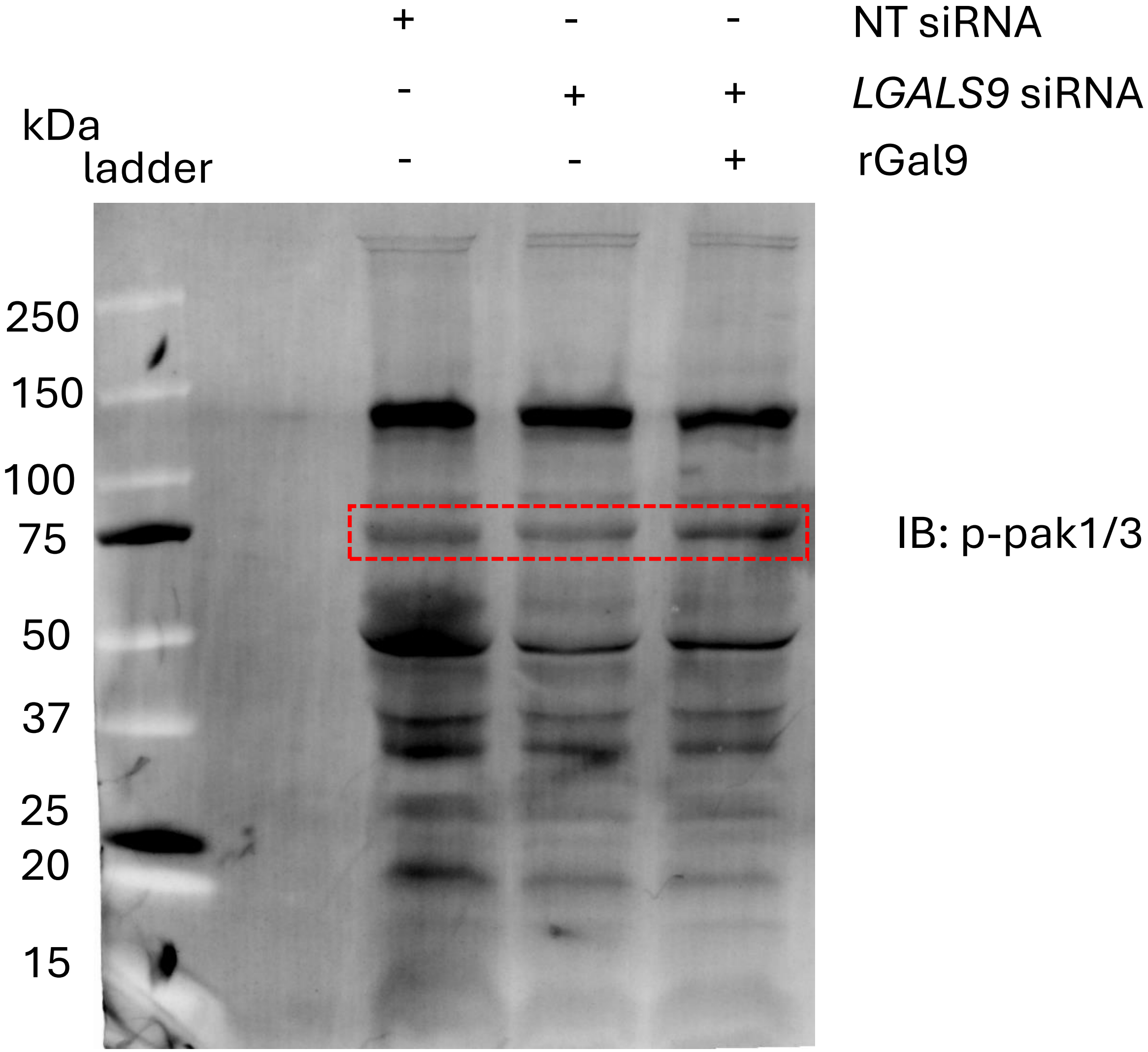

+ - - NT siRNA
- + + *LGALS9* siRNA
kDa ladder
- - + rGal9
250
150
100
75
50
37
25
20
15
IB: p-pak1/3

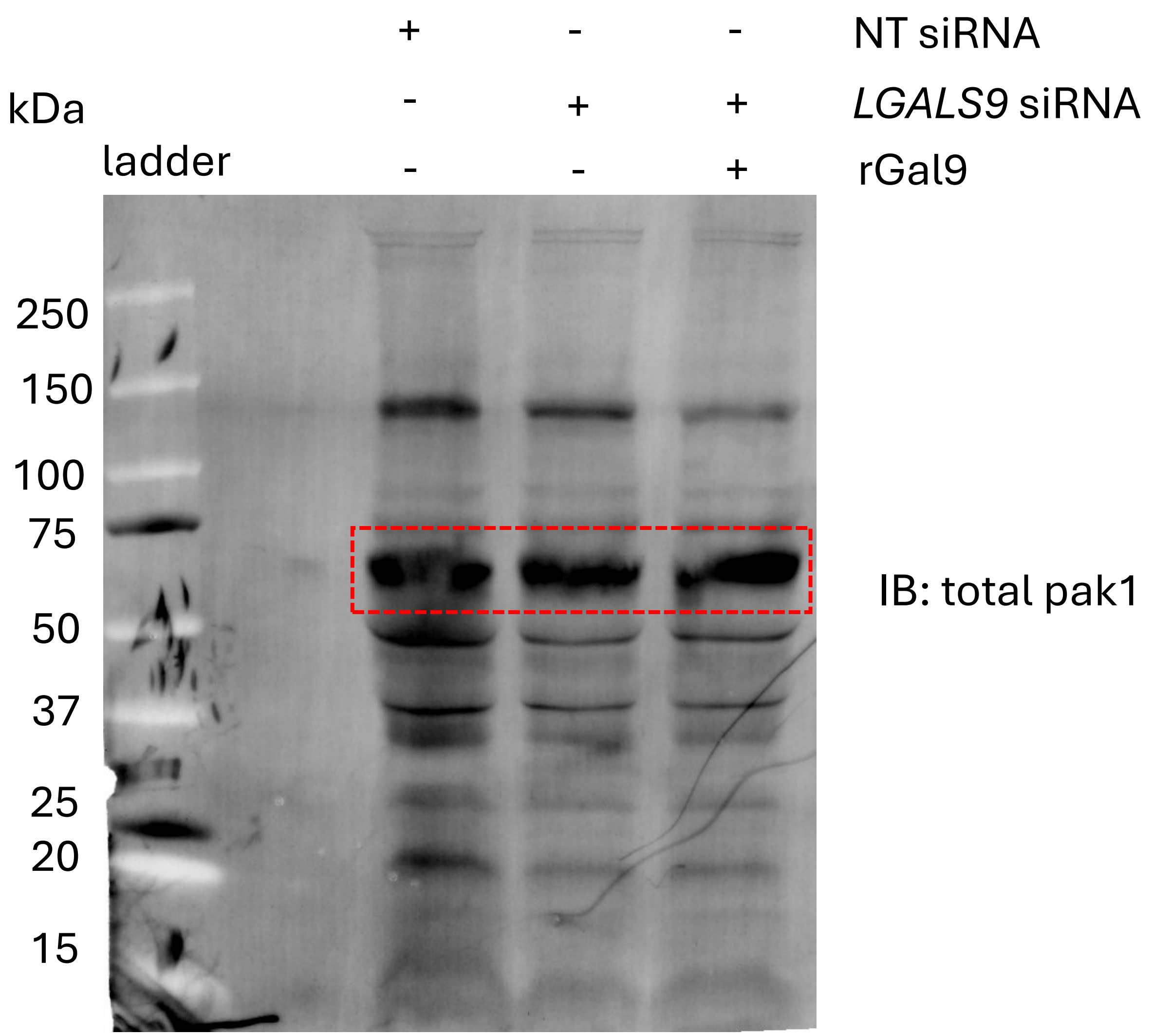

+ - - NT siRNA
kDa - + + LGALS9 siRNA
ladder - - + rGal9
250
150
100
75
50
37
25
20
15
IB: total pak1

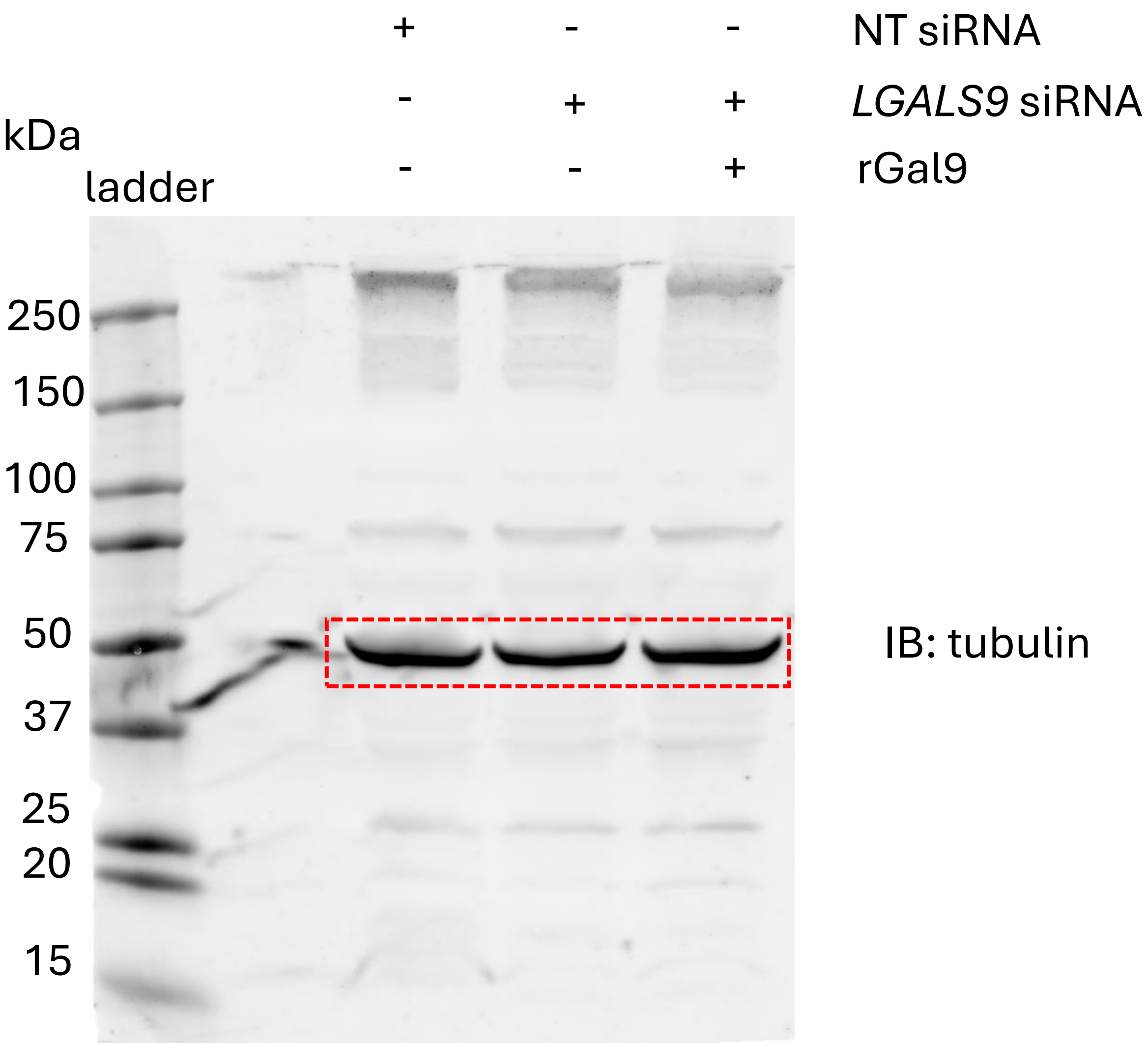

+ - - NT siRNA
- + + LGALS9 siRNA
- - + rGal9
kDa
ladder
250
150
100
75
50
37
25
20
15
IB: tubulin

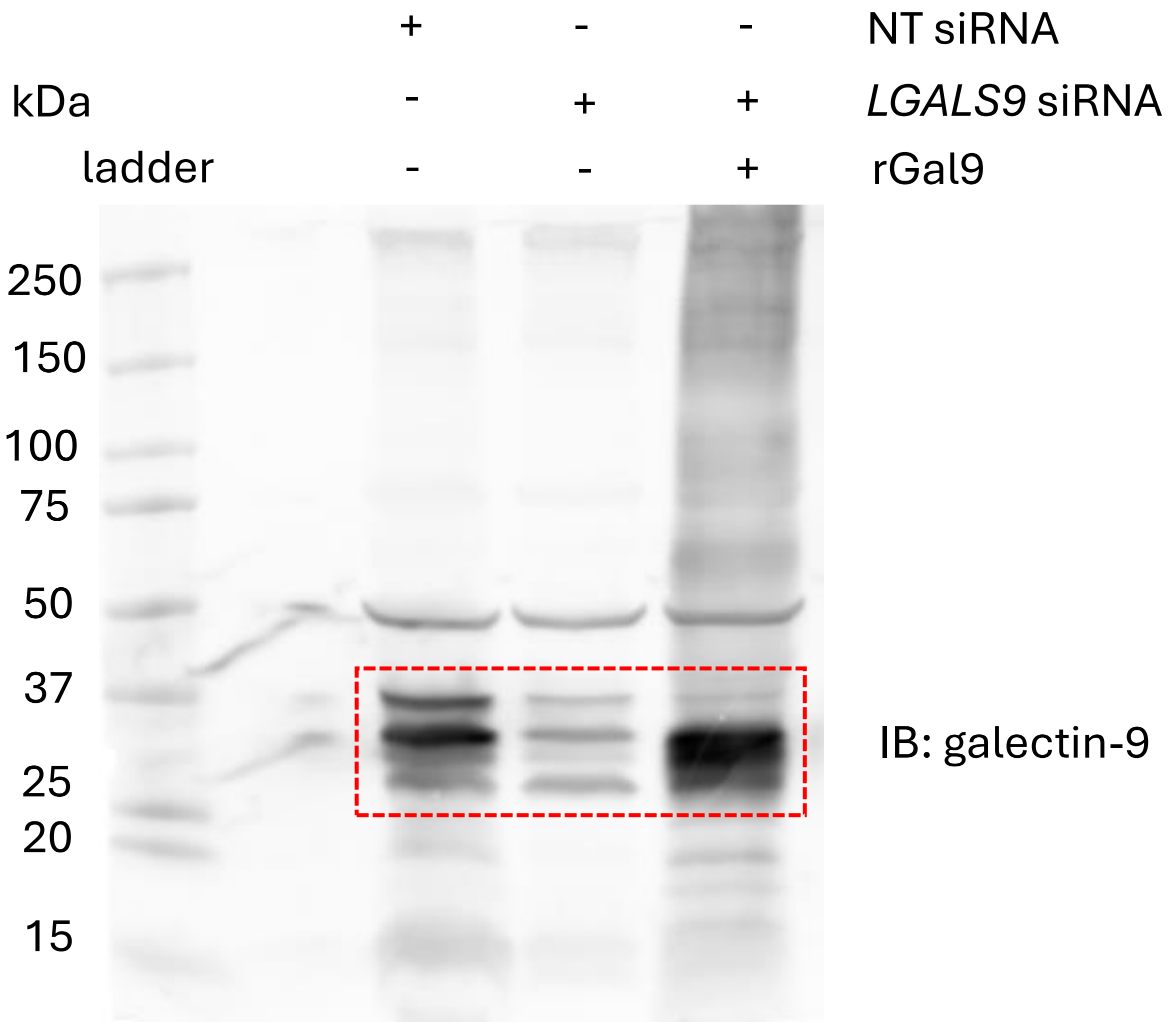
+ - - NT siRNA
kDa - + + *LGALS9* siRNA
ladder - - + rGal9
250
150
100
75
50
37
25
20
15
IB: galectin-9

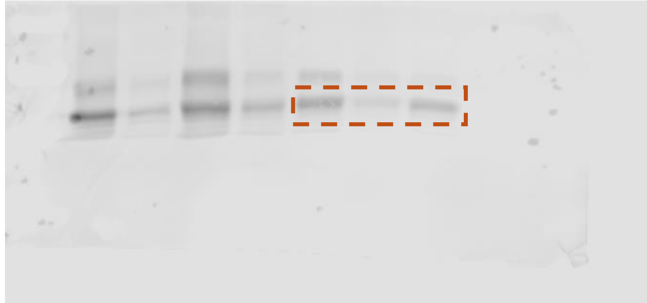
Galectin-9
+ - - WT
- + + gal9 KD
- - + rGal9

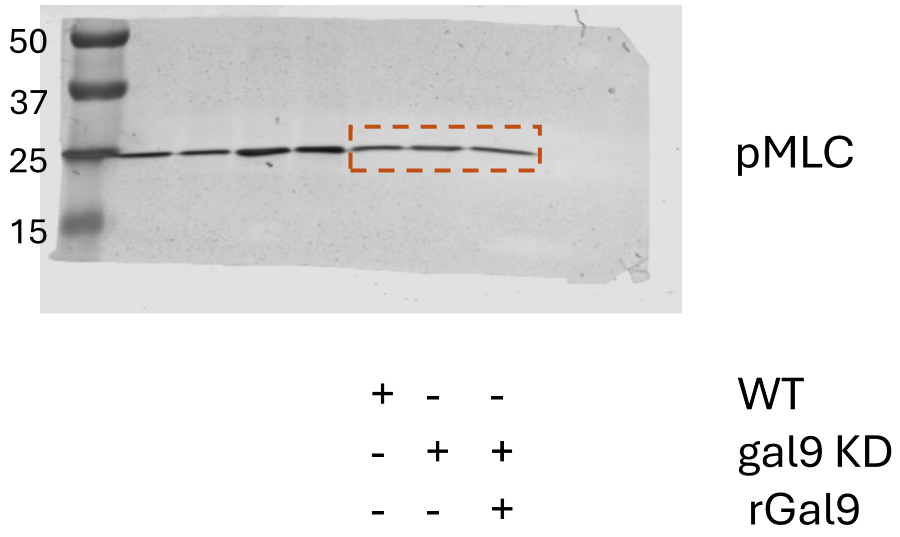
50
37
25
15
pMLC
+ - -
- + +
- - +
WT
gal9 KD
rGal9

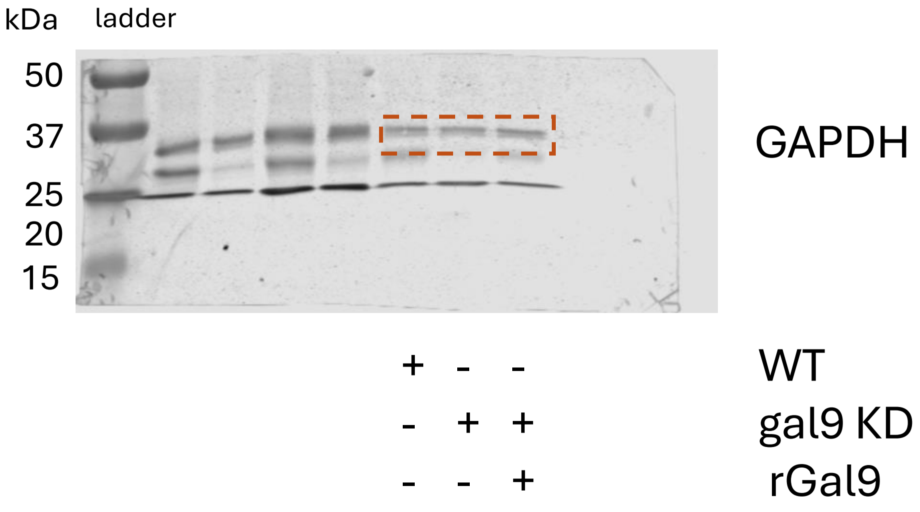

kDa
ladder
50
37
25
20
15
GAPDH
+ - -
WT
- + +
gal9 KD
- - +
rGal9

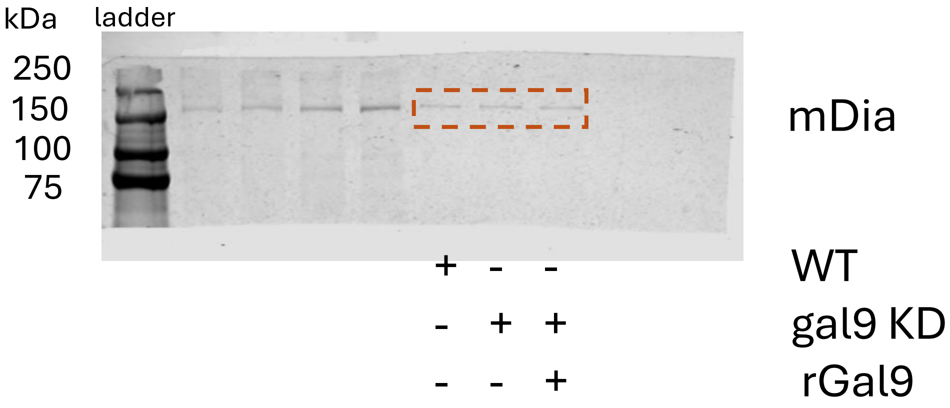
kDa
ladder
250
150
100
75
mDia
WT
+ - -
gal9 KD
- + +
rGal9
- - +
